# PATRI, a Genomics Data Integration Tool for Biomarker Discovery

**DOI:** 10.1155/2018/2012078

**Published:** 2018-06-28

**Authors:** G. Ukmar, G. E. M. Melloni, L. Raddrizzani, P. Rossi, S. Di Bella, M. R. Pirchio, M. Vescovi, A. Leone, M. Callari, M. Cesarini, A. Somaschini, G. Della Vedova, M. G. Daidone, M. Pettenella, A. Isacchi, R. Bosotti

**Affiliations:** ^1^NMS Oncology, Nerviano Medical Sciences Srl, Nerviano, Italy; ^2^University of Milano Bicocca, Milano, Italy; ^3^Icona Srl, Cinisello Balsamo, Italy; ^4^Parametric Design Biotech, Gessate, Italy; ^5^Fondazione IRCCS Istituto Nazionale dei Tumori, Milano, Italy

## Abstract

The availability of genomic datasets in association with clinical, phenotypic, and drug sensitivity information represents an invaluable source for potential therapeutic applications, supporting the identification of new drug sensitivity biomarkers and pharmacological targets. Drug discovery and precision oncology can largely benefit from the integration of treatment molecular discriminants obtained from cell line models and clinical tumor samples; however this task demands comprehensive analysis approaches for the discovery of underlying data connections. Here we introduce PATRI (Platform for the Analysis of TRanslational Integrated data), a standalone tool accessible through a user-friendly graphical interface, conceived for the identification of treatment sensitivity biomarkers from user-provided genomics data, associated with information on sample characteristics. PATRI streamlines a translational analysis workflow: first, baseline genomics signatures are statistically identified, differentiating treatment sensitive from resistant preclinical models; then, these signatures are used for the prediction of treatment sensitivity in clinical samples, via random forest categorization of clinical genomics datasets and statistical evaluation of the relative phenotypic features. The same workflow can also be applied across distinct clinical datasets. The ease of use of the PATRI tool is illustrated with validation analysis examples, performed with sensitivity data for drug treatments with known molecular discriminants.

## 1. Introduction

The recognition of cancer as a genetic disease has raised in recent years huge “omics” efforts that have generated extensive molecular information on cancer cell lines and tumor samples, along with clinical characterization and drug sensitivity information. These data are often accessible through public resources, such as CCLE [1, 2], TCGA Research Network [3], ExpO [4], and ICGC [5], to name a few. As a consequence, a number of initiatives, often at global scale, have taken advantage of this unprecedented opportunity, such as the Cancer Therapeutics Response Portal (CTRP) [6–8], linking publicly available cancer cell line features to small-molecule sensitivity for the discovery of patient-matched cancer therapeutics, or the i2b2 (Informatics for Integrating Biology and the Bedside)-tranSMART Foundation, a platform and a community aimed at integrating clinical and translational research data, providing “open-source, open-data” resources for precision medicine [9, 10]. In translational research, treatment sensitivity biomarkers are key to decision-making, for the identification and definition of patient populations susceptible to therapy benefits. In recent years, the search for biomarkers has indeed raised a huge community effort [1–3, 11–15] and a stimulating debate around the drug sensitivity issue [16–20]. Cancer cell lines can recapitulate many of the molecular alterations driving tumor drug sensitivity [11]: for this reason, molecular characterization of experimental preclinical models has been widely used in support to all phases of drug discovery and development, for the identification of potential targets and for the exploration of several molecular aspects, such as drug sensitivity contexts, mechanisms of action, or issues in treatment responsiveness. Correlation between multiple baseline cancer genomics data and relative drug sensitivity has been explored in a number of public resources, such as CellMiner [12, 21], Genomics Drug Sensitivity in cancer (GDSC) [13, 22], or CancerDP [23, 24], where data can be enriched for compound and/or cell line numerosity but cannot be extended to user-supplied genomics or compound sensitivity data, which would be fundamental for new drug development.

Biomarkers resulting from the complex task of complementing cancer preclinical findings with clinical knowledge have found application as prognostic or diagnostic indicators, favoring the design of companion diagnostics for targeted drugs and facilitating therapeutic developments [28]. This is the case, for instance, of* KRAS* gene mutations in the observed resistance to cetuximab and panitumumab treatment in colorectal cancers samples [29, 30] or of rearranged Abl in the sensitivity to imatinib in acute myeloid leukemia [31] and of afatinib, gefitinib, and erlotinib in* EGFR* mutated tumors [32, 33]. Other examples are the sensitivity to vemurafenib in* BRAF* mutated tumors [34] or to lapatinib in amplified/overexpressed Her2 (ERBB2) positive tumors [35], as well as the sensitivity to inhibitors of ALK, ROS1, and TRK (*NTRK1-2-3*) in tumors harboring activating rearrangements of these kinases [36, 37]. Indeed, gene rearrangements with kinase catalytic domains often result in the anomalous overexpression of kinase targets, driven by the partner gene, which can be identified by protein or RNA expression analysis as indirect readout [38, 39]. Especially in the targeted drug field, determination of patient eligibility for a certain treatment is sometimes only possible by performing a specific molecular assay on clinical specimens; however, other peculiar phenotypic characteristics measured in treatment susceptible individuals might be used to aid in the process of patient population selection. An example is the frequent association of the presence of ALK rearrangements in lung tumors with patients' young age, male gender, and nonsmoking history [40, 41].

The aim of our work was to provide a flexible and quick solution to streamline an analysis workflow for the search of potential treatment biomarkers across preclinical and clinical datasets and to make it accessible for application to user-provided genomics and treatment sensitivity data. For this purpose, we designed the “Platform for the Analysis of TRanslational Integrated data” (PATRI) tool, requiring data imported by users and integrating this workflow with an intuitive user-friendly graphical interface. First, drug response information is required to set up statistical analyses for the identification of potential drug sensitivity baseline genomic traits in cell lines (“Translational” workflow) or in tumor samples (“Clinical” workflow); lists of resulting relevant markers can then be used to predict genomics responsiveness in annotated datasets of tumor samples, which are stratified into putative “Sensitive” or “Resistant” populations by the algorithm and correlated with the respective relevant clinical characteristics.

PATRI is conceived for basic end-users and is freely distributed as a virtual machine, portable on Windows, Linux, and MacOS platforms. The PATRI tool is accessible for open download at https://www.parametricdesign.bio/.

## 2. Materials and Methods

### 2.1. PATRI Database and Structure Implementation

In PATRI, the Database Management System (DBMS) implementation was based on MariaDB. The database schema (Suppl. Fig.  1) was designed to include cross-referenced tables for Cell Line and Clinical Sample genomics data (gene expression, somatic mutation, and copy number) and respective sample annotations, each with fixed and customizable fields. Upload of data input was predisposed as tab-delimited text files, previously formatted to mirror the destination tables. Detailed descriptions and instructions can be found in the* PATRI Platform User's Guide* (Suppl. File) and in the* PATRI Platform Installation and Configuration Guide* (Suppl. File) downloadable documents. Export of analysis results was enabled as tab-delimited text files. All PATRI functions were made accessible through a web graphical user interface developed using Joomla and Zend Frameworks. Statistical analysis libraries from CRAN [42] and Bioconductor R [43, 44] were integrated and interactive graphing functions were introduced using Google Charts [45]. PATRI is provided for download at https://www.parametricdesign.bio/ as an Oracle VM Virtual Box file, populated with an artificial demonstration dataset, which can be removed and replaced with the desired data following instructions in the PATRI Platform User's Guide (Suppl. File).

### 2.2. Statistical Methods

The PATRI tool integrates selected libraries from CRAN [42] and Bioconductor R [43] for the statistical analysis of molecular data, according to the different genomics data types. Before statistical analysis of defined “Sensitive” versus “Resistant” sample groups, complexity reduction of the input genomics data is performed via a stepwise filtering procedure to remove background noise, i.e., all genes with no significant variation across samples. Briefly, all the genes appearing constant across conditions are removed from the data (i.e., never mutated or always mutated genes; all genes with identical “loss”, “normal”, or “gain” copy number type definition across samples; all genes with log2 expression below a user-selectable threshold value). In addition, a calculation of a point-biserial Pearson's correlation between sensitive/resistant cell lines or clinical samples and each gene is also applied, followed by removal of poorly correlated genes (default absolute value cut-off: 0.1).

For all accepted data types, i.e., gene expression, copy number, and mutation, a custom implementation of the Elastic Net algorithm [46] originally used in Barretina J et al. [1] was included, applicable if both “Sensitive” and “Resistant” groups are composed of a minimum of 4 samples each. The Elastic Net is a regularization and variable selection method favoring the selection of strongly correlated predictors, particularly useful for data matrices in which the number of features (genes) is much bigger than the number of subjects (samples). The relative robustness of a selected gene is represented by the final ranking, reported as the percentage of times a molecular feature is retained in the model across 100 runs, accompanied by the average beta value across runs. Additionally, for the detection of differentially expressed genes, we introduced testing procedures such as ANOVA and Limma [47], particularly suited for small sample groups [48]; ANOVA was included also for copy number analysis. Resulting p values and log2 fold change measures are reported and used to rank the molecular features. For the detection of mutated genes, statistical tests based on hypergeometric distribution and odds ratio measures were also implemented. The above algorithms can be applied starting from cell line genomics data (“Translational” workflow) or from tumor sample genomics data (“Clinical” workflow).

Buttons and slide bars are provided to enable sorting and manual filtering of the obtained gene lists, differentiating “Sensitive” and “Resistant” cell lines or clinical samples, based on statistical significance or fold change differences. Putative “biomarker” gene lists can then be quickly evaluated in the available annotated clinical sample data to categorize them into predicted “biomarker sensitive” or “biomarker resistant” cases and to extract relevantly differentiating clinical descriptive parameters in a single button click. First, a reversed classification algorithm based on “random forest” [49] is launched, applying a majority voting approach to assign clinical samples to the most likely category (“Sensitive” or “Resistant”), based on the status of the candidate biomarkers of the starting filtered gene list. One hundred thousand classification trees are run in parallel, using the entire spectrum of identified biomarkers for the random forest classification model. Then, the resulting “Sensitive” and “Resistant” assigned clinical samples are immediately tested for association with specific patient clinical annotations with a two-tail nonparametric Mann–Whitney test for continuous variables (like age, number of cigarettes, etc.) and a multiple-category Chi-square test for categorical variables (tumor subtype, grade, etc.). Associated clinical features are displayed in a table, ranked based on significance p values: visualization of each tested variable is enabled as a boxplot or a barplot, respectively. A heatmap, clustered both on molecular features and on samples, is reported with clustering distances calculated with Hamming distance for mutations and with Ward's method for copy number and gene expression. For available time-to-event survival data, a separate classical two-tail log-rank test between predicted sensitive and resistant samples can be run, with visualization via Kaplan-Meier survival curves.

### 2.3. Cell Line Compound Treatment

Cell lines were grown in the appropriate culture media as recommended by vendors and treated with increasing doses of the tested compounds. Drug sensitivity data were expressed as the micromolar concentration of the compound at which cell proliferation is reduced by 50% (IC50). All cell lines were authenticated by STR analysis (AmpFlSTR® Identifiler® PCR Amplification Kit, Applied Biosystems, Foster City, CA, USA) using the GeneMarker HID v 2.4.0 software (Soft Genetics, State College, PA, USA) and comparative analysis was performed with CLIFF (Cell Line Identity Finding by Fingerprinting, [50]).

### 2.4. Datasets and Analysis Workflows for PATRI Validation

Genomics data for 1036 cell lines were imported from CCLE [51]; mutation data were converted into binary information (wild type or mutated genes); cell line compound sensitivity was assessed in-house. Annotated TCGA clinical genomic datasets [3], comprising gene expression, copy number, mutation data, and clinical sample descriptions, were obtained from cBioportal [52, 53] for breast cancer (Breast Invasive Carcinoma (TCGA, “Provisional”), 1017 samples) and melanoma (Skin Cutaneous Melanoma (TCGA, “Provisional”), 478 samples); none of the datasets contained treatment response information for the considered drugs. Two lymphoma gene expression datasets for 20 (GSE14879) and 130 (GSE19069) samples, respectively, were downloaded from Gene Expression Omnibus (GEO) [54], with clinical annotations derived from the respective descriptive publications [25, 26]; the two datasets were not merged due to the discrepancy of the available clinical sample information and were utilized to test the “Clinical” analysis workflow. Txt tables were created with cell line names and the respective “Sensitive” or “Resistant” labels, assigned based on a threshold IC50 of 1 *μ*M for all the tested compounds. For the lymphoma GEO14879 clinical dataset, no entrectinib treatment response could be available, so drug sensitivity was presumed for the 5 ALK-positive samples, arbitrarily defined as “Sensitive” responders to ALK inhibition only to simulate a “Clinical” workflow analysis. Statistical analysis was launched on the selected cell lines or clinical samples using all the algorithms in PATRI for all the available genomics data types; only the relevant molecular signature results, filtered based on the indicated p value and/or log fold change thresholds, are discussed in the manuscript. The filtered lists were then used to categorize the indicated clinical samples into “Sensitive” and “Resistant” and to explore the resulting statistically relevant sample annotations, ranked based on significance (p value).

## 3. Results and Discussion

### 3.1. Design and Implementation of PATRI

**Figure 1:**
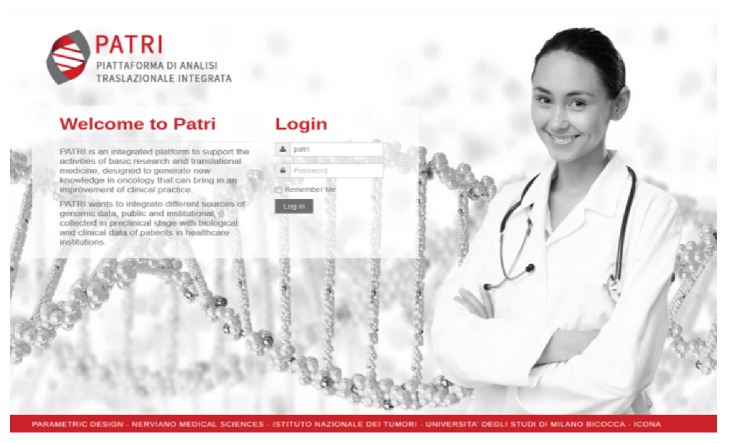
**PATRI graphical user interface: welcome and login page. **Screenshot of the PATRI welcome and login home page. See also text and Supplementary File.

The identification of sensitivity markers implicated in cancer treatment response is fundamental to support patient population definition in the clinics and is well established for a number of approved kinase inhibitors drugs that are selectively active in tumors harboring activating mutations or rearrangements of their target genes, such as vemurafenib in BRAF mutated tumors [34], lapatinib in amplified/overexpressed ERBB2 (Her2) positive tumors [35], and entrectinib in ALK rearranged tumors [37]. We focused on building an intuitive tool for use in drug discovery pipelines to immediately link relevant molecular markers from cell line drug treatment models with clinical features associated with tumor sample genomics data, for the quick exploration of potential population therapeutical biomarkers. For this purpose, we have developed PATRI (Platform for the Analysis of TRanslational Integrated data), an open-source tool offering a flexible genomic data integration resource to basic end-users for the identification of predictive biomarkers of differential sensitivity to drugs or any other treatments, such as siRNA or CRISPR-Cas9, starting from user-provided data. Central to the design was the ease of use, through an intuitive graphical user interface, based on a simple workflow of streamlined data analysis, extraction, and visualization procedures, directly correlating biomarkers identified in cell line or tumor sample genomics data to clinical information, aided by the introduction of mouseover and pop-up interactive options. PATRI is a web-based application (Figure 1) with a client-server architecture, as detailed in the PATRI Installation and Configuration Guide (Suppl. File), built on a relational database supporting data mining activities. The downloadable tool is initially populated with a “test” database for demonstration purposes that can be removed and replaced with the desired data. Free codes and analysis packages were utilized for the implementation of all PATRI components to enable distribution as an open-source tool and, possibly, custom code implementation.

**Figure 2:**
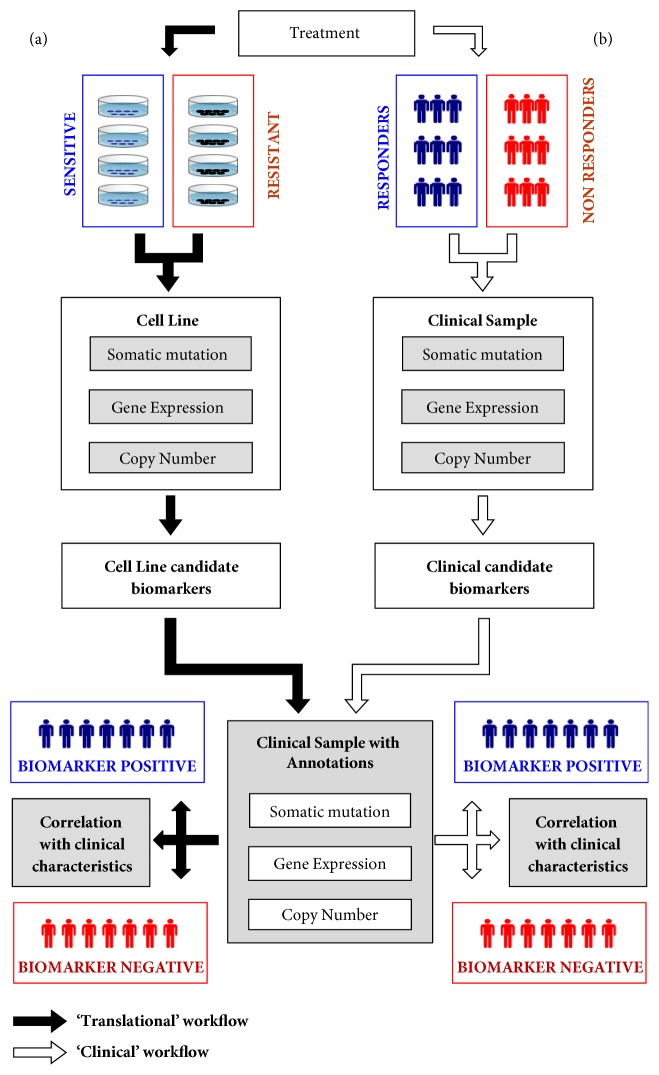
**PATRI analysis workflows.** Schematic representation of the PATRI tool analysis workflows. (a) “Translational” workflow (black arrows), executing a statistical identification of candidate baseline genomics biomarkers starting from defined treatment “Sensitive” versus “Resistant” cell line groups, through categorization of “biomarker sensitive” versus “biomarker resistant” clinical samples in the annotated clinical dataset, based on the selected gene candidates, with simultaneous identification of statistically correlated differentiating characteristics. (b) “Clinical” workflow (white arrows), executing the same operations as in the “Translational” workflow, starting from defined treatment “Sensitive” (responder) versus “Resistant” (nonresponder) tumor samples in pretreatment clinical genomics datasets.

Figure 2 schematically illustrates the conceptual “Translational” and “Clinical” workflows in PATRI, with full functionalities detailed in Suppl. Fig.  2-7 and in the PATRI Platform User's Guide (Suppl. File). A “Translational” workflow is available to obtain putative treatment biomarkers starting from cell line genomics data that can be used to categorize clinical samples into “biomarker sensitive” and “biomarker resistant” samples and to simultaneously obtain significantly correlated clinical characteristics for patient stratification (Figure 2(a)). Similarly, starting from clinical genomic datasets (“Clinical” workflow, Figure 2(b)), PATRI allows analysis and correlation of putative treatment response genomics markers from a test tumor sample population to the clinical characteristics of a second clinical sample cohort. Briefly, after import of the desired baseline (pretreatment) genomics data for cell line models and clinical samples, users will define opposite groups of “Sensitive” and “Resistant” cell lines or “responder” and “nonresponder” tumor samples based on available treatment sensitivity information. PATRI automatically retrieves and associates the genomics data and descriptions to the list of provided samples. By a mouse click, single or multiple predefined statistical tests can be chosen and launched for analysis of the selected sets of gene expression, copy number, and gene variant analyses data (Suppl. Fig.  3-4), including the Elastic Net option [1, 46] for all the three types of data. The resulting lists of significant sensitivity biomarker genes are displayed in separate tabs with sorting buttons and slide bars, allowing data filtering; the tool also enables quick export of results and graphical visualization through different charting options supporting mouseover and zooming functions (Suppl. Fig.  5), such as interactive Volcano plots, dendrograms, or scatter plots integrating data by color shades and dot sizes. Filtered sensitivity biomarkers obtained at this point for cell lines can be immediately connected to clinical data imported into PATRI, to investigate the presence of molecularly discriminated clinical subpopulations: one mouse click starts simultaneous classification of clinical tumor samples, based on the respective molecular status of the selected filtered biomarkers, as potentially “Resistant” or “Sensitive” to the drug, via an adaptation of the random forest classifier algorithm [49], together with a stratification of associated clinical sample characteristics, ranked based on statistical significance (Suppl. Fig.  6-7). Graphical representation and data export upon mouse clicking allow exploration of the identified clinical features associated with tumor genomics data (Suppl. Fig.  7) and permit rapid identification of particularly discriminating clinical features potentially defining patient subpopulations, which might be used in support of patient selection for clinical trials. Thanks to the flexibility of sample description fields in the PATRI database, along with cell lines or clinical samples, the tool might similarly accept data from patient-derived cancer models, such as PDXs and PDOs (patient-derived xenografts and organoids, respectively) that more closely mirror the architecture and cellular heterogeneity of human tumors [55–57], increasingly available with associated clinical/genomic data sets and annotations thanks to a number of recent international initiatives (e.g., Human Cancer Model Initiative (HCMI) [58], EurOPDX Consortium [59], or Public Repository of Xenografts (PRoXe) [60], to name a few) [61–63].

### 3.2. Validation of PATRI

For the validation of the tool, we generated in-house cell growth inhibition sensitivity data (IC50) on panels of cancer cell lines treated with the well-known targeted drugs lapatinib, vemurafenib, or entrectinib and tested PATRI for the ability to identify significant treatment sensitivity-related molecular markers through the “Translational” workflow (Figure 2(a)), using data from CCLE [1, 51], TCGA [3], and Gene Expression Omnibus (GEO) [54] resources.

**Figure 3:**
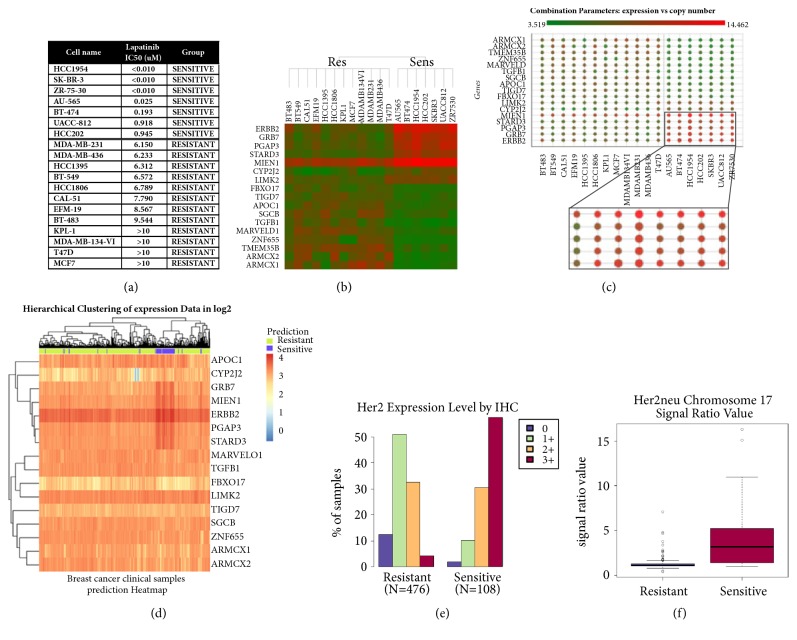
‘‘**Translational” analysis of lapatinib sensitivity in breast cancer cell lines and clinical samples.** Results of PATRI “Translational” analysis workflow performed on a panel of breast cancer cell lines sensitive or resistant to lapatinib treatment and on a panel of 1017 TCGA breast cancer clinical samples. (a) List of treated breast cell lines with respective lapatinib IC50 values. A threshold of 1*μ*M was chosen to define “Sensitive” and “Resistant” cell lines. (b) Heatmap dendrogram of 17 markers differentiating lapatinib sensitive versus resistant breast cell lines, obtained by ANOVA gene expression analysis (p value>10^−4^, log2 FC>|1.5|). Significantly high or low expressed genes are highlighted in red and green, respectively. (c) Scatter plot visualization of the identified genes in the different cell lines, combining dot size, representing magnitude of copy number values, and dot color shades, ranging from low (green) to high (red) gene expression values. (d) Heatmap representing hierarchical cluster analysis via random forest categorization of the predicted “Sensitive” or “Resistant” 1071 breast cancer samples, based on the selected genes (with the exception of* TMEM35B*, not represented in the clinical dataset; sample IDs could not be represented on the lower part of the graph). (e-f) Top ranking of significant clinical features (where available) associated with TCGA breast cancer samples, classified as potentially “Sensitive” or “Resistant”. (e) Histogram representing distribution of clinically evaluated Her2 immunohistochemistry levels (0-3+) in the predicted “Sensitive” and “Resistant” clinical sample groups. The displayed data correspond to the column “Her2 IHC score” in the TCGA Breast Invasive Carcinoma “Provisional” Clinical Data annotation file. (f) Box plot representing the clinically assessed average signal value for chromosome 17 amplification in predicted “Sensitive” (4.28) and “Resistant” (1.32) clinical sample groups. The displayed data correspond to the column “Her2 cent 17 ratio” in the TCGA Breast Invasive Carcinoma “Provisional” Clinical Data annotation file.

Lapatinib [35] is a dual EGFR and ERBB2 inhibitor, currently approved in the clinics for the treatment of* ERBB2* amplified breast cancers in combination with capecitabine or letrozole [64]. In our analysis,* ERBB2* kinase gene amplification and overexpression were correctly identified by PATRI within a group of lapatinib sensitive versus resistant breast cancer cell lines (Figure 3(a)) tested in our labs. Concomitant amplification and overexpression of a number of additional genes, correlating with lapatinib treatment sensitivity, were also observed (Figures 3(b) and 3(c)). Many of these genes, such as* GRB7*,* PGAP3*,* STARD3*, and* MIEN1,* have been reported to be coamplified and overexpressed with* ERBB2 *in breast tumors in the “*ERBB2* amplicon”, located on the long arm of chromosome 17 (17q12), neighboring the* ERBB2* coding sequence [65–68]. STRING analysis [27] of the 17 differentially expressed genes in Figure 3(b) (obtained by ANOVA expression analysis, p value>10^∧^-4, log2 FC> |1.5|) revealed a considerable number of known or predicted protein interactions, supporting the functional interconnections in the selected list (not shown). Using the “Translational” workflow implemented in PATRI, the above marker list was used to categorize potentially “Sensitive” or “Resistant” cases in a panel of breast cancer clinical samples from TCGA data collection with the respective associated clinical feature annotations via random forest classification. In the resulting breast sample hierarchical analysis heatmap, most of the predicted “Sensitive” breast cancer samples were clustered in a compact group (Figure 3(d)), characterized by a strong enrichment in Her2 positive tumors as measured by immunohistochemistry (IHC levels = 3+) (Figure 3(e)) having more than 90% cells positive to Her2 staining (not shown), both characteristics clearly associated with ERBB2 overexpression. In addition, chromosome 17 amplification (chromosome 17 signal ratio value) was also among the top ranking clinical annotations differentiating predicted “Sensitive” and “Resistant” samples in the breast cancer dataset (Figure 3(f)). Gene lists obtained from gene expression or copy number alternative analysis algorithms (ANOVA and Elastic Net for both copy number and gene expression, Limma for gene expression) and with different filtering thresholds could all identify groups of “Sensitive” breast cancer samples significantly enriched in Her2 IHC-positive tumors and with marked chromosome 17 amplification among the top ranking clinical reported features in the breast cancer dataset. Interestingly, this result was observed also with the Elastic Net copy number list (not shown), which did not include* ERBB2* among the most significant differential genes. This observation prompted us to test the robustness of the obtained gene signatures after removal of the* ERBB2* gene from all the previously evaluated biomarker gene lists. Though with a lower p value, the predictive power was still retained, with a strongly significant enrichment in Her2 IHC-positive and chromosome 17 amplified samples among the predicted “Sensitive” (not shown), likely driven by the other overexpressed and amplified genes from the “ERBB2 amplicon” included in the signature.

**Figure 4:**
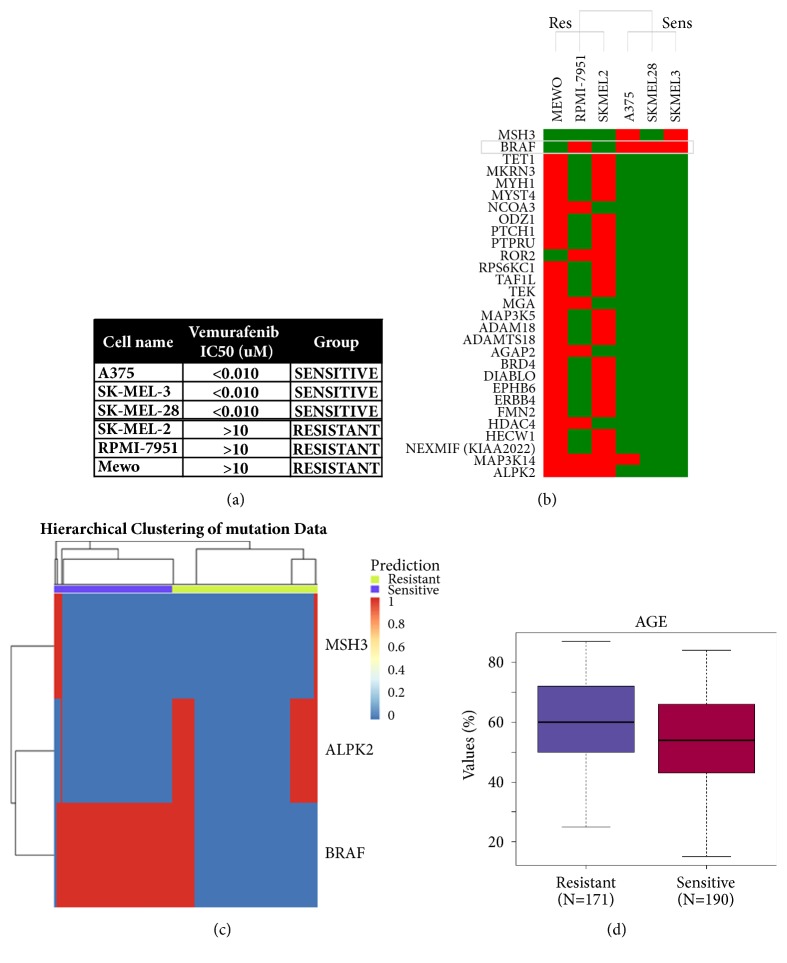
‘‘**Translational” analysis of vemurafenib sensitivity in melanoma cell lines and clinical samples. **Results of PATRI “Translational” analysis workflow performed on a panel of melanoma cell lines sensitive or resistant to vemurafenib treatment and on a panel of 478 TCGA melanoma clinical samples. (a) List of melanoma cell lines with respective vemurafenib IC50 values. A threshold of 1*μ*M was chosen to define “Sensitive” and “Resistant” cell lines. (b) Heatmap dendrogram for the results of PATRI odds ratio mutational analysis in “Sensitive” versus “Resistant” melanoma cell lines. Red, mutated genes; green, wild type genes. (c) Heatmap representing hierarchical cluster mutation analysis via random forest categorization of the predicted “Sensitive” or “Resistant” 478 melanoma TCGA samples, based on 3 selected mutated genes:* BRAF, MSH3, ALPK2* (sample IDs could not be represented on the lower part of the graph). (d) Box plot representing the reported age distribution in predicted “Sensitive” (avg. 54.3) and “Resistant” (avg. 60.3) clinical melanoma sample groups. The displayed data correspond to the column “Age” in the TCGA Skin Cutaneous Melanoma (TCGA, Provisional) Clinical Data annotation file.

Vemurafenib (Zelboraf) is a B-Raf inhibitor approved for the treatment of late-stage melanoma. It selectively inhibits melanoma cells harboring the V600E* BRAF* activating mutation, being inactive on WT* BRAF* cells [34]. Using the PATRI workflow, statistical mutation analysis in a small panel of melanoma cell lines showing differential sensitivity to vemurafenib (Figure 4(a)), provided a list of 29 mutated genes (filtered p value>0.1, log10 odds ratio> |1|), among which mutated* BRAF *was the only feature common to the 3 highly sensitive melanoma cell lines, but also present in the resistant RPMI-7951, harboring a V600E* BRAF* mutated gene (Figure 4(b)). This cell line has been previously described as a B-Raf inhibitor resistant cell line [69], likely due to a reactivation of the MEK pathway, in which a combined treatment with the AS703026 MEK inhibitor and the PLX4032 BRAF inhibitor could actually overcome this resistance phenotype [69]. In 2 out of 3* BRAF *mutated sensitive cell lines, we concomitantly observed a mutation in MutS Homolog 3 (*MSH3*), a gene participating in the mismatch repair (MMR) system. Indeed,* BRAF* mutations have been observed to frequently occur in colorectal tumors cases with MSI characterized by deficient DNA mismatch repair (dMMR) [70]. Besides, we found the* ALPK2* kinase to be preferentially mutated in* BRAF *wt-vemurafenib resistant melanoma cell lines; mutations in* ALPK2* have been proposed to be involved in cutaneous melanoma [71]. Due to the low number of starting cell line samples and the limited concordance of the identified mutational profiles, we focused only on the above* BRAF*,* MSH3*, and* ALPK2* mutations for the execution of the “Translational” workflow on a set of clinical genomics data for about 470 melanoma samples from the TCGA database [52]. Hierarchical clustering evaluation of the melanoma samples showed a group of predicted “Sensitive” melanoma samples with mutated* BRAF* and WT* ALPK2*; only a small fraction of melanoma samples showed mutated* MSH3* without a clear clustering pattern (Figure 4(c)). In the majority of melanoma samples,* BRAF* and* ALPK2* molecular alterations appeared to be mutually exclusive; however* ALPK2* has been reported among genes that are mutated in significantly higher proportion of melanoma cell lines than in melanoma tumors [72]. We repeated the melanoma clinical analysis using only* BRAF* for sample classification: the resulting “Sensitive” melanoma group was enriched in primary tumor samples derived from “trunk” rather than other excision sites and from patients with an average lower age as compared to predicted “Resistant” patients (54.3 versus 60.3, Figure 4(d)), in agreement with reported literature [73, 74].

**Figure 5:**
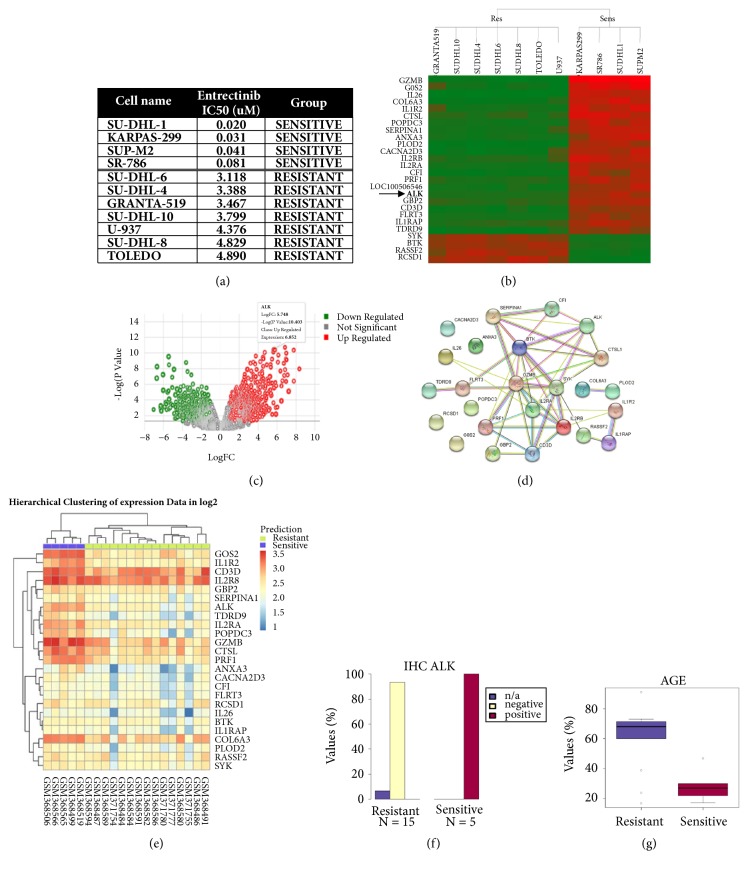
‘‘**Translational” analysis of entrectinib sensitivity in lymphoma cell lines and clinical samples. **Results of PATRI “Translational” analysis workflow performed on a panel of lymphoma cell lines sensitive or resistant to entrectinib treatment and on two panels of 20 and 130 lymphoma clinical samples (GSE14879 [25] and GSE19069 [26], respectively). (a) List of lymphoma cell lines with respective entrectinib IC50 values. A threshold of 1*μ*M was chosen to define “Sensitive” and “Resistant” cell lines. (b) Heatmap dendrogram of 26 markers differentiating entrectinib sensitive versus resistant lymphoma cell lines, obtained by Limma gene expression analysis (p value<10^−7^, logFC>|5|). Significantly high or low expressed genes are highlighted in red and green, respectively. (c) Volcano plot visualizing significance and magnitude of gene expression differences in sensitive versus resistant conditions, with pop-up indicating ALK expression level. Significantly high or low expressed genes (p value<0.05, logFC>|1|) are highlighted in red and green, respectively. (d) Results from STRING analysis [27] showing the protein-protein interaction network connecting the identified genes (STRING interaction score: 0.150). (e) Heatmap representing hierarchical cluster analysis via random forest categorization of the predicted “Sensitive” or “Resistant” GSE14879 lymphoma samples, based on the selected genes (sample IDs are represented on the lower part of the graph). (f-g) Top ranking of significant clinical features associated with GSE14879 lymphoma samples, classified as potentially “Sensitive” or “Resistant”. (f) Histogram representing distribution of clinically assessed ALK immunohistochemistry (IHC) positivity (reported in [25]) in the predicted “Sensitive” and “Resistant” sample groups. (g) Box plot representing the distribution of the reported age [25] in predicted “Sensitive” (avg. 21.6) and “Resistant” (avg. 61.3) lymphoma sample groups.

We then considered a panel of lymphoma cell lines formerly tested in our labs for sensitivity to entrectinib ([37] and Figure 5(a)), a new TRKs/ALK/ROS1 inhibitor currently showing great promise in phase I/II clinical trials on tumors driven by rearrangements of one of these kinases [37, 75]. The panel included 4 anaplastic large cell lymphoma (ALCL) cell lines, all harboring the nucleophosmin* NPM-ALK* rearrangement [76], and all extremely sensitive to treatment with entrectinib. In the PATRI gene expression analysis of the 4 sensitive versus 7 resistant lymphoma cell lines with Limma, ALK resulted as the most statistically significant overexpressed kinase (Figures 5(b) and 5(c)). The most differentially expressed genes (p value<10^−7^, logFC> |5|, Figures 5(b) and 5(c)) found in the entrectinib sensitive lymphoma cell lines were subjected to STRING analysis [27] and resulted to be significantly networked with ALK (Figure 5(d)) and found to be transcriptionally regulated in ALK activated pathways [[26, 77–80] and reviewed in [81, 82]]. “Translational” analysis of these markers in two distinct gene expression clinical non-Hodgkin's lymphoma datasets (GSE14879, 20 samples [25], and GSE19069, 130 samples [26]) correctly predicted and clustered the 5 ALK-positive ALCL samples from GSE14879 (Figure 5(e)), with immunohistochemistry positivity features for ALK (Figure 5(f)) and PRF1 (not shown) and younger age (Figure 5(g)) ranking with highest statistical significance. Interestingly, a comparable result was achieved with a 22-gene list obtained with ANOVA gene expression analysis (p value<10^−5^, logFC> |4.5|) not containing ALK, though with a less defined heatmap “Sensitive” versus “Resistant” cluster pattern (not shown). In GSE14879, predicted “Sensitive” samples included most of the ALK-positive ALCL samples and also included 5 Peripheral T-Cell lymphoma, unspecified (PTCL-NOS) samples, however displaying again ALK-positive diagnosis and lower age among the top ranking significant clinical associated parameters (not shown). Mutational analysis of the entrectinib-treated lymphoma cell line panel did not provide significant results, while copy number analysis with ANOVA revealed only two markers with significant microalterations, namely, TCR gamma alternate reading frame protein (*TARP*) loss and ADAM metallopeptidase domain 6 pseudogene (*ADAM6*) gain in entrectinib sensitive, ALK-positive cell lines. The significance of these two markers could not be explored using the PATRI translational workflow, since only gene expression data were available for the same samples in the two lymphoma clinical datasets.

**Figure 6:**
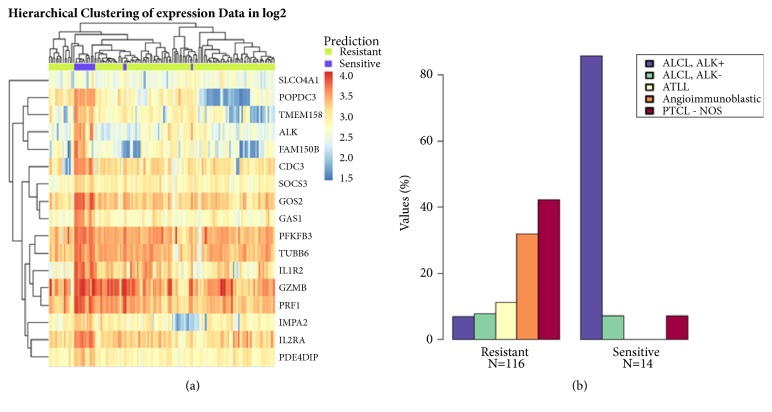
**Simulation of **‘‘**Clinical” analysis workflow based on presumed ALK inhibitor sensitivity in lymphoma clinical samples. **Results of PATRI “Clinical” analysis workflow, simulated using two panels of lymphoma clinical samples. The 5 ALK-positive ALCL samples in the GSE14879 dataset (sample IDs: GSM368499, GSM368506, GSM368519, GSM368565, and GSM368566) were presumed to be ALK inhibitor responders and set as “Sensitive” samples only for validation purposes, using the PATRI available gene expression analysis algorithms. (a) Heatmap representing hierarchical cluster analysis via random forest categorization of the predicted “Sensitive” or “Resistant” 130 samples in the lymphoma GSE19069 dataset (sample IDs could not be represented on the lower part of the graph), starting from a filtered 17-gene expression biomarker list obtained by Limma analysis of the GSE14879 dataset (p value<10^−10^, logFC>|1|). (b) Histogram representing lymphoma diagnosis distribution for the 14 predicted “Sensitive” and the 116 “Resistant” lymphoma samples from the GSE19069 dataset (ALCL, ALK+: anaplastic large cell lymphoma ALK-positive; ALCL, ALK-: anaplastic large cell lymphoma ALK-negative; PTCL-NOS: peripheral T-cell lymphoma, unspecified; ATLL: adult T-cell leukemia/lymphoma; Angioimmunoblastic: angioimmunoblastic T-cell lymphoma).

The same lymphoma clinical datasets were also used to simulate a “Clinical” workflow analysis, presuming the 5 ALK-positive ALCL samples in the GSE14879 dataset as ALK inhibitor treatment “responder” patient samples for validation purposes. PATRI biomarker analysis was executed with Limma and a filtered 17-gene list (p value<10^−10^, logFC> |1|) was used for exploration and sensitivity prediction in the lymphoma GSE19069 dataset (Figure 6(a)), resulting in the prediction of 14 “Sensitive” lymphoma samples mostly containing ALK-positive ALCL samples, with top ranking clinical annotations for ALK-positive ALCL diagnosis (Figure 6(b)) and younger age (not shown). The provided results illustrate the feasibility of the PATRI “Clinical” analysis workflow for the quick evaluation and the comparison of “training versus test” dataset biomarker analysis correlations for all available clinical datasets with consistent phenotypic annotations.

## 4. Conclusion

In this work, we describe PATRI, a freely available standalone tool conceived as a biomarker data analysis “starter kit” for basic users, enabling flexible storage, analysis, and complementation of preclinical and clinical baseline genomics data for correlation with treatment sensitivity, allowing the exploration of potential predictive therapeutical biomarkers.

The current version of the tool design, along with widely accepted algorithms and graphical representations, introduces a “Translational” workflow, supporting rapid clinical evaluation of putative preclinical therapeutic response biomarkers in annotated clinical genomics datasets, based on random forest categorization in parallel with phenotypic significance analysis. The same workflow can also be applied across distinct clinical datasets (“Clinical” workflow).

We have proposed examples of use of PATRI with in-house sensitivity data from representative targeted drugs with well-established mutated or overexpressed biomarkers; however, PATRI might also be applied to support the identification of new relevant biomarkers and indicators of sensitivity in other types of treatments, such as RNA interference or CRISPR/Cas9 screenings, as well as for the evaluation of their frequency and relevance in the clinics.

The PATRI structure can be integrated with further analysis methods, available as R packages, making the tool a suitable platform for future implementation of innovative analysis approaches in biomarker discovery, such as the integration of novel prediction algorithms [83–86], possibly supporting also the identification of synergistic combinations [87], or the handling of confounding factors in preclinical cancer model variability [88]. One easy adaptation might be, for example, the emerging promising field of the identification of splicing gene isoforms or transcriptomics biomarkers as novel predictors of drug response [89].

## Data Availability

All the data used in the paper are publicly available and have been referenced accordingly. The produced analysis results are only for validation purposes; if requested we can provide them as stated in the present Data Statement.

## Supplementary Materials

Supplementary Materials The Supplementary File to “PATRI, a Genomics Data Integration Tool for Biomarker Discovery”, by Ukmar, G. et al., contains the following 3 sections: (1) Supplementary Figures 1-7 (Supplementary Figure 1: PATRI database scheme; Supplementary Figure 2: PATRI graphical user interface: Analysis windows; Supplementary Figure 3: PATRI graphical user interface: “New Analysis” algorithm selection windows; Supplementary Figure 4: PATRI graphical user interface: “Selected Analysis” results window; Supplementary Figure 5: PATRI graphical user interface: Graphing options; Supplementary Figure 6: PATRI graphical user interface: Clinical Samples windows; Supplementary Figure 7: PATRI graphical user interface: Clinical Samples Analysis results and graphing options); (2) PATRI Installation and Configuration Guide, Version 1.0; (3) PATRI Platform User's Guide, Version 1.0.
