# Supplementary material for: PATRI, a Genomics Data Integration Tool for Biomarker Discovery
Source: Biomed Res Int. 2018 Jun 28;2018:2012078. doi: 10.1155/2018/2012078 (PMC6051285; doi:10.1155/2018/2012078)
Supplement: Supplementary Materials — The Supplementary File to “PATRI, a Genomics Data Integration Tool for Biomarker Discovery”, by Ukmar, G. et al., contains the following 3 sections: (1) Supplementary Figures 1-7 (Supplementary Figure 1: PATRI database scheme; Supplementary Figure 2: PATRI graphical user interface: Analysis windows; Supplementary Figure 3: PATRI graphical user interface: “New Analysis” algorithm selection windows; Supplementary Figure 4: PATRI graphical user interface: “Selected Analysis” results window; Supplementary Figure 5: PATRI graphical user interface: Graphing options; Supplementary Figure 6: PATRI graphical user interface: Clinical Samples windows; Supplementary Figure 7: PATRI graphical user interface: Clinical Samples Analysis results and graphing options); (2) PATRI Installation and Configuration Guide, Version 1.0; (3) PATRI Platform User's Guide, Version 1.0. [file 2012078.f1.docx]

**SUPPLEMENTARY MATERIALS**

PATRI, a genomics data integration tool for biomarker discovery

Ukmar G^1†^, Melloni GEM^2,7†^, Raddrizzani L^1†^, Rossi P^3^, Di Bella S^1^, Pirchio MR^3^, Vescovi M^5^ , Leone A^1^, Callari M^4,6^, Cesarini M^2^ , Somaschini A^1^, Della Vedova G^2^, Daidone MG^4^, Pettenella M^5^, Isacchi A^1^, Bosotti R^1^*

† these authors equally contributed to the work

1 NMS Oncology, Nerviano Medical Sciences Srl, Nerviano (MI), Italy
2 University of Milano Bicocca, Milano, Italy

3 Icona Srl, Cinisello Balsamo (MI), Italy

4 Fondazione IRCCS Istituto Nazionale dei Tumori, Milano, Italy

5 Parametric Design Biotech, Gessate (MI), Italy

6 Current address: Cancer Research UK Cambridge Institute, University of Cambridge, UK

7 Current address: Harvard Medical School, Boston MA, USA

***Corresponding Author**

Roberta Bosotti

Nerviano Medical Sciences srl

Viale Pasteur 10, 20014 Nerviano (MI), Italy

[roberta.bosotti@nervianoms.com](mailto:roberta.bosotti@nervianoms.com) Supplementary Figure 1: **PATRI database scheme**

**
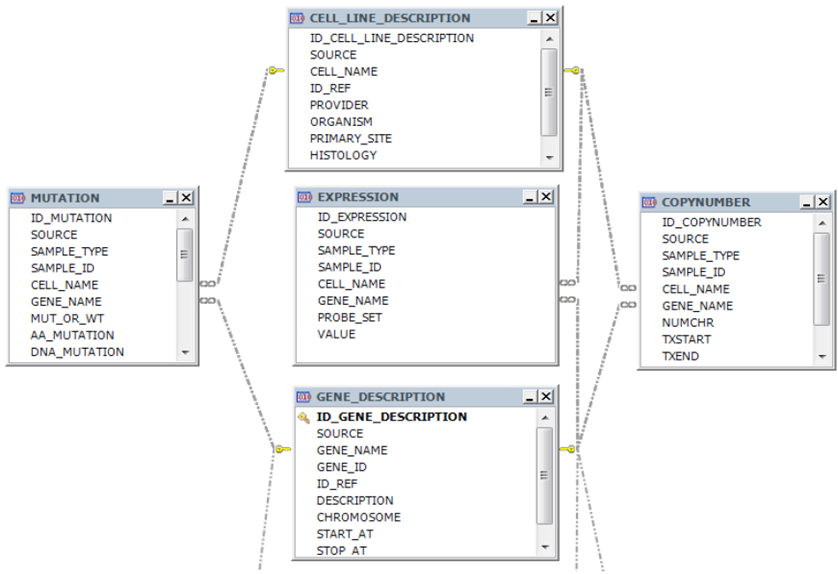

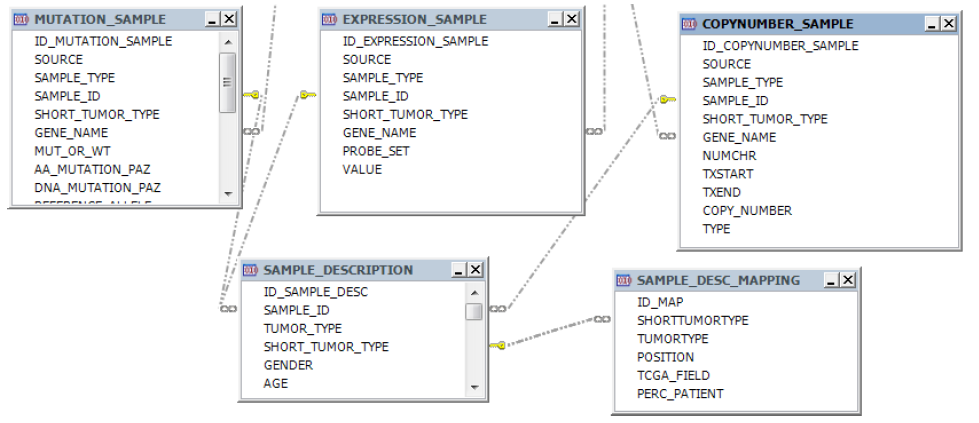
**Legend to Supplementary Figure 1: **PATRI database scheme**

Table structures composing the PATRI database are represented, with connecting cross links. Green and red boxes identify tables dedicated to cell lines and clinical samples, respectively. For each sample type, a description table (“cell_line_description” and “sample_description”) is provided for upload of sample annotation characteristics, each with fixed fields, plus a number of customizable fields available for clinical samples, as obtained for example from the patients’ Case Report Forms (CRFs), including 3 fields used to generate Kaplan-Meier (KM) survival curves, i.e. days to death, days to last follow up and vital status). Three dedicated tables for cell lines and three tables for clinical samples, respectively, are defined for molecular data (mutation, expression and copy number). All data are cross-referenced to a table called “gene_description”, containing gene information that can be populated with gene information obtained from NCBI RefSeq [RefSeq: NCBI Reference Sequence Database, https://www.ncbi.nlm.nih.gov/refseq/.] or other preferred sources. Instructions on how to format and import data are provided in the PATRI Platform User’s Guide and PATRI Platform Installation and Configuration Guide downloadable documents (see also Supplementary File).

Supplementary Figure 2: **PATRI graphical user interface: Analysis windows**


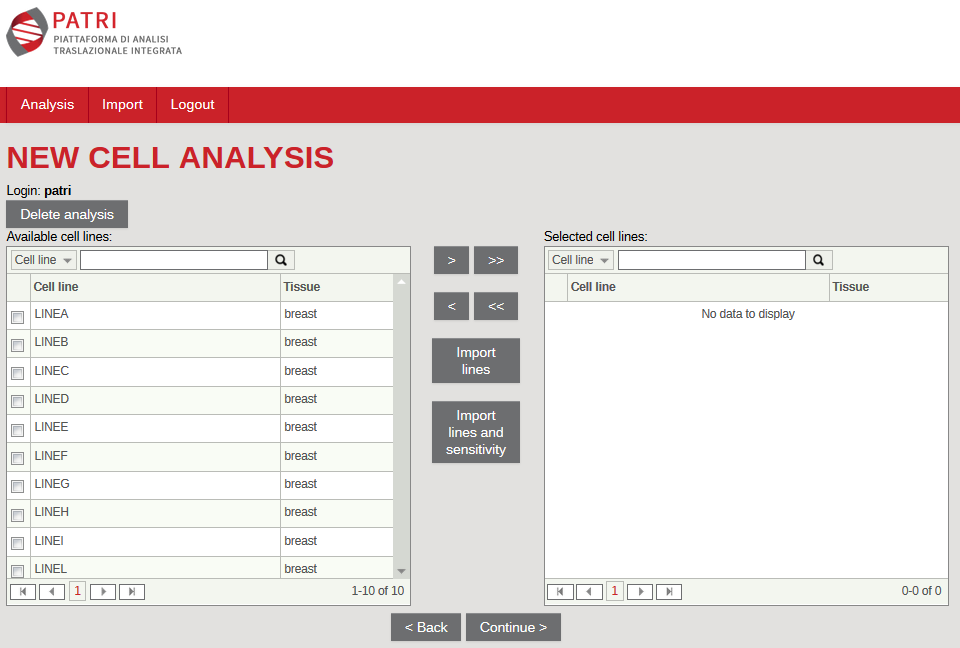


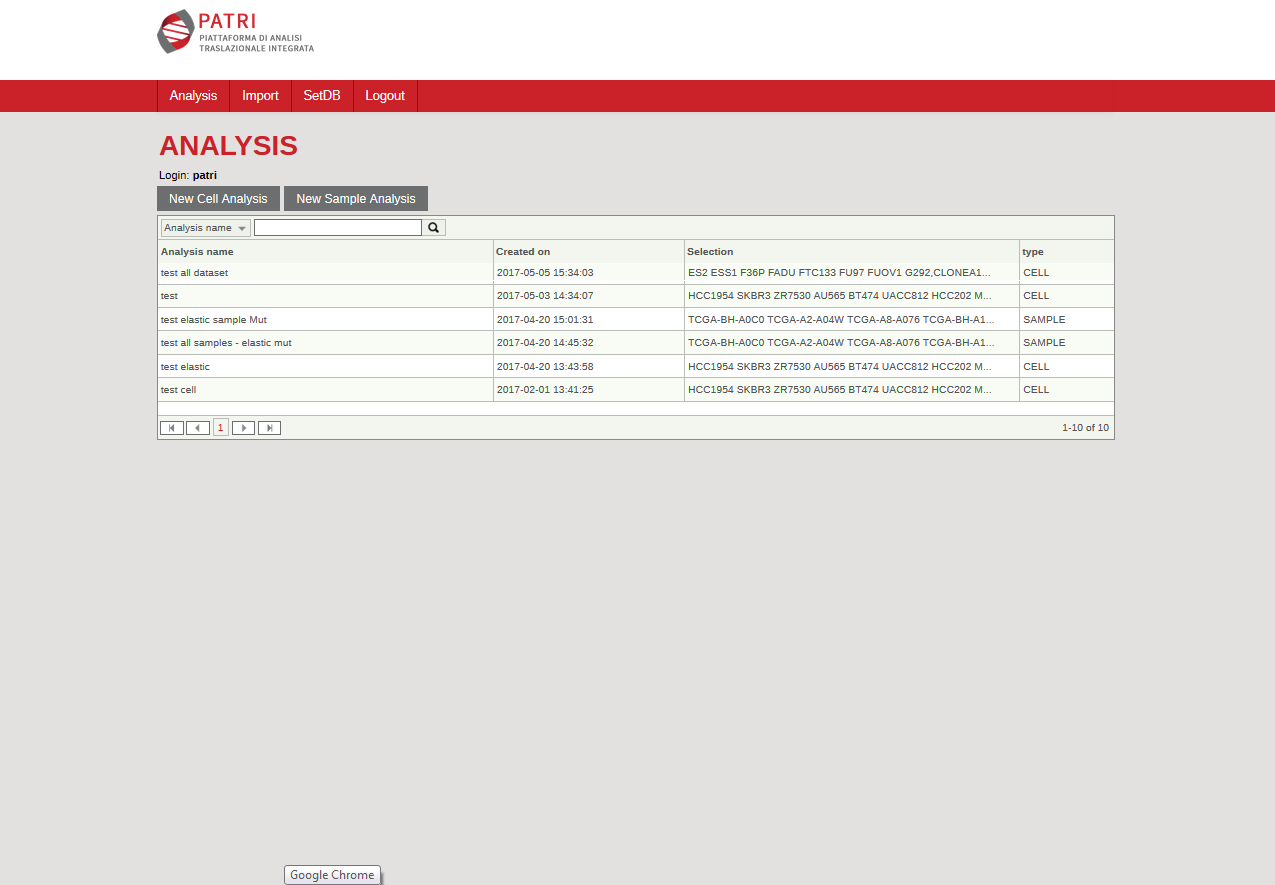

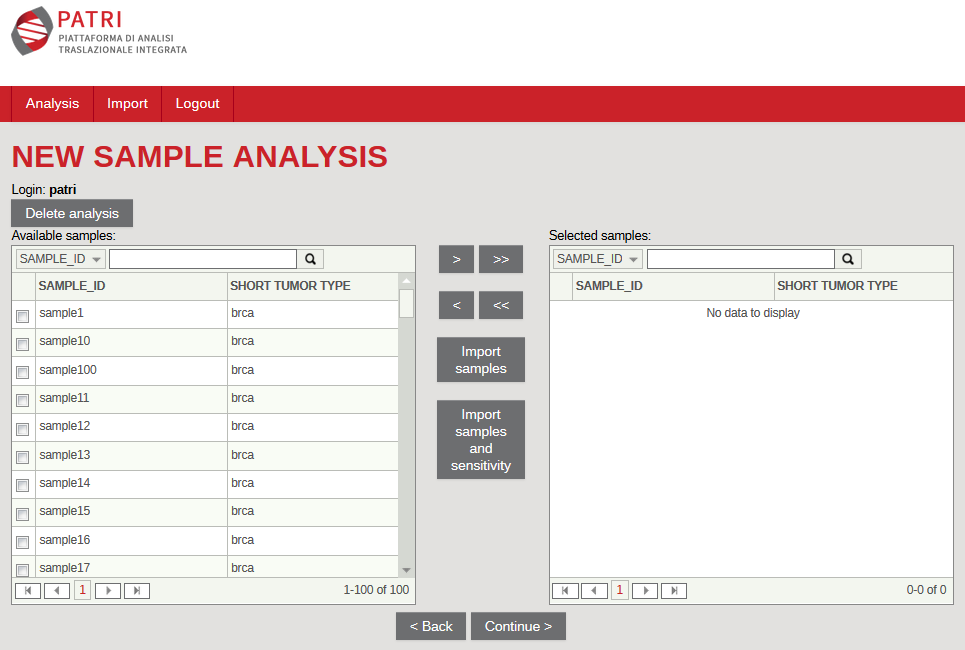


Legend to Supplementary Figure 2: **PATRI graphical user interface: Analysis windows**

Screenshots of ‘Analysis’ windows. All PATRI functionalities can be intuitively accessed by horizontal menu. The main Analysis window lists all archival analyses, which can be opened by mouse click. Start buttons are provided to initiate new ‘Cell Line’ or clinical ‘Sample’ analyses. Sensitive and resistant cell lines or samples can be selected directly from the displayed list and labeled as ‘sensitive’ or ‘resistant’ on the basis of their response to a specific drug treatment or browsed from a user-defined .txt file with cell line and respective label in each row. The system automatically retrieves and associates the genomics data uploaded in PATRI tables to the list of provided samples.

Supplementary Figure 3: **PATRI graphical user interface: ‘New Analysis’ algorithm selection windows**


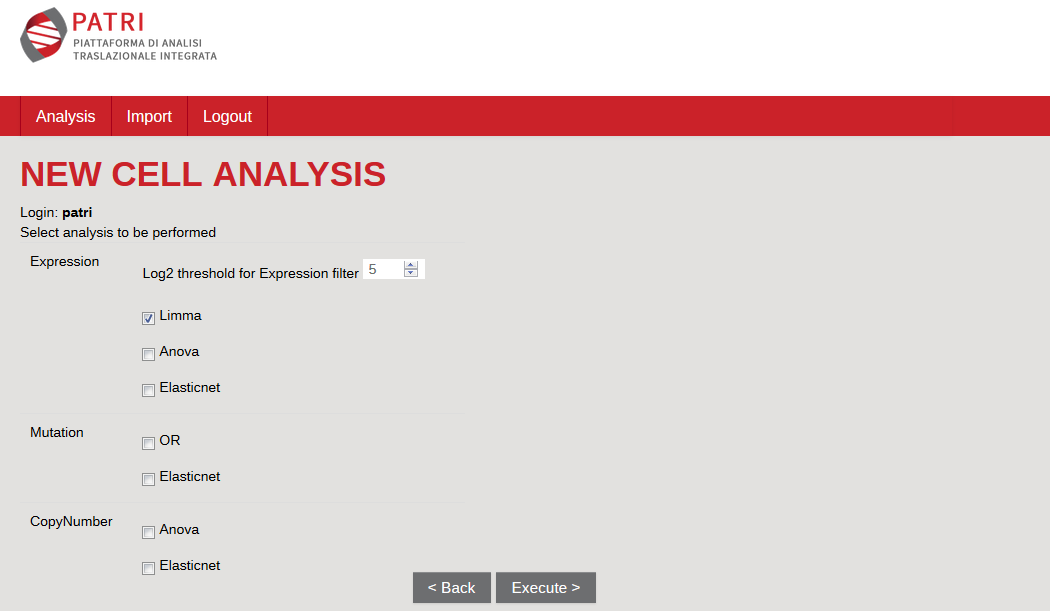

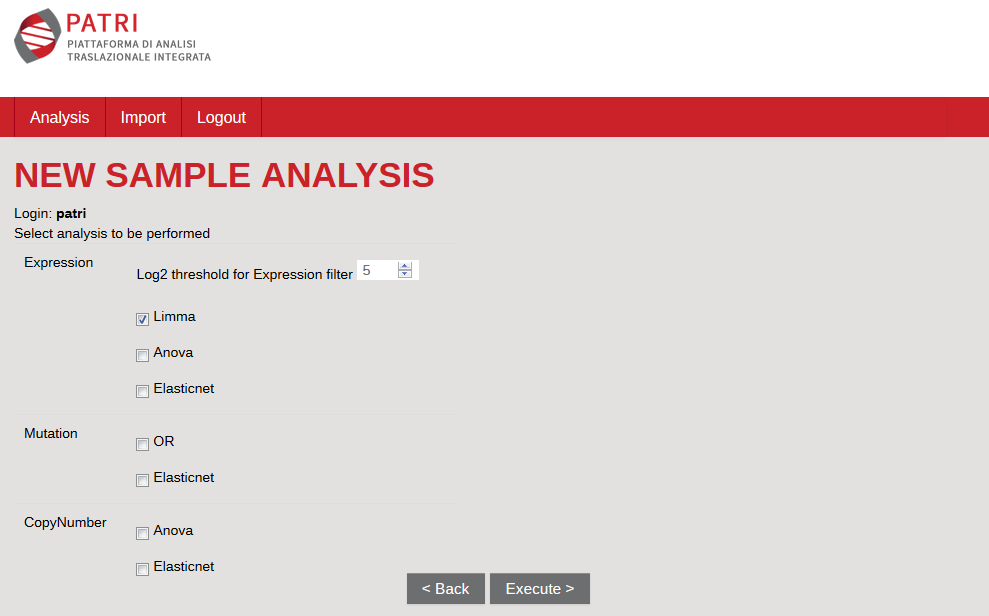


Legend to Supplementary Figure 3: **PATRI graphical user interface: ‘New Analysis’ algorithm selection windows**

Screenshots of ‘New Cell Analysis’ and ‘New Sample Analysis’ panes, allowing selection and simultaneous execution of the built-in statistical algorithms for genomics data analysis.

Supplementary Figure 4: **PATRI graphical user interface: ‘Selected Analysis’ results window**


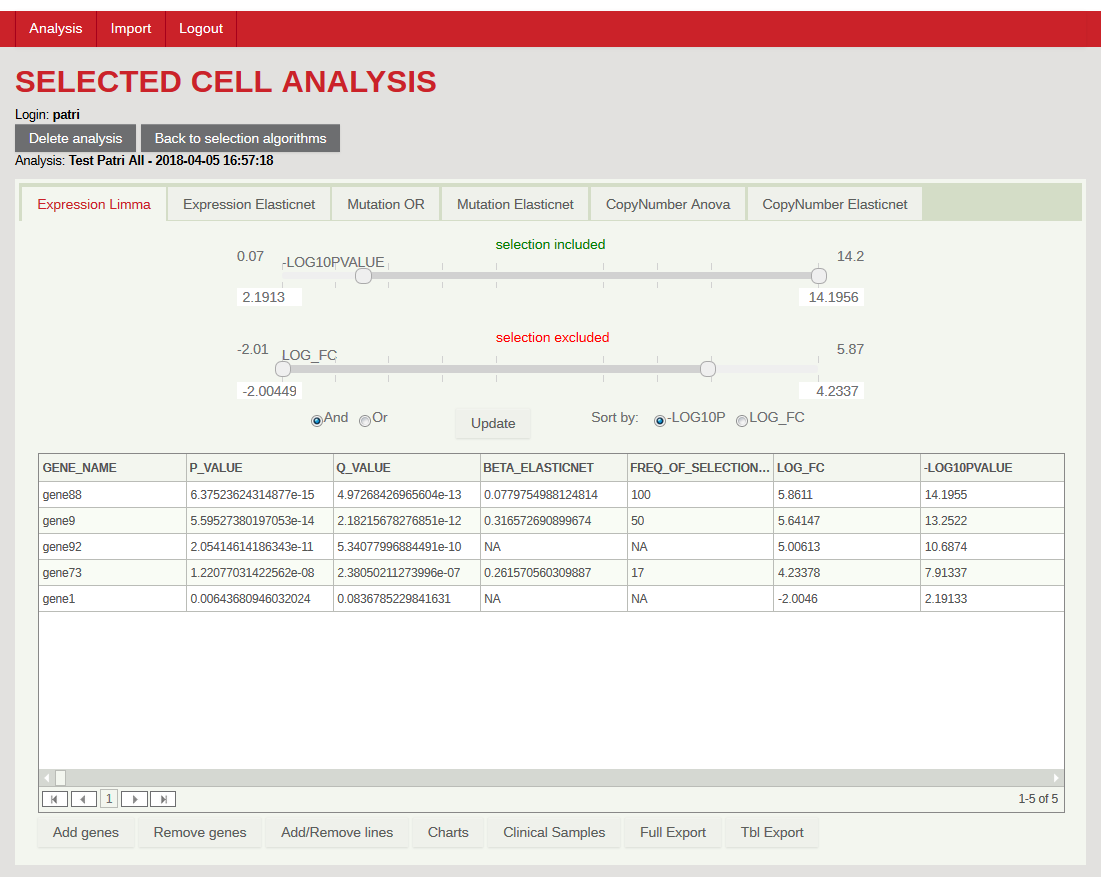


Legend to Supplementary Figure 4: **PATRI graphical user interface: ‘Selected Analysis’ results window**

Screenshot of ‘Selected Cell Analysis’ (similar to ‘Selected Sample Analysis’) pane, where results of the selected analyses are displayed for each gene in the dataset, with sorting buttons and filtering slide bars; a pop-up option is available to view the corresponding gene information upon mouse click. Additional buttons provide access to charting functions and to searching of additional genes or samples not included in the filtered results. The list of identified genes can be exported as a text file by clicking on Export buttons from the Analysis results or Charts pages, together with gene expression values, copy number values or mutational status for each gene and sample. An additional ‘Clinical Sample’ button is active to proceed to exploration of the status of the selected candidate biomarker gene lists in the available clinical sample genomics data.

Supplementary Figure 5: **PATRI graphical user interface: Graphing options**


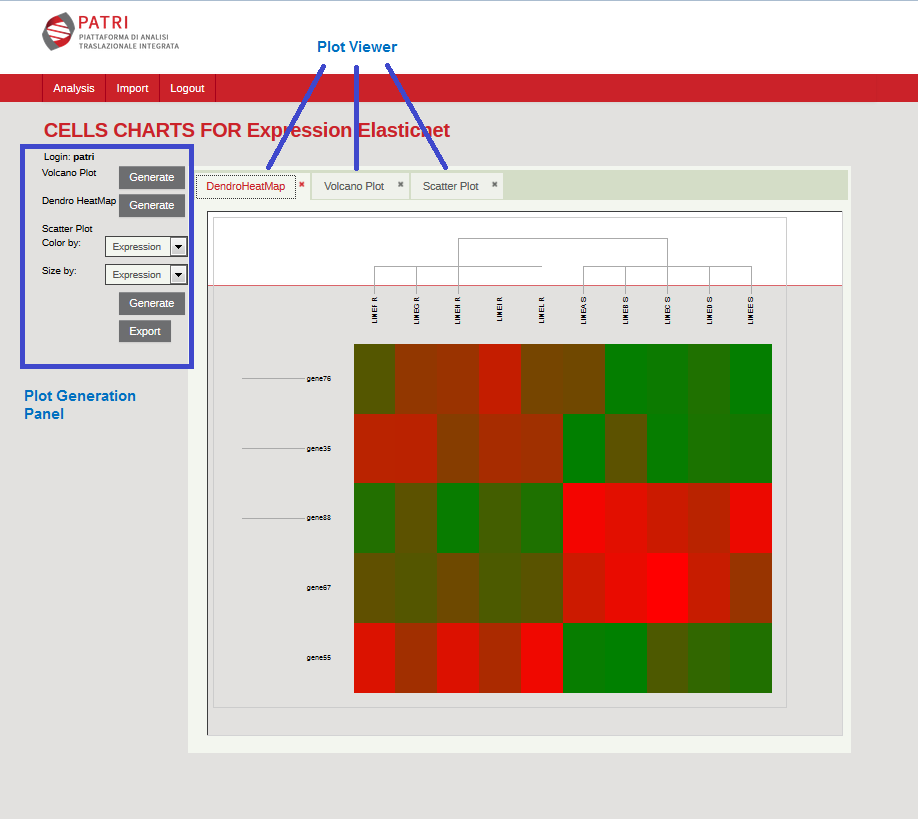

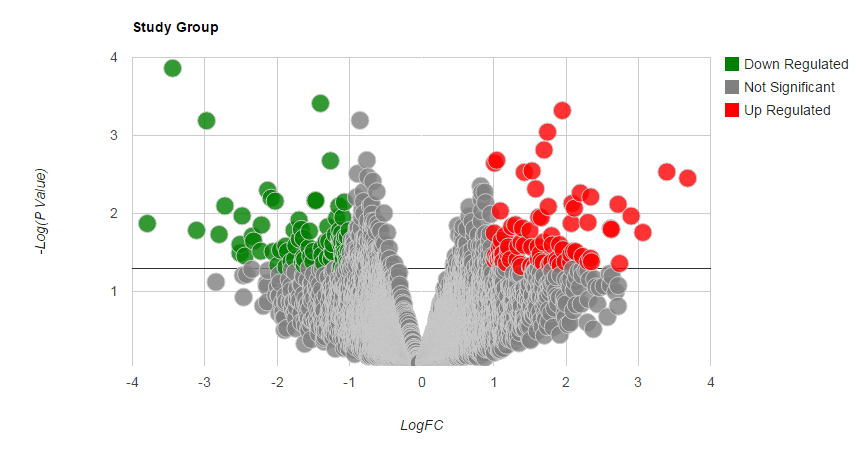

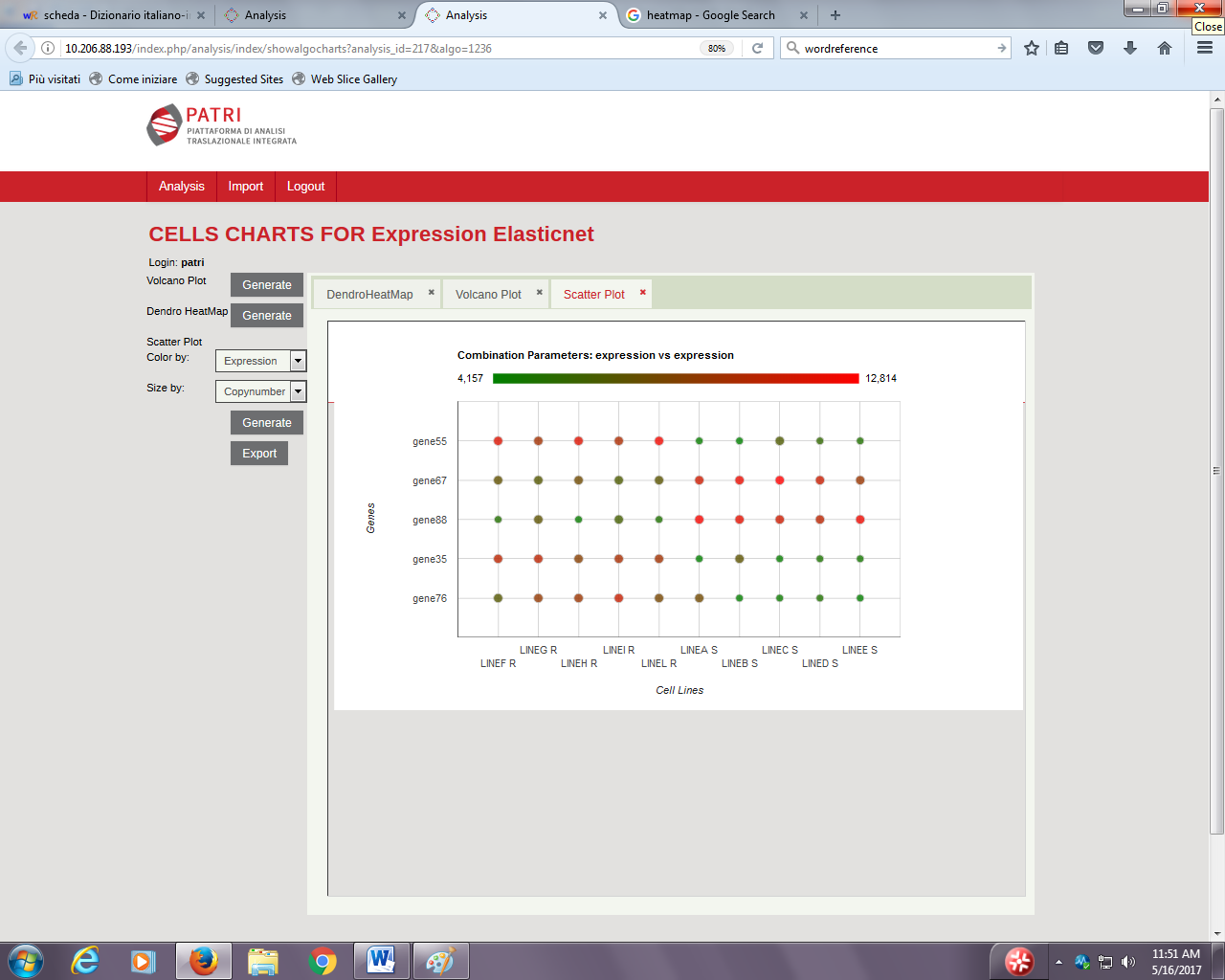


Legend to Supplementary Figure 5: **PATRI graphical user interface: Graphing options**

Examples of graphing options available in PATRI, in particular: (top) interactive Volcano plot, (middle) heatmap, and (bottom) scatter plot, where results from different analysis types can be combined by dot size and color shades. Mouseover and zooming options are available to facilitate data inspection.

Supplementary Figure 6: **PATRI graphical user interface: Clinical Samples pane**


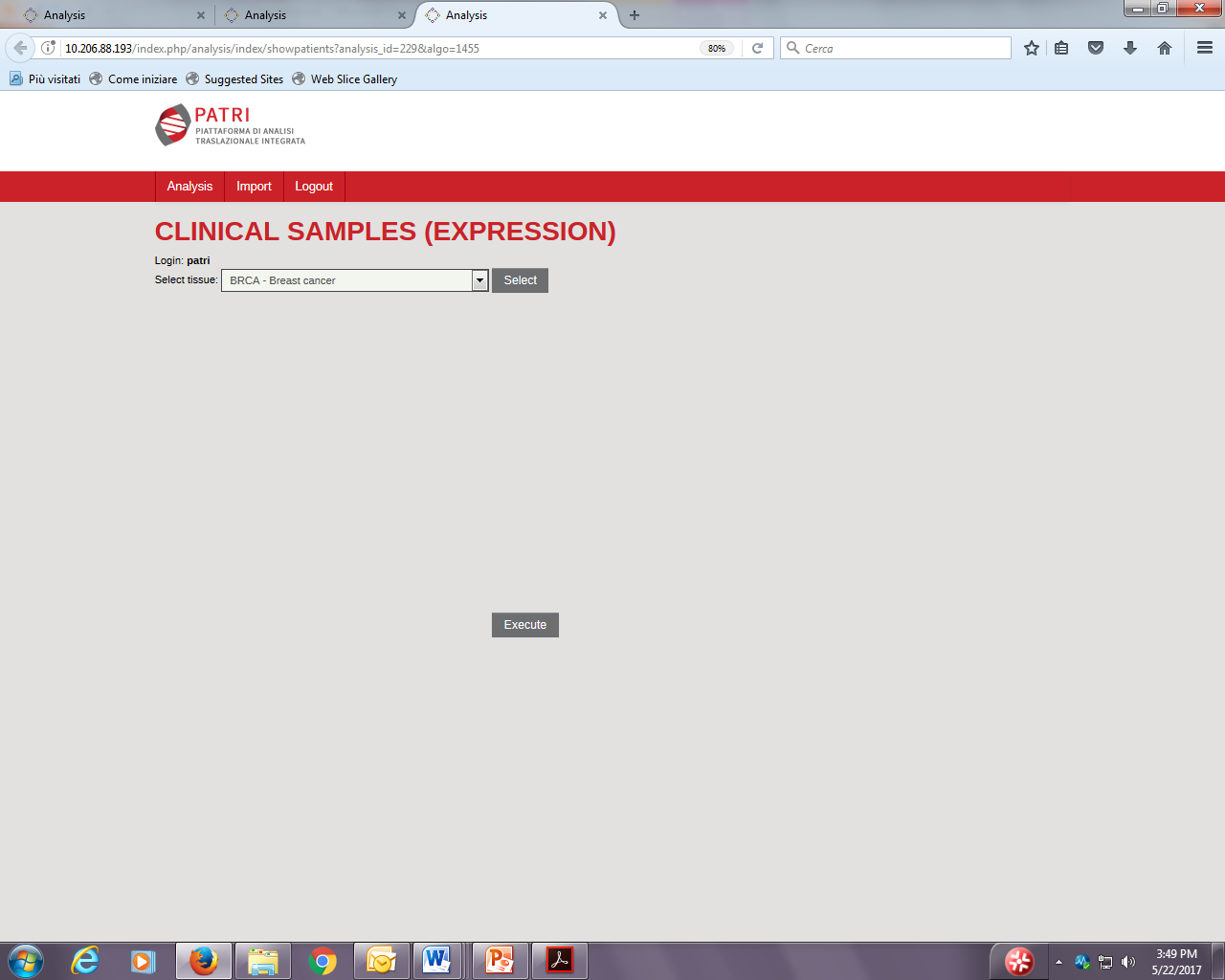


Legend to Supplementary Figure 6: **PATRI graphical user interface: Clinical Samples pane**

Screenshot of Clinical Samples pane, where results of cell line candidate biomarker evaluation can be explored in the available collections of clinical information for the presence of clinical subpopulations that might be defined based on the status of the identified sensitivity biomarkers. By clicking on the ‘Select’ button, details on the dataset content will be extracted and displayed.

Supplementary Figure 7: **PATRI graphical user interface: Clinical Samples Analysis results and graphing options**


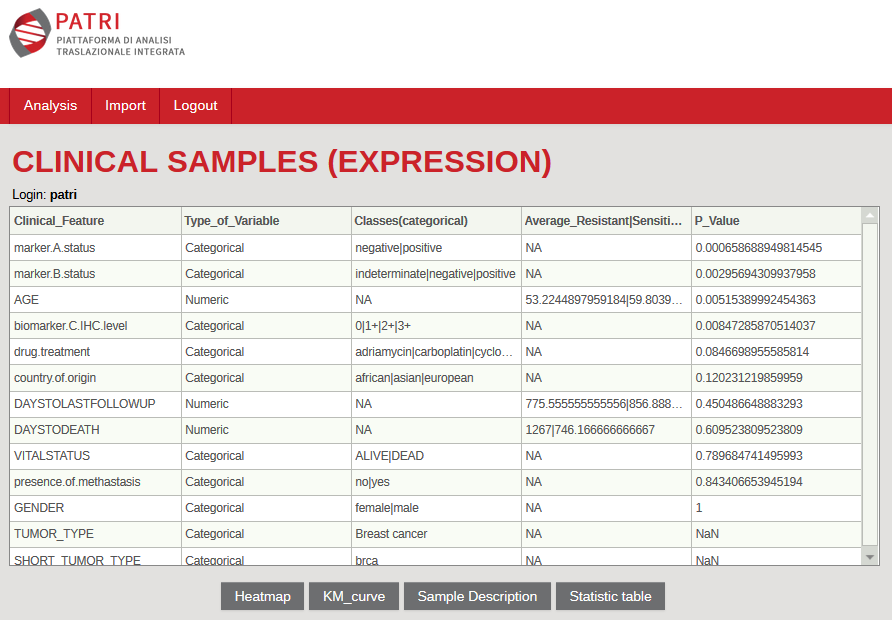


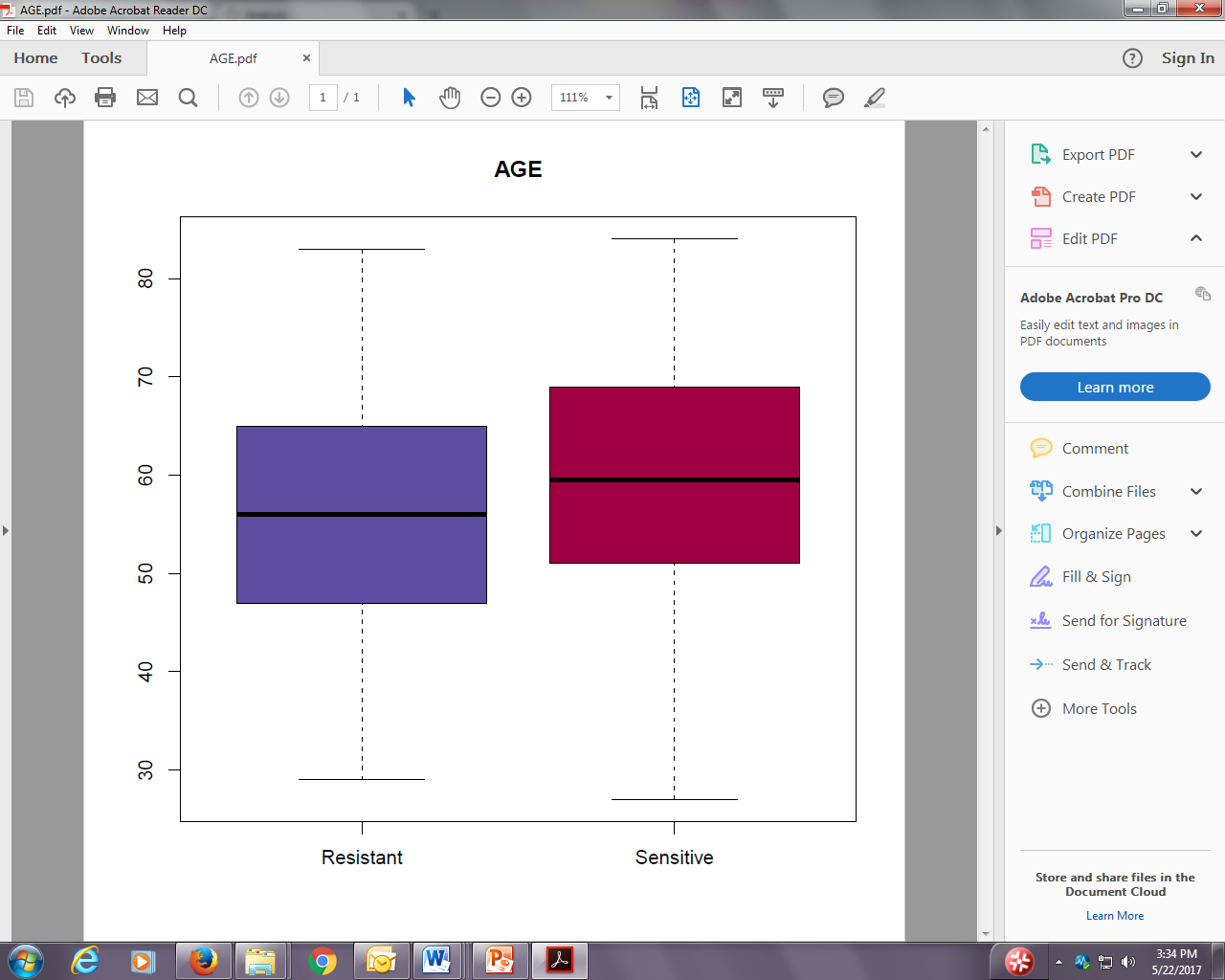

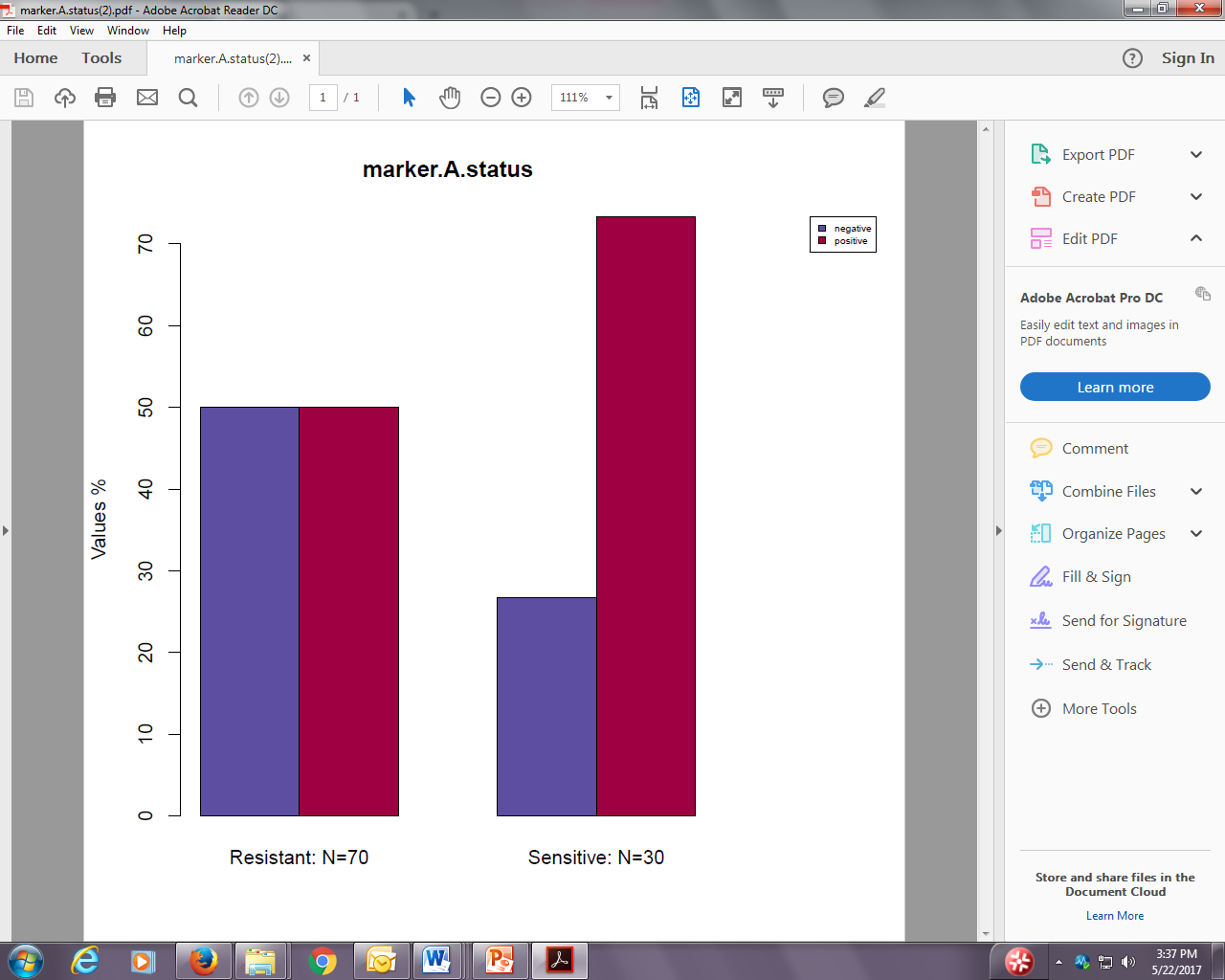


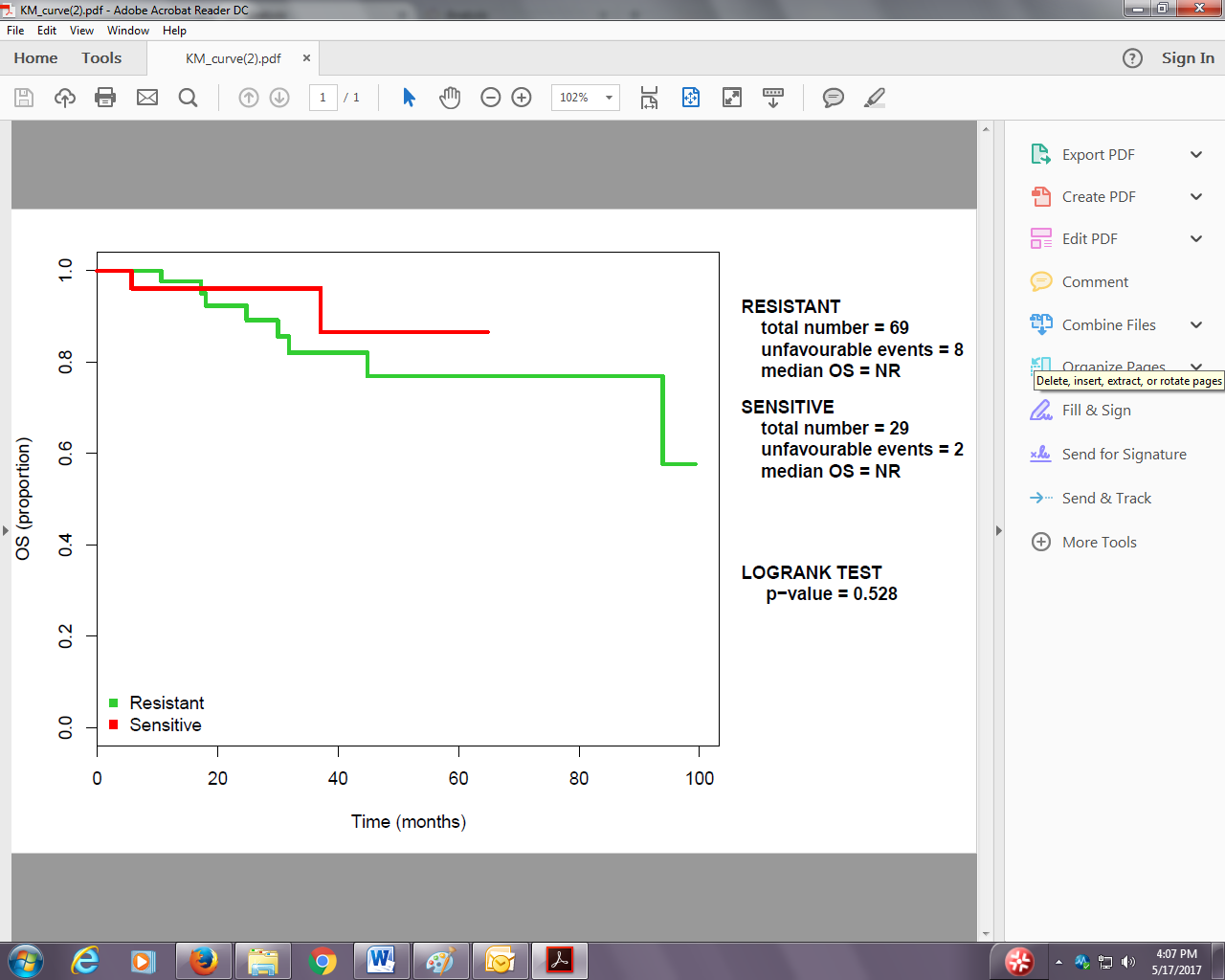

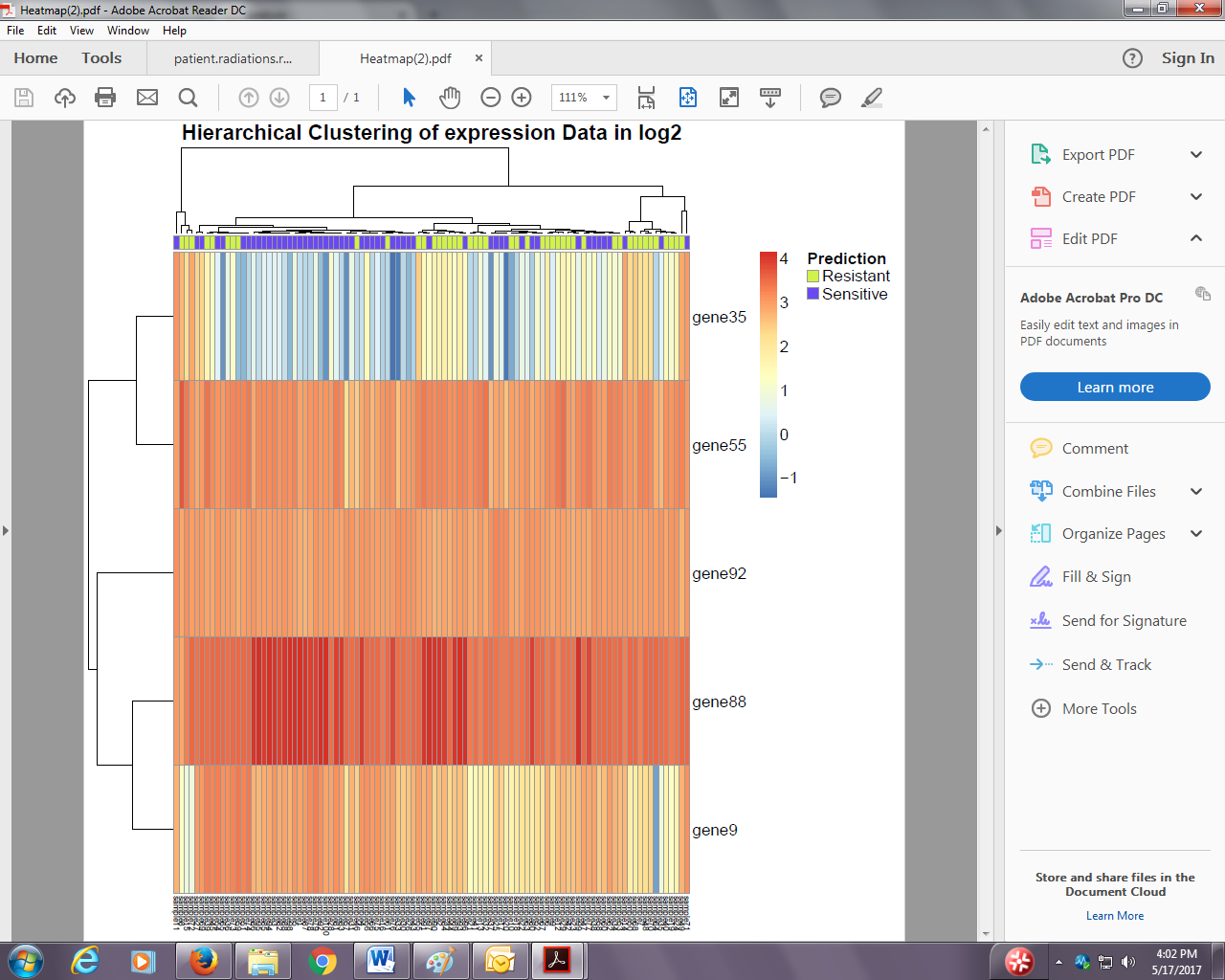


Legend to Supplementary Figure 7: **PATRI graphical user interface: Clinical Samples Analysis results and graphing options**

Top, screenshot of the Clinical Samples analysis results, displaying a list of statistically significant clinical parameters that can be retrieved and displayed for graphical evaluation. Bottom: examples of boxplots, bar graphs, Kaplan-Meier curves and prediction heatmaps, generated by clicking on the desired items within the table or on the lower buttons.

Suppl. File 1

PATRI – Installation and Configuration Guide

Version 1.0

DISCLAIMER NOTICE:

THE PATRI SOFTWARE IS PROVIDED "AS IS", WITHOUT WARRANTY OF ANY KIND, EXPRESS OR IMPLIED, INCLUDING BUT NOT LIMITED TO THE WARRANTIES OF MERCHANTABILITY, FITNESS FOR A PARTICULAR PURPOSE AND NONINFRINGEMENT. IN NO EVENT SHALL THE AUTHORS BE LIABLE FOR ANY CLAIM, DAMAGES OR OTHER LIABILITY, WHETHER IN AN ACTION OF CONTRACT, TORT OR OTHERWISE, ARISING FROM, OUT OF OR IN CONNECTION WITH THE SOFTWARE OR THE USE OR OTHER DEALINGS IN THE SOFTWARE.

Table of Contents

[Introduction 16](#_Toc514849208)

[Setup and installation 17](#_Toc514849209)

[System requirements 17](#_Toc514849210)

[Software requirements 18](#_Toc514849211)

[Virtual Machine Installation 18](#_Toc514849212)

[Launch the Virtual machine 23](#_Toc514849213)

[Switch off virtual machine 25](#_Toc514849214)

[New User creation 26](#_Toc514849215)

[Introduction 30](#_Toc514849216)

[Home page 31](#_Toc514849217)

[Analysis 32](#_Toc514849218)

[New Cell line Analysis: Resistant / Sensitive cell line labels 36](#_Toc514849219)

[New Cell line Analysis: Statistical Algorithm Selection 38](#_Toc514849220)

[Selected Cell Analysis 40](#_Toc514849221)

[Charts 45](#_Toc514849222)

[Heatmap 46](#_Toc514849223)

[Volcano Plot 46](#_Toc514849224)

[Scatter Plot 48](#_Toc514849225)

[*Fig. 19 Scatter plot* 48](#_Toc514849226)

[Clinical Samples 49](#_Toc514849227)

[New Sample Analysis: label of the samples 57](#_Toc514849228)

[New Sample Analysis: Algorithm choice 58](#_Toc514849229)

[Selected Sample Analysis 59](#_Toc514849230)

[Data visualization by Charts 60](#_Toc514849231)

[Import 60](#_Toc514849232)

[Import Expression 61](#_Toc514849233)

[Import Mutation 63](#_Toc514849234)

[Import Copy number 66](#_Toc514849235)

[Import Cell Line Description 67](#_Toc514849236)

[Import Gene Description 69](#_Toc514849237)

[Import Clinical Sample Description 70](#_Toc514849238)

# Introduction

This document describes PATRI (Platform for the Analysis of TRanslational Integrated data) installation and configuration procedures developed by Parametric Design, Nerviano Medical Science, Istituto Nazionale dei Tumori, Università di Milano Bicocca and Icona.

PATRI is a stand-alone tool accessible through a user-friendly graphical interface for the analysis of user-provided genomics data associated to information on sample characteristics. PATRI resides on a MariaDB database and uses some Open Source R libraries for the analysis. It can be applied to the identification of molecular markers discriminating any two conditions (e.g. sensitivity or resistance to a drug), leading to the detection of potentially predictive biomarkers.

PATRI is based on client-server architecture with a software component dedicated to the application use (client part) and a software component designed to manage the queries and generate the output (server part).

This document explains the following tasks:

- Installation requirements
- System requirements
- Platform installation
- Platform configuration
- Virtual machine launch
- Virtual machine switch off
- User ID set up

# Setup and installation

PATRI is based on client-server architecture. It is a web-based application: the Client component is a Web Browser, while the server was implemented as a Virtual Machine with UNIX installation.

In the following pages PC/server software and hardware requirements for PATRI installation are reported together with instructions on how to install and configure the virtual Machine and start/end platform execution.

Note: For information on how to use PATRI, please refer to *PATRI – Platform User’s Guide*.

# System requirements

|  | **Windows requirements** | **Macrequirements** | **Linuxrequirements** |
| --- | --- | --- | --- |
| **Operating system** | - Windows XP, Service Pack 2 or higher - Windows Vista - Windows 7 - Windows 8 | Mac OS X 10.6 or higher | Ubuntu 12.04 or higher  Debian 7 or higher OpenSuSE 12.2 or higher  Fedora Linux 17 |
| **Processor** | Intel Pentium 4 or higher | Intel | Intel Pentium 4 or higher |
| **Freedisk space** | 100 GB recommended. 80 GB required. | |  |
| **RAM** | 8 GB recommended. 4 GB required. | |  |

## Software requirements

The following pre-installed programs are required to run PATRI:

- Web Browser:

PATRI is compatible with:

o Google Chrome 41 or higher (recommended)

o Mozilla Firefox 36 or higher

o Internet Explorer 11

- Oracle VM Virtual Box 4.3.8 or higher

## Virtual Machine Installation

To install and configure the virtual machine, please follow the instructions below:

1. Copy the file **PATRI.vdi**file on your PC/server
2. Open **Oracle VM Virtual Box** program
3. Click on the **New** icon to create a new Virtual Machine.


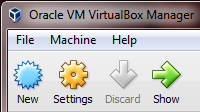


1. In the **Create Virtual Machine** window, insert the following parameters and click on **Next**
   - Name 🡪 PATRI
   - Type 🡪 Linux
   - Version 🡪 Red Hat (32 bit)


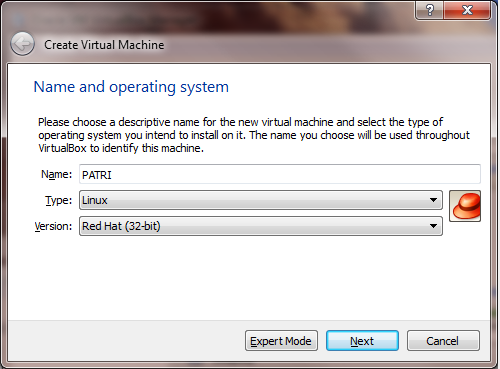


1. Select a memory size compatible with PC/server performance.To guarantee optimal system performance, **4 GB** are recommended. Click on the **Next** button to proceed with the installation.


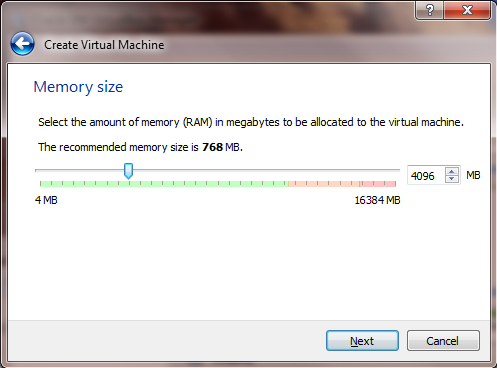


1. In the **Hard Disk** configuration page, select the **Use an existing virtual hard disk file** option and click on the
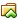
 icon.


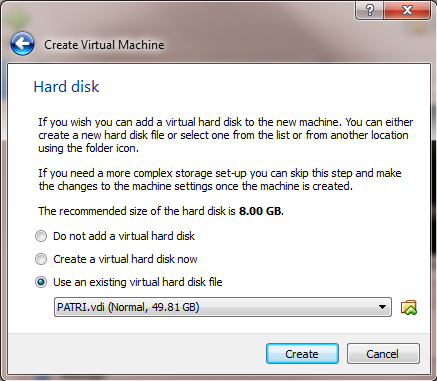


1. From the appropriate folder, select the PATRI.vdi file (previously imported in step 1) and click on the **Open** button. Click on the **Create**button to complete.


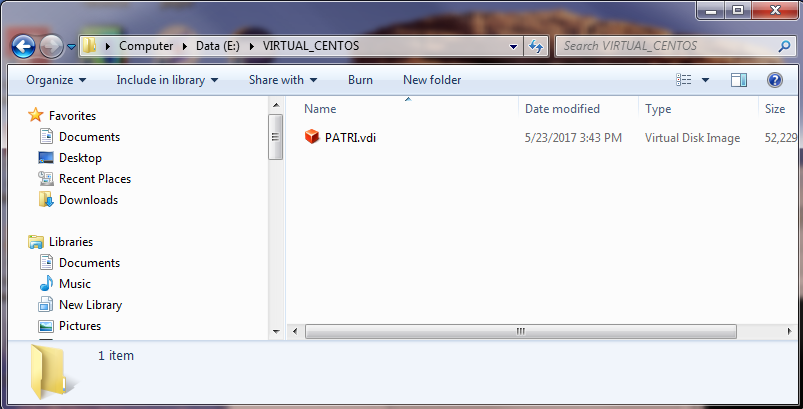


1. In the VM Virtual Box folder you can now find the PATRI virtual machine icon.

1. Click on **Setting** and then **Network** to configure the network:
   - Please ensure that the computer where PATRI is installed has an active internet connection. Charts and Volcano graphical functions use Google Charts libraries to generate charts available online.


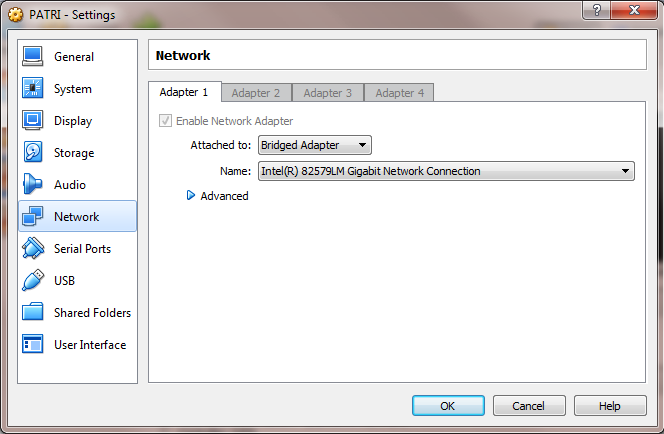


- - Set the **Attached to:** field to 🡪**Bridge Adapter**
  - Set the **Name:** field to 🡪 specify the installed Ethernet or Wireless board


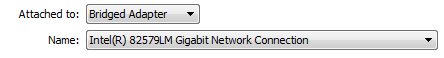


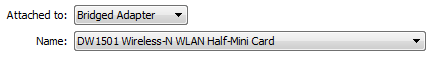


## Launch the Virtual machine

1. In the **Oracle VM Virtual Box Manager** window, click on the PATRI virtual machine icon and then on **Start**
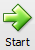
 .
2. PATRI execution window will open. This is a Linux server terminal. Please wait for the launch of the operative system(see figure below).


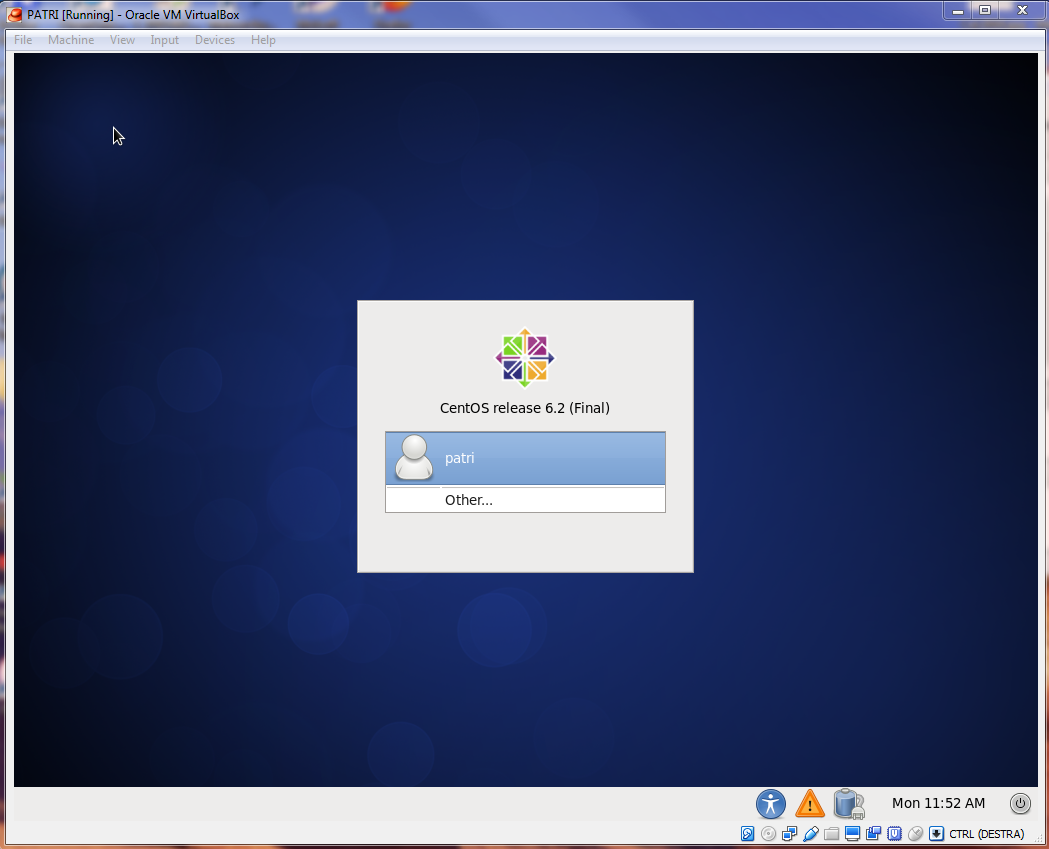


1. Click on Patri username type password = patri for system authentication; this will open the welcome PATRI page,available locally on the virtual machine, as follows:


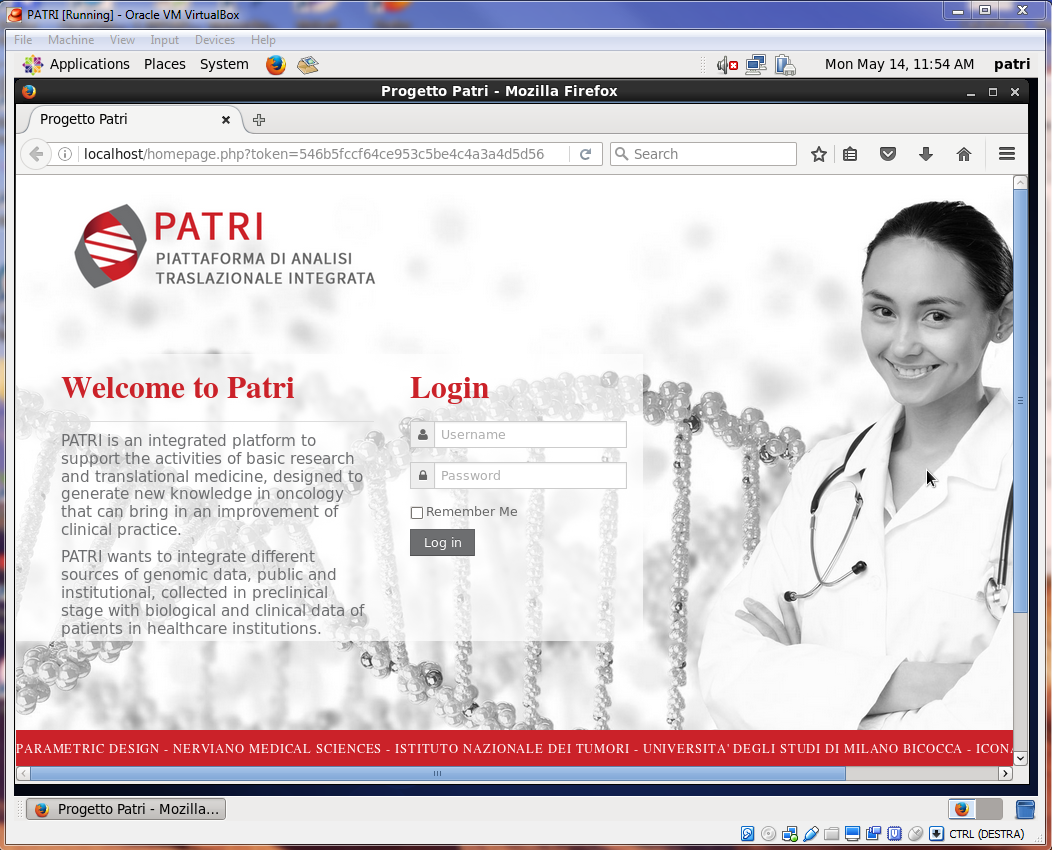


1. To use the virtual machine as a server, open the terminal and type the **ifconfig** command and copy the IP address that will be displayed on the screen.


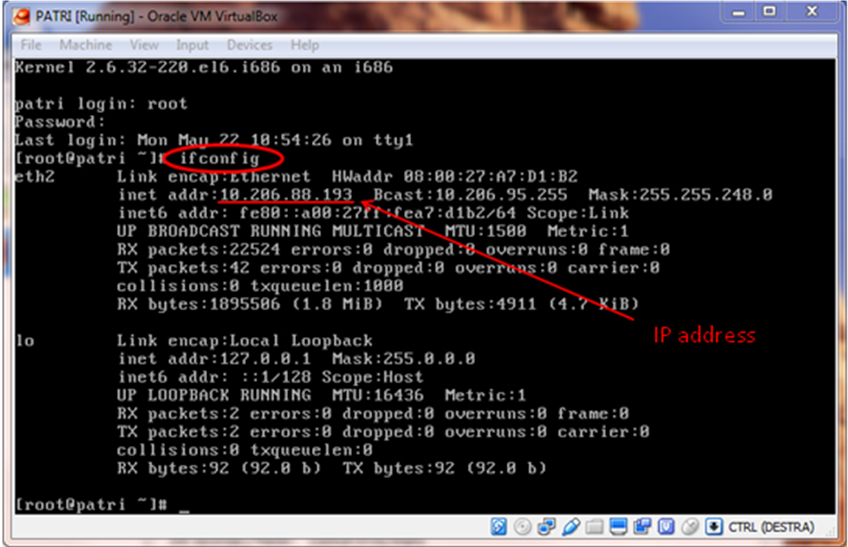


1. Open the web browser (see software requirement) and type the copied IP address in the URL


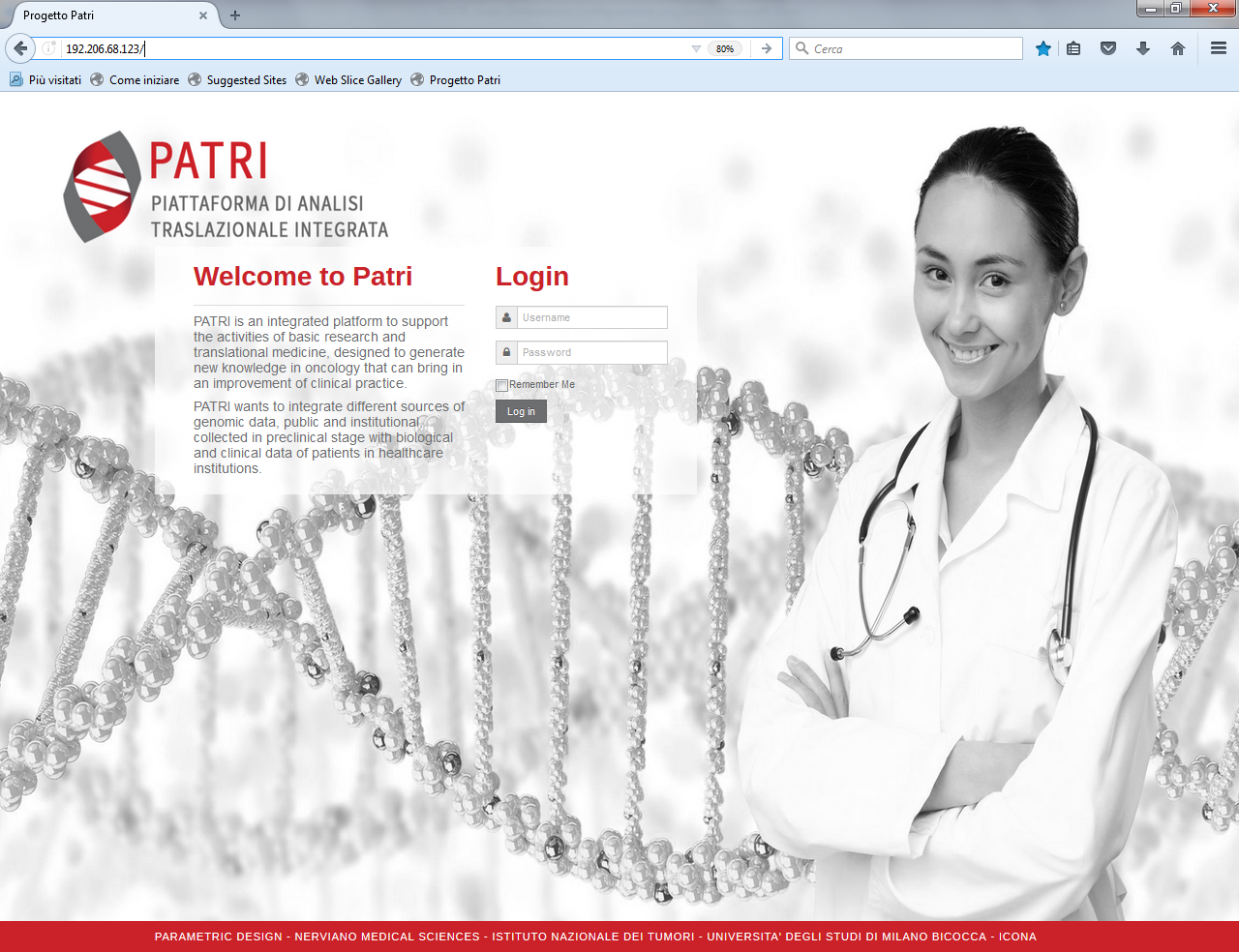


1. You can now use PATRI on your web browser.

Note: For instructions on PATRI usage please refer to *PATRI – User’s Guide*.

Note: Internet connection is required for the proper use of PATRI.

## Switch off virtual machine

1. Move to the virtual machine terminal
2. Type in the keyboard the **poweroff** command
3. Wait for the virtual machine to switch off

1. PATRI virtual machine is switched off when **Powered Off** appears in the **Oracle VM Virtual Box Manager** window.

# New User creation

To create a new user:

1. To access PATRI administrator page, type the IP address, obtained using the **ipconfig** command, followed by the keyword**/administrator** in the URL browser


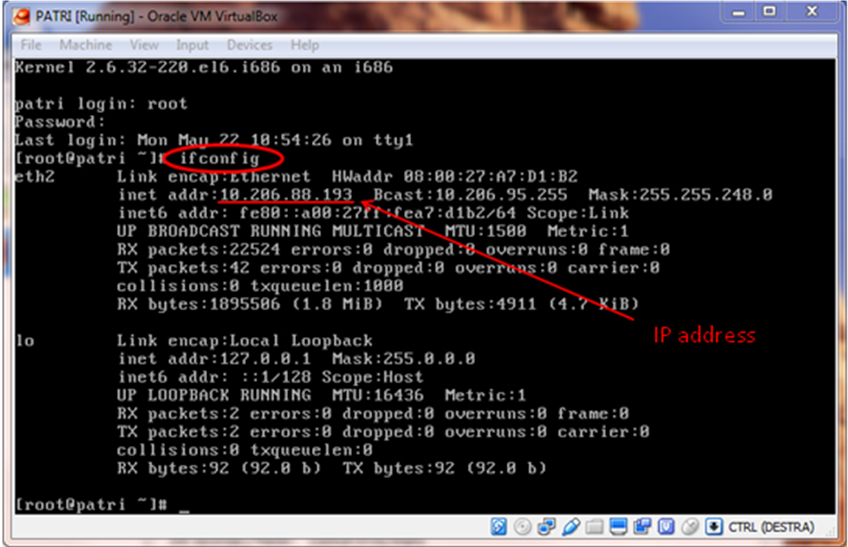


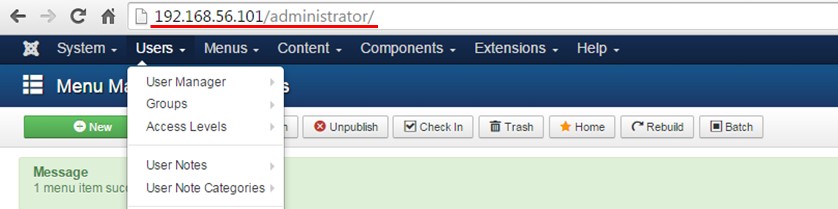


1. Log in to PATRI using the following credentials:

login: **admin**

password: **abc123**

1. Select **Users**🡪**User Manager** from the menu
2. Click on the **New** button and create username and password for the new user
3. Click on **save**

Suppl. File 2

#### PATRI

Platform User’s Guide

Version 1.0

DISCLAIMER NOTICE:

THE PATRI SOFTWARE IS PROVIDED "AS IS", WITHOUT WARRANTY OF ANY KIND, EXPRESS OR IMPLIED, INCLUDING BUT NOT LIMITED TO THE WARRANTIES OF MERCHANTABILITY, FITNESS FOR A PARTICULAR PURPOSE AND NONINFRINGEMENT. IN NO EVENT SHALL THE AUTHORS BE LIABLE FOR ANY CLAIM, DAMAGES OR OTHER LIABILITY, WHETHER IN AN ACTION OF CONTRACT, TORT OR OTHERWISE, ARISING FROM, OUT OF OR IN CONNECTION WITH THE SOFTWARE OR THE USE OR OTHER DEALINGS IN THE SOFTWARE.

**Table of Contents**

[Introduction 16](#_Toc514849239)

[Setup and installation 17](#_Toc514849240)

[System requirements 17](#_Toc514849241)

[Software requirements 18](#_Toc514849242)

[Virtual Machine Installation 18](#_Toc514849243)

[Launch the Virtual machine 23](#_Toc514849244)

[Switch off virtual machine 25](#_Toc514849245)

[New User creation 26](#_Toc514849246)

[Introduction 30](#_Toc514849247)

[Home page 31](#_Toc514849248)

[Analysis 32](#_Toc514849249)

[New Cell line Analysis: Resistant / Sensitive cell line labels 36](#_Toc514849250)

[New Cell line Analysis: Statistical Algorithm Selection 38](#_Toc514849251)

[Selected Cell Analysis 40](#_Toc514849252)

[Charts 45](#_Toc514849253)

[Heatmap 46](#_Toc514849254)

[Volcano Plot 46](#_Toc514849255)

[Scatter Plot 48](#_Toc514849256)

[*Fig. 19 Scatter plot* 48](#_Toc514849257)

[Clinical Samples 49](#_Toc514849258)

[New Sample Analysis: label of the samples 57](#_Toc514849259)

[New Sample Analysis: Algorithm choice 58](#_Toc514849260)

[Selected Sample Analysis 59](#_Toc514849261)

[Data visualization by Charts 60](#_Toc514849262)

[Import 60](#_Toc514849263)

[Import Expression 61](#_Toc514849264)

[Import Mutation 63](#_Toc514849265)

[Import Copy number 66](#_Toc514849266)

[Import Cell Line Description 67](#_Toc514849267)

[Import Gene Description 69](#_Toc514849268)

[Import Clinical Sample Description 70](#_Toc514849269)

# Introduction

The present document represents a reference for the use of the Platform for Integrated Translational Analysis or Piattaforma di Analisi Traslazionale Integrata (PATRI), which was conceived, designed and developed by Parametric Design, Nerviano Medical Science, Istituto Nazionale dei Tumori, Università di Milano Bicocca and Icona.

PATRI is an integrated platform designed to support basic and translational medical research activities, conceived to enable novel knowledge in the oncology research field aiming at providing improvements in the clinical field.

PATRI intends to achieve the integration of different sources of public and institutional genomics data, both as data collected during preclinical research phases and as biological and clinical data obtained for patients under treatment in clinical institutions.

The platform provides relevant support for the identification of novel prognostic and/or predictive biomarkers and for the identification of potential new therapeutic targets. Molecular data (such as gene mutation, expression, copy number) can be integrated with information about differential sensitivity to drugs under development and might possibly translate into the identification of putative biomarkers of response to pharmacological therapies.

Through the integration of biomolecular data collected in preclinical phase with biological and clinical data from patients treated in clinical institutions, PATRI can be employed in research activities to generate new knowledge and clinically useful information for the treatment of neoplastic pathologies.

Additional information regarding platform installation can be found in *PATRI – Platform Installation and Configuration Guide.*

# Home page


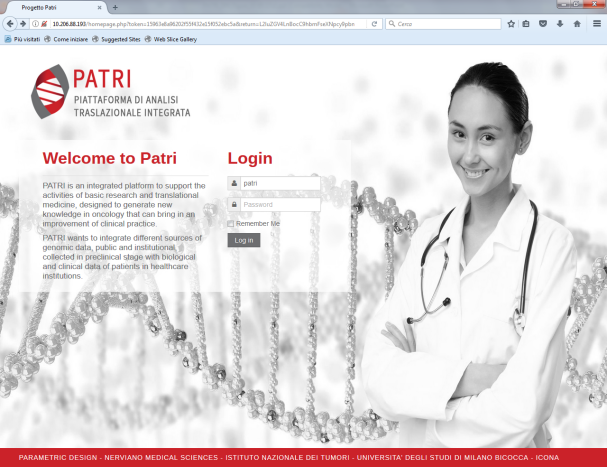


Fig. 1 - PATRI Home page

From PATRI home page users can login to PATRI platform (Fig. 1).

To login:

- Write **username** and **password** in the indicated fields
- Click on **Log in**
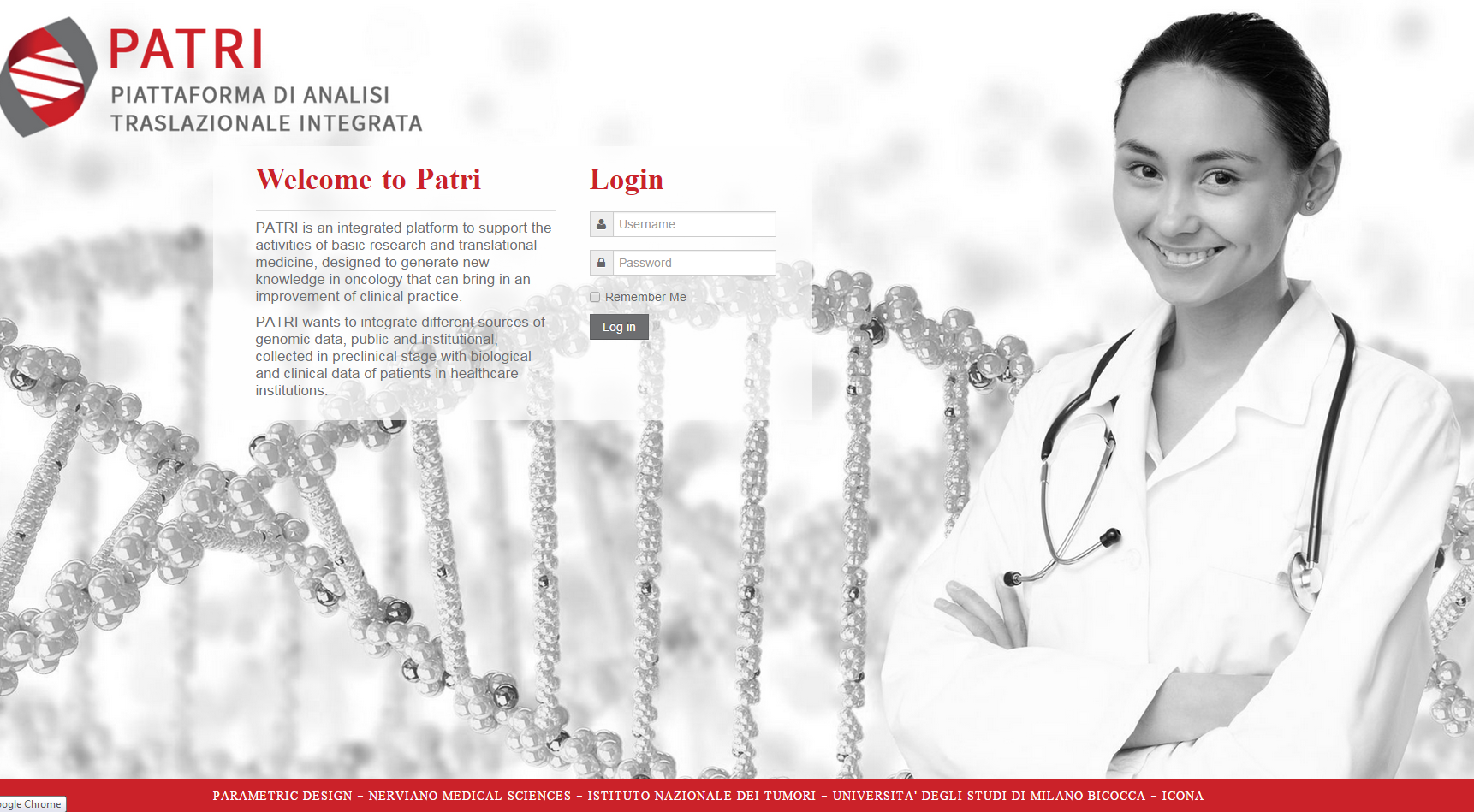


Username and password are requested to perform the login. If you do not have your own credentials, please use the following:

- User: **patri**
- Password: **patri**

To create a new user, please refer to the *PATRI – Platform Installation and Configuration* manual.

Note: For issues relative to page display, please refer to *PATRI – Platform Installation and Configuration* manual.

# Analysis


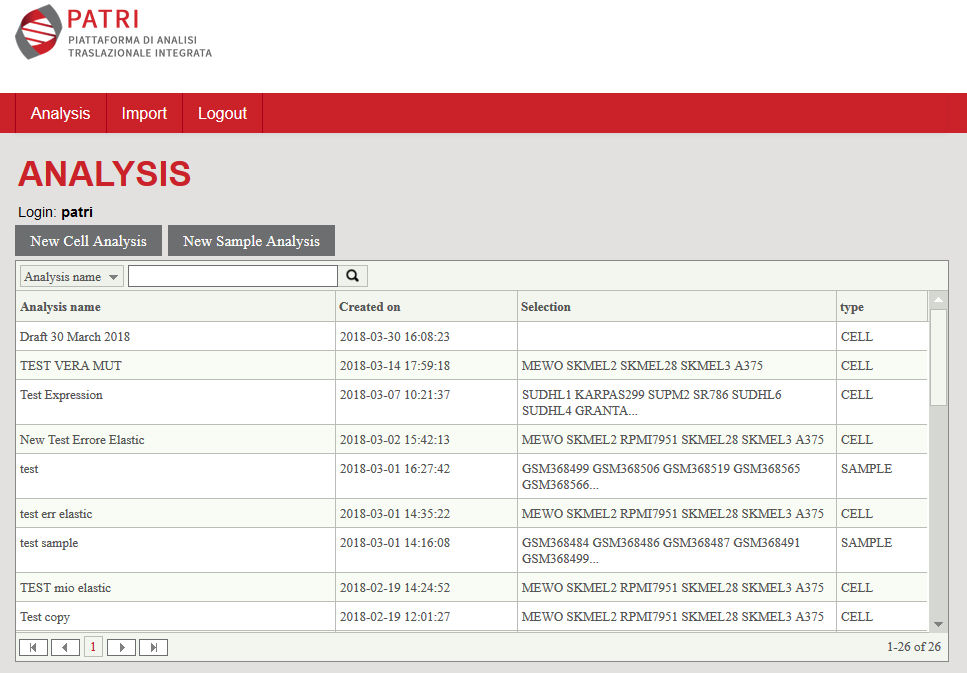


Fig. 2 – ANALYSIS page

Upon login, the Analysis page will be displayed. It allows the following procedures:

- Import genomics dataset for the analysis
- Open a previously saved analysis
- Create a new analysis

By clicking the **Import**
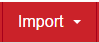
 button, a page allowing the upload of external genomic datasets for the analysis will be opened. Please refer to the ‘Import’ chapter for details on data import procedures.

Saved analyses are listed. To open a previously saved analysis, click on the row of interest.

Table fields:

- **Analysis Name** = name identifying the analysis (as provided by the user). Unfinished and not saved analysis will be automatically saved with the name “Draft” followed by the analysis creation date
- **Created on** = analysis creation date
- **Selection** = names of the cell lines or clinical tumor samples used for the analysis
- **Type** = it indicates if the analysis has been performed starting from cell lines or clinical samples

The search box
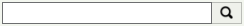
 allows searching previously performed analyses by keywords.

**Two major options are available to run analyses: Cell Line Analysis or Sample Analysis.**

The **New Cell Line Analysis
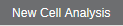
** button allows creating a new analysis based on sensitivity data on cancer cell lines. The **New Sample Analysis**
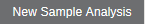
 button allows performing a similar analysis starting from information on patients’ therapeutic response to a treatment on clinical sample data.

**New Cell Line Analysis**


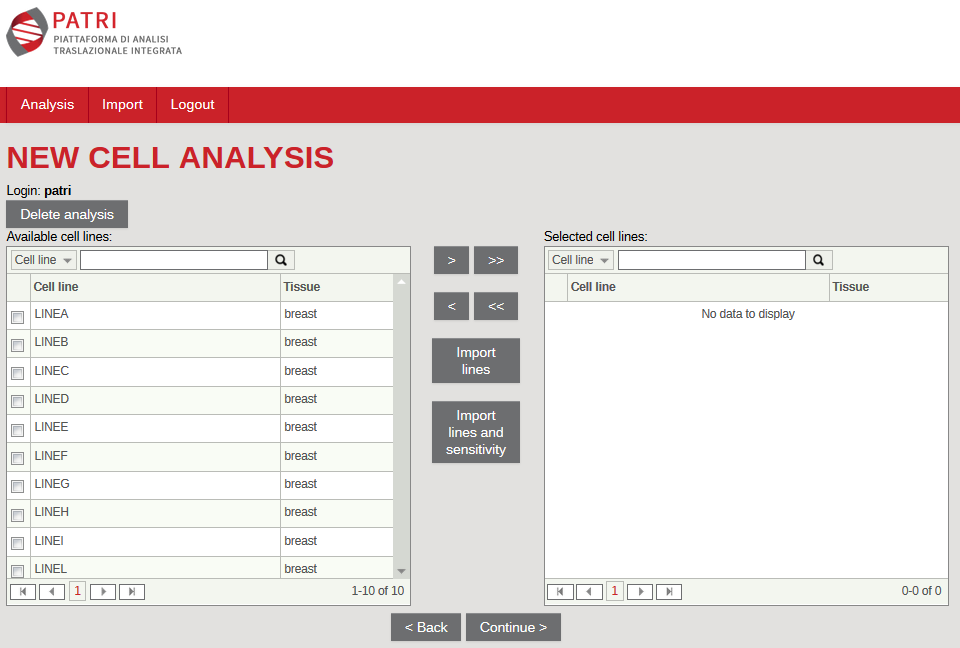


Fig. 3 – NEW CELL LINE ANALYSIS: cell line selection

The “New cell line analysis” page can be accessed from the Analysis page either upon creation of a new analysis or by loading a previously saved analysis. It allows the selection of the cell lines for the subsequent analyses.

Cell line import can be performed in three ways:

1. Graphical selection:

- Select the cell lines from the provided list in the **Available cell lines** list by inserting the tick on the corresponding row
- Move cell lines from **Available cell lines** to **Selected cell lines** using the
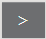
 button.

Note: The
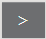
 button allows to move cell lines back to the **Available cell lines table**

Note: By using the
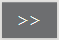
 and
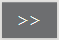
 buttons, the user can move all the cell lines from **Available cell lines** to **Selected cell lines** and vice versa.

1. Text selection:

- Click on **Import Lines** button
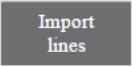

- Insert the space-delimited names of the cell lines in the provided form
- Click **Import Lines**

1. From file (including the label on sensitivity/resistance to the drug):

- Click the **Import Lines and Sensitivity** button
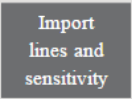

- Select a txt file (tab-delimited) reporting on each row the names of one cell line and the respective label “sensitive” or “resistant” (see the provided files for testing purposes)
- Click **Import lines**

The cell lines selected for the analysis will be displayed in the **Selected cell lines table**.

To continue with the analysis, click on the
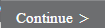
 button.

The **Delete Analysis** button
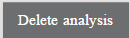
 on the top left allows deletion of the current analysis.

#

# New Cell line Analysis: Resistant / Sensitive cell line labels


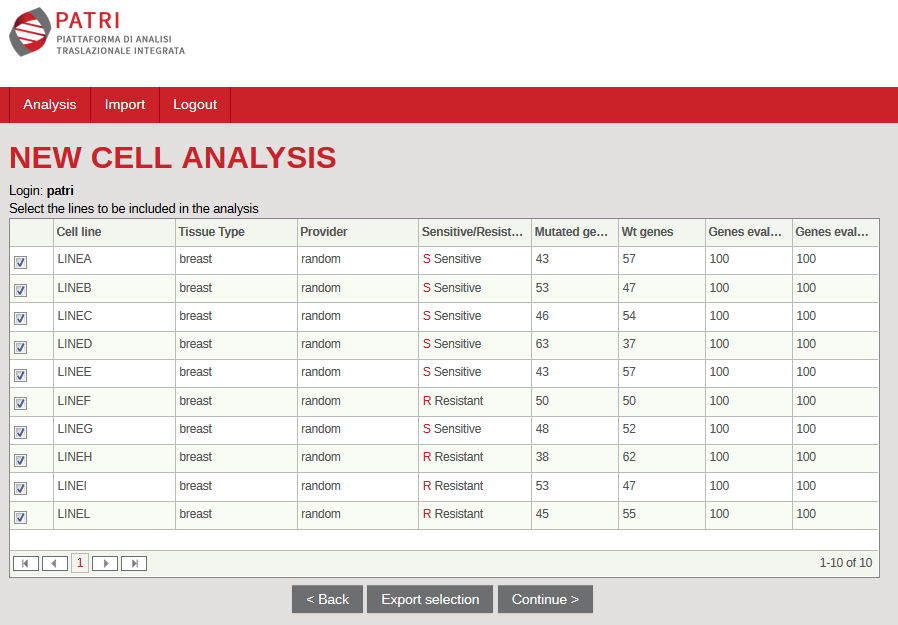


Fig. 4 – NEW CELL LINE ANALYSIS: selection of cell lines for the analysis

In this page, the user can assign the “sensitive” or “resistant” label to the cell lines, if not already provided by importing a .txt file (see previous page), and, if desired, remove selected cell lines from the analysis by following the steps reported below:

- To assign the Sensitive (S) or Resistant (R) label to each cell line, click on the red capital letter in the Sensitive/Resistant column
- The cell lines with a tick
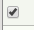
 will be included in the subsequent analysis. Please deselect if you wish to exclude a cell line from the analysis. This field cannot be selected if the Sensitive/Resistant column value is **Null**.

Table fields:

- Name of the cell line
- Cell line tissue of origin
- Cell line provider
- **Sensitive/Resistant** column: it is mandatory field to specify if the cell line is considered sensitive or resistant to the drug or other treatment.
- Number of gene variants reported in the dataset loaded in PATRI for the indicated cell line (**Mutated genes**).
- Number of wild-type genes reported in the dataset loaded in PATRI for the indicated cell line (**Wt genes**).
- Number of genes profiled by gene expression (**Genes evaluated for expression**) or Copy Number (**Genes evaluated for Copy Number**).

Please note:

1. At least 2 Resistant and 2 Sensitive cell lines must be selected to continue the analysis.
2. Elastic Net model (it can be selected in the following page) requires at least 4 Sensitive and 4 Resistant cell lines.

Note: Click on **Export Selection** button to download the list of cell lines included in the **Selected cell lines** table in txt format.

Click on the following button
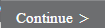
 to continue with the statistic model selection.

#

# New Cell line Analysis: Statistical Algorithm Selection


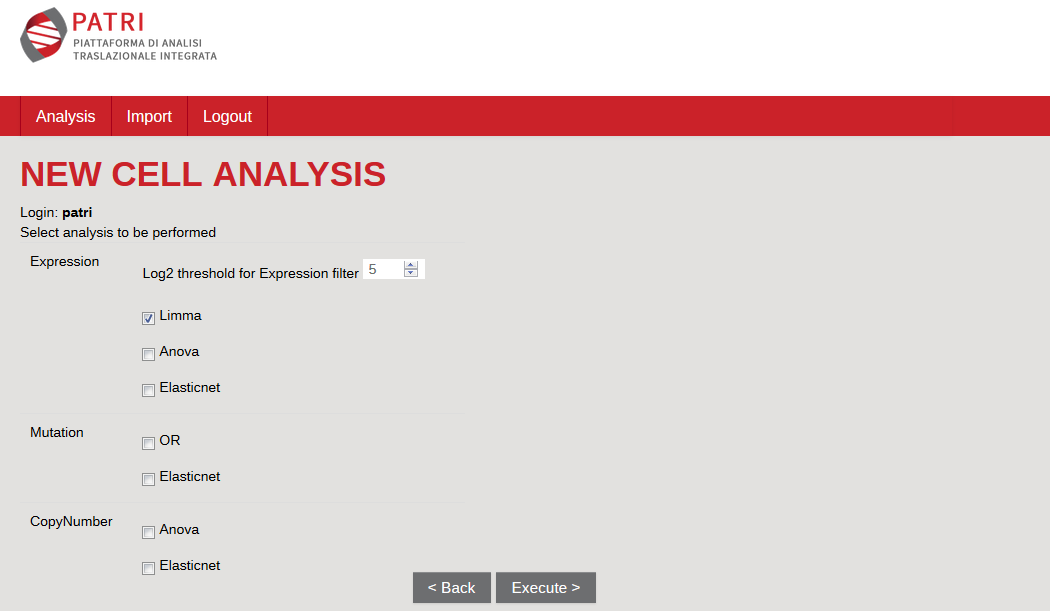


Fig. 5 – New Genomic Analysis: statistic model selection

The page allows the selection of the statistics for the analysis.

Based on the data type, the available algorithms can be selected:

Expression:

- Limma or Anova
- Elasticnet (4 Resistant and 4 Sensitive cell lines required)

Mutation:

- Odds Ratio (OR)
- Elasticnet (4 Resistant and 4 Sensitive cell lines required)

Copy Number:

- Anova
- Elasticnet (4 Resistant and 4 Sensitive cell lines required)

Click on the Execute
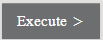
 button. A new window will open. Please type a name for the analysis in the **Analysis Name** field.


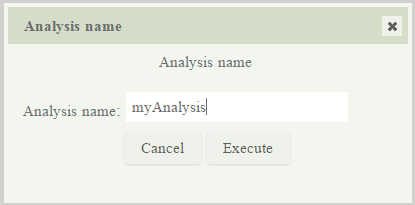


Fig. 6 – Analysis name window

Click on Execute
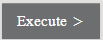
 to launch the analysis. A log page will appear (Fig. 7). Upon execution completion, the Execution completed red sentence will appear on the screen.


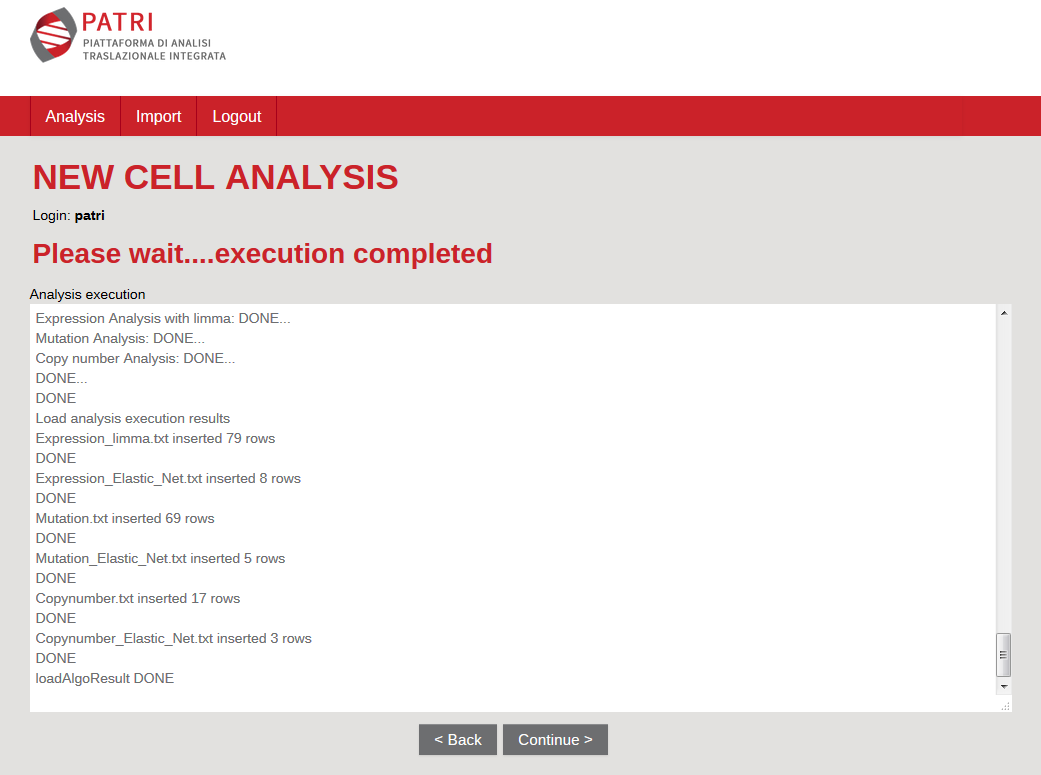


Fig. 7 – Analysis execution log page

Click on **Continue**
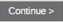
 and go to the Result page.

# Selected Cell Analysis


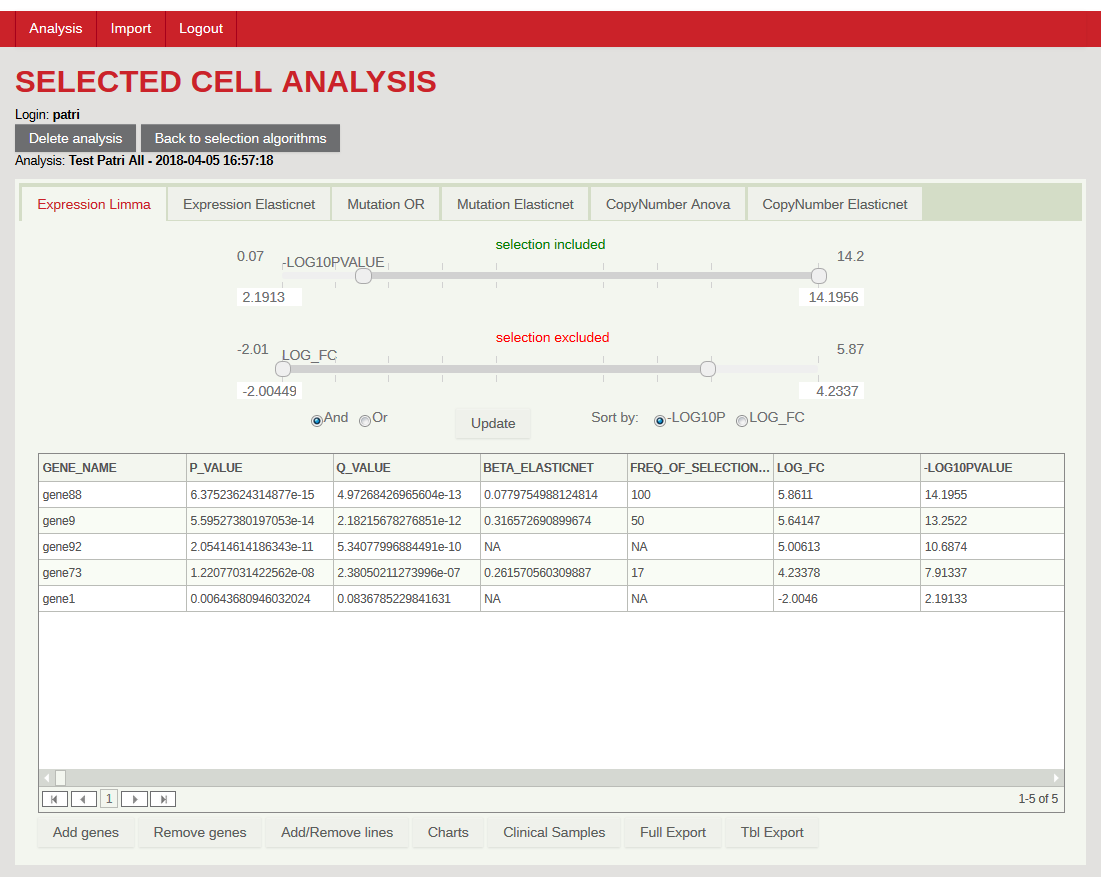


Fig. 8 – SELECTED CELL ANALYSIS: Example of results obtained from Gene Expression analysis using Limma

In this page, results from the selected analysis are reported, organized in sheets (i.e. Gene Expression by Limma in Fig. 8). Each sheet corresponds to a genomic type of analysis (Gene expression, Mutation, Copy Number) performed using selected statistics (Limma, Anova, ElasticNet).

Table Fields:

**GENE_NAME**: name of the gene

**P_VALUE**: p-value referred to the used statistics

**Q_VALUE**: q-value calculated by FDR

**BETA_ELASTICNET**: angular coefficient referred to the gene in the Elastic Net model (please also see Selected Genomics analysis: Elastic Net).

**FREQ_OF_SELECTION_IN_100_EN_MODELS**: number of times a gene is found as a significant covariate in the model (please also see Selected Genomics analysis: Elastic Net).

**LOG_FC**: LogRatio Sensitive vs. Resistant

**LOG10PVALUE**: -Log(P_VALUE)

When mutation analysis is performed, in the **Mutation OR** sheet all the fields are equal to the ones reported above with the exception of **CORRECTED_ODDS_RATIO** field that replaces the **LOG_FC** field. This field shows the Corrected Odds Ratio value. An example of Mutation OR sheet is reported in Fig. 9.


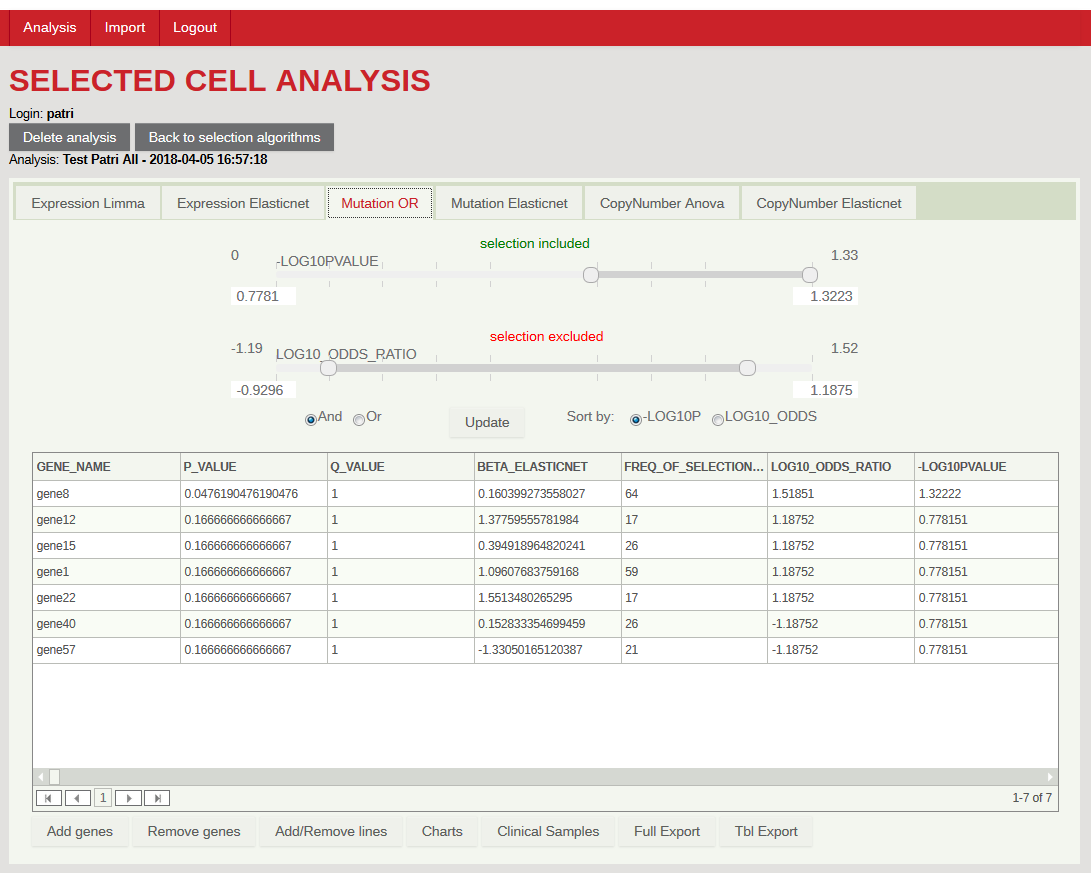


Fig. 9 - SELECTED GENOMICS ANALYSIS: Example of results from OR mutation analysis

Genes identified in the differential analysis can be sorted by p-value or logFC (LR) / log10Odds.

Two slide bars above the table allow gene filtering based on -LOG10PVALUE and LOGFC (or or LOG10_ODDS_RATIO for Mutation Analysis). Extreme values for the distributions of both -LOG10PVALUE and LOGFC or LOG10_ODDS_RATIO in the analyzed data are shown at each end of the sliding bars.

Sliding buttons
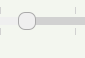
 are provided to filter results; they are set by default on unfiltered values:

- For the –Log10PValue slider, default position is on the extreme values of the distribution. Values included between the two sliding buttons
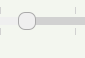
 are retained in the filtered data (Fig 10)
- For Log_FC or Log10_Odds_ratio sliders, default position is central. Values included between the two sliding buttons
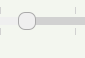
 are excluded from the filtered data (Fig. 11)

**
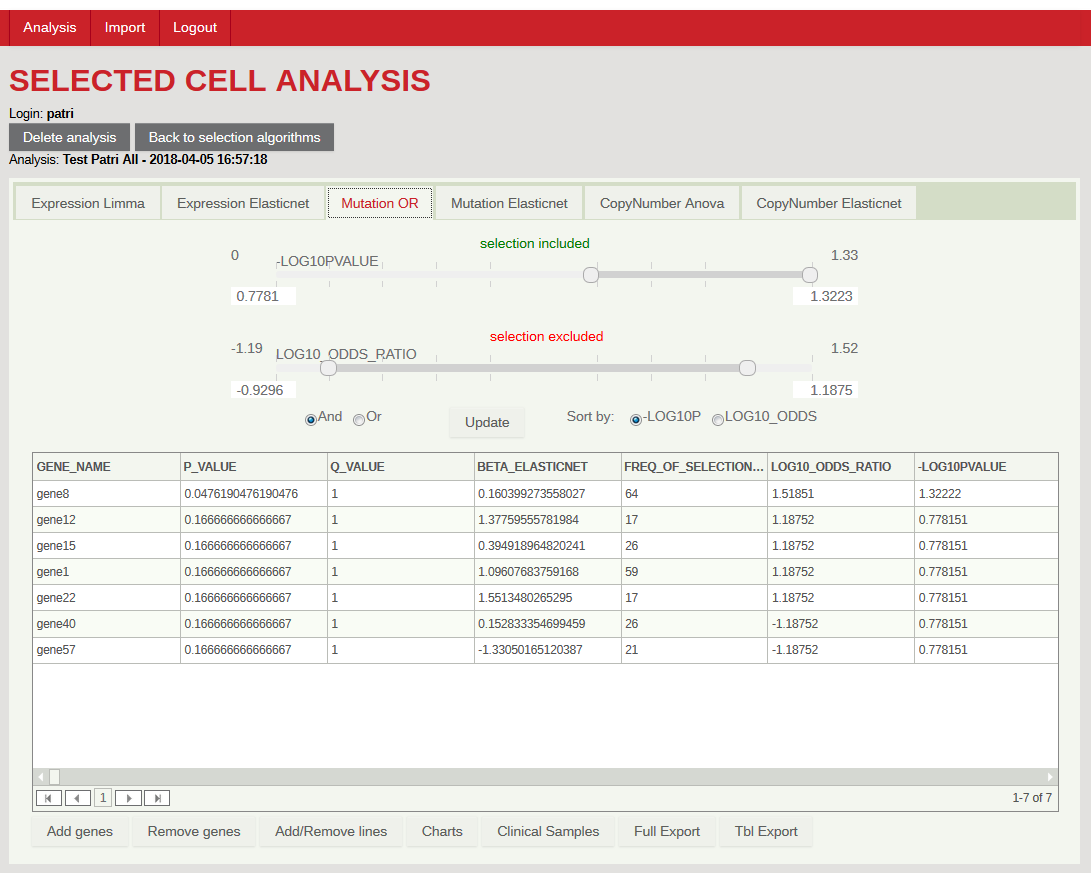

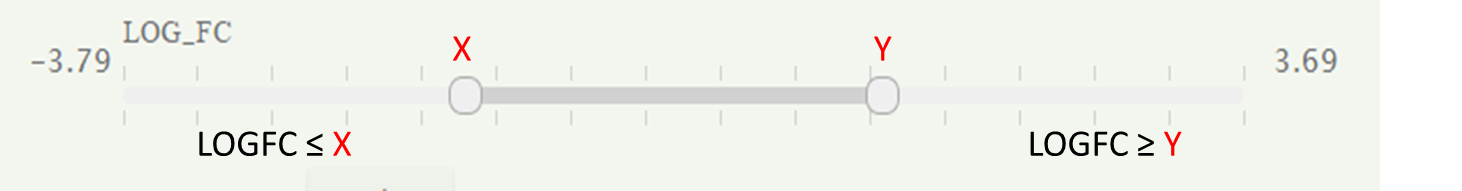
**

Fig.10 - slider LOG_FC (similar to LOG10_ODDS)


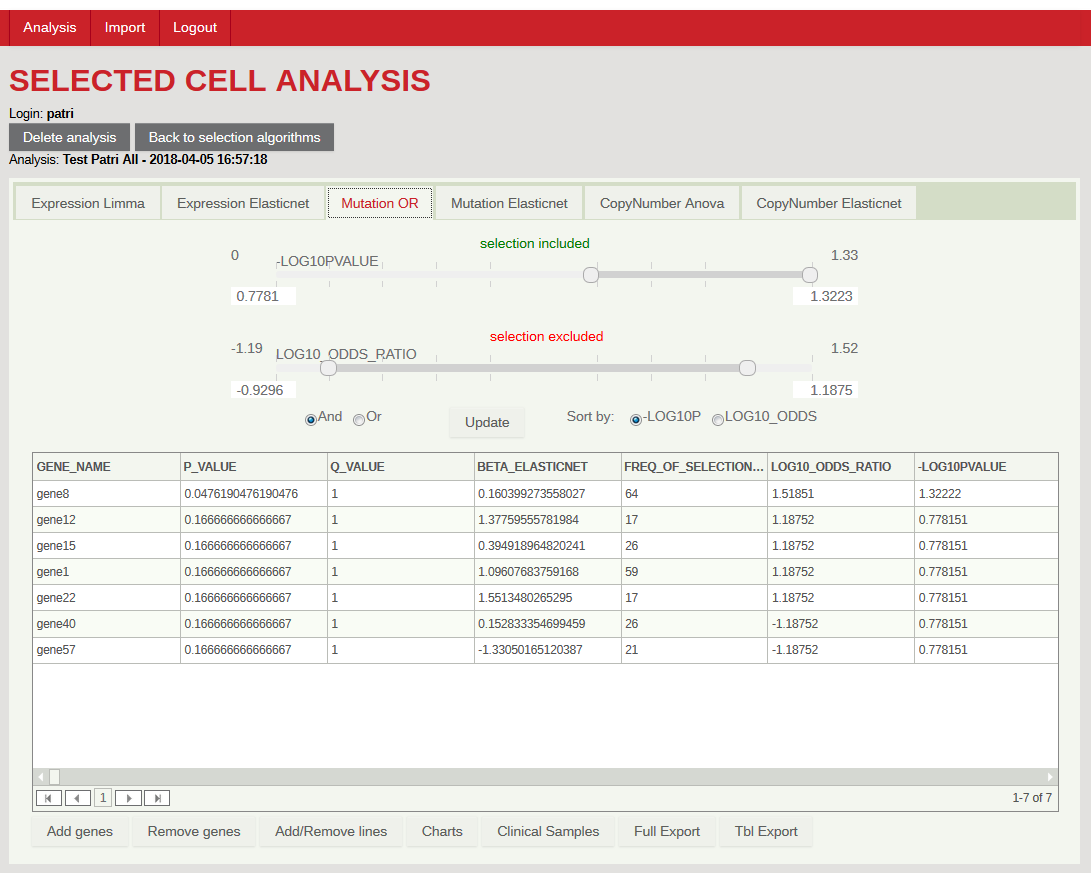

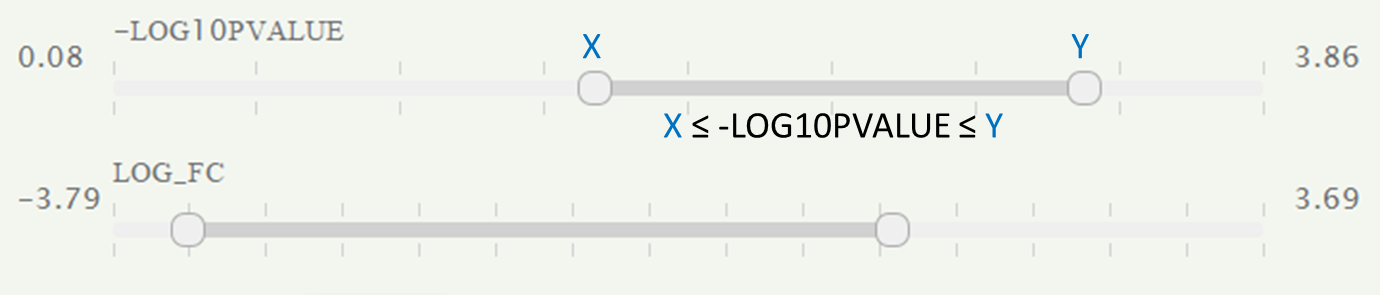


Fig.11 - slider –LOG10PVALUE

To change filtering thresholds, users can:

- Move the sliding buttons
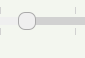
 on the right or on the left: the corresponding new threshold values will be visualized in the white boxes below the bar extremities. Click on
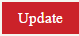

- Type desired threshold values in the white boxes below the slide bar extremities, then click on
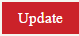
 to move the sliding buttons
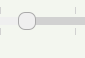
 accordingly
- Filtering options can be combined by selecting the ‘And’ or ‘Or’ logical operator radio buttons followed by
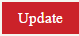


On the bottom of the results pane, additional functions can be activated by clicking on the buttons shown in Fig. 12:

**Add genes**: add one or more genes having out of threshold range values to the filtered list

**Remove genes**: remove one or more genes from the filtered list.

**Add/remove lines**: add/remove cell lines from the list of cell lines included in the analysis: this function defines the cell lines whose genomics data will be displayed in the plots generated using the **Charts** button.

**Charts**: opens a new web page where results included in the table can be visualized in several graphical options

**Clinical Samples:** this button initiates a further statistical analysis: the filtered results obtained for cell lines will be used for statistical exploration on genomics datasets on clinical samples, if available, and subsequently associated and integrated with clinical annotations (see [Clinical samples](#_Clinical_Samples))

**Full Export**: export of all the data in txt format

**Tbl Export:** export of the filtered data in txt format


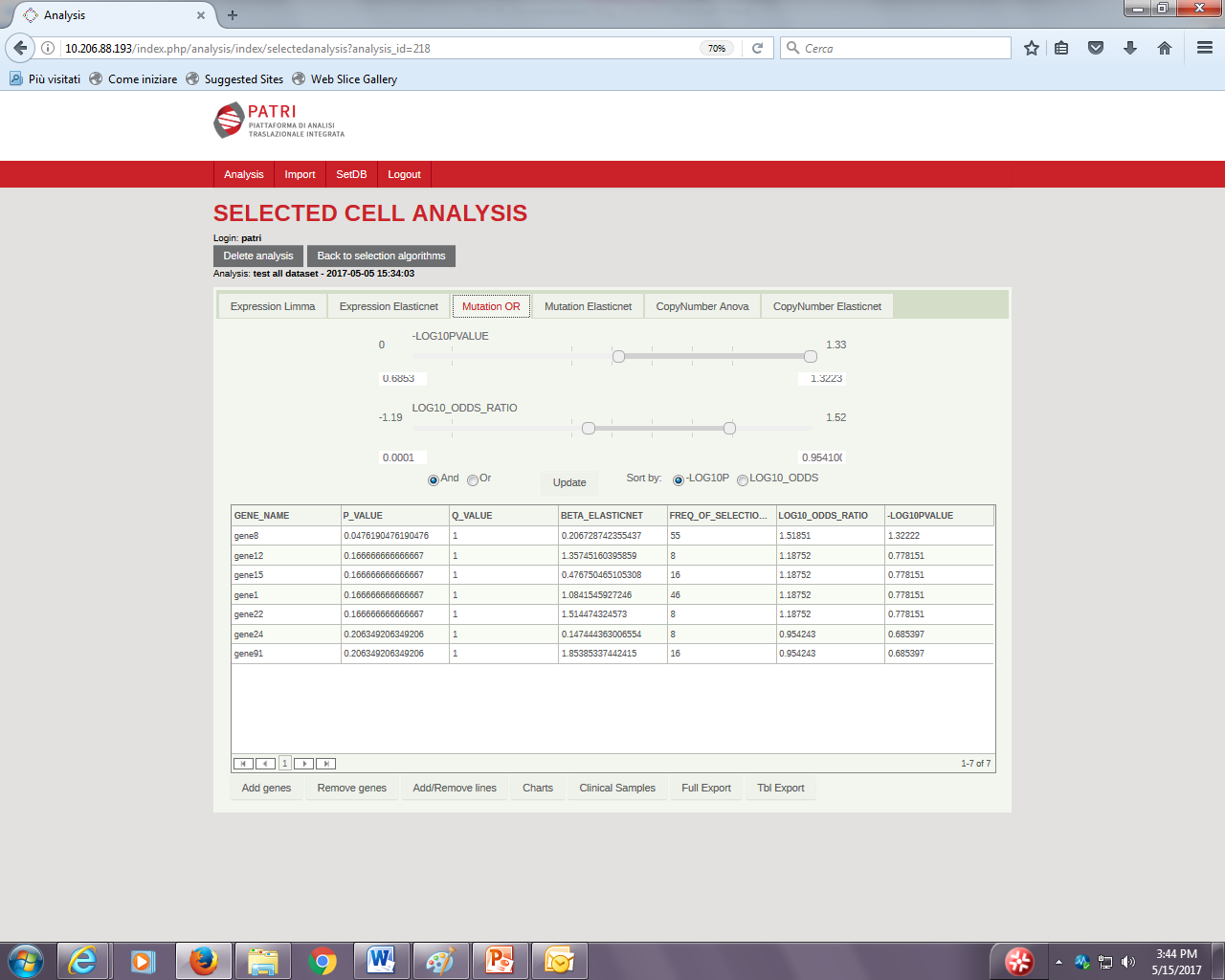


Fig. 12 - Footer commands

Note: please ensure that the computer where PATRI is installed has an active internet connection. Charts and Volcano graphical functions use Google Charts libraries to generate charts available online.

Users' computers must have access to https://www.gstatic.com/charts/loader.js in order to use the interactive features of Google Charts.

A custom implementation of the elastic net algorithm, a machine learning approach originally used in Barretina J, et al. for the simultaneous analysis of gene expression, copy number and mutation data is also included. To increase robustness, the algorithm was adjusted in PATRI to be run 100 times under different bootstrap samples and obtain a stable ranking of the final genes; the percentage of times a molecular feature is retained in the model is reported, accompanied by the average beta value across runs, as reported in Fig. 14.

In the elastic net results page, using the sliders in the upper part of the panel data can be filtered by the user using the following parameters:

- **BETA_EASTICNET**
- **FREQ_OF_SELECTION_IN_100_EN_MODELS**.


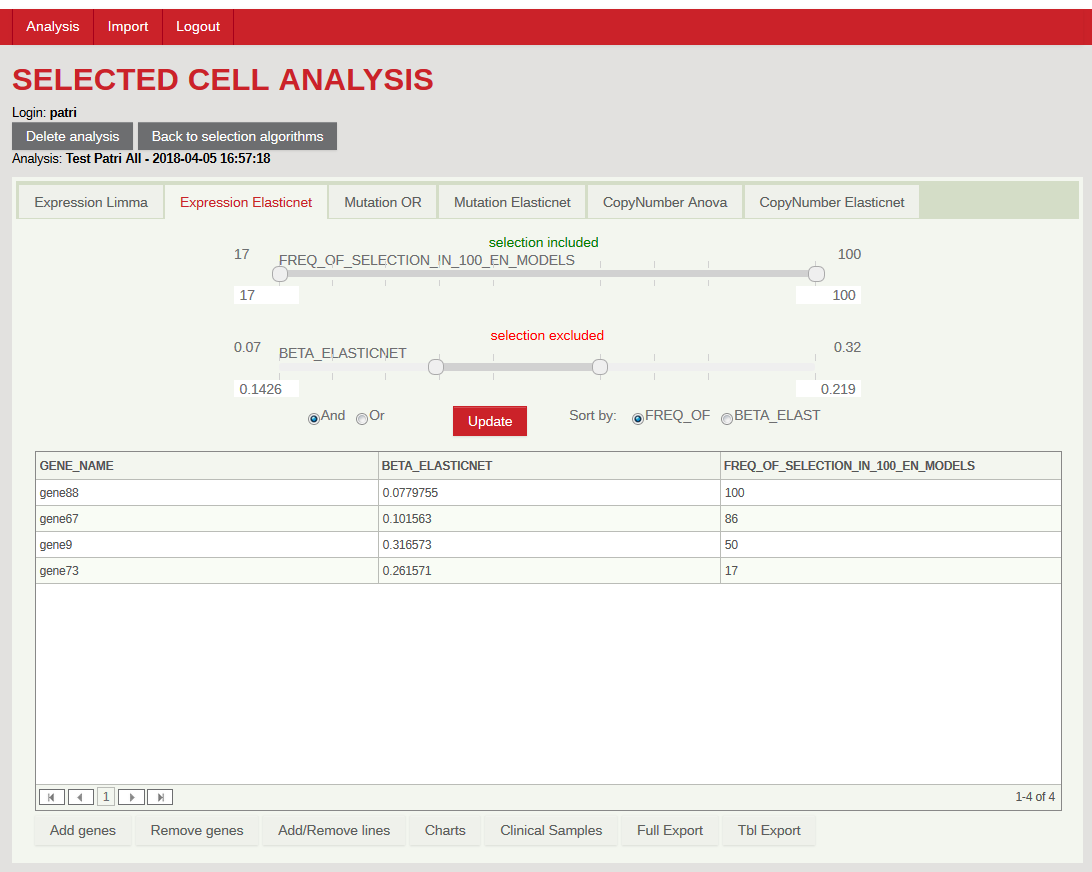


Fig. 13 - Elastic Net page: gene expression example

# Charts

To graphically visualize the results of the analysis click
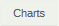
 in the SELECTED CELL ANALYSIS result page. Three types of graph can be displayed:

- **Volcano Plot**
- **Dendro HeatMap Plot**
- **Scatter Plot**

The charts use in input the data filtered from the result page. In the left panel (**Plot Generation Panel**) the commands to select the desired plots are reported, in the right panel a plot viewer is displayed upon selection of the graph type (**Plot Viewer**).


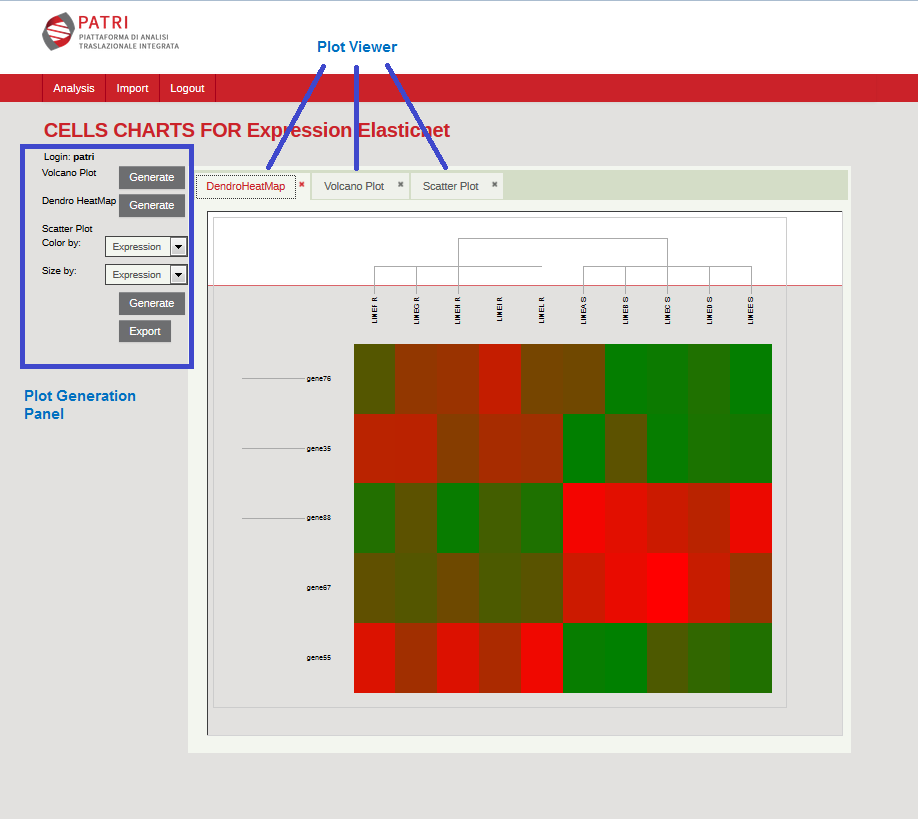


Fig. 14 – Page showing CHARTS available for the visualization of the results obtained from Limma gene expression analysis

To select and display in the **Plot Viewer** the desired plot, Click on the Generate button
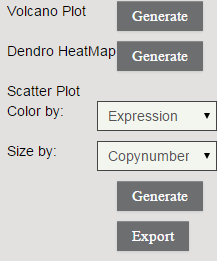
.

Data used to generate the graph can be downloaded in txt format by click on the Export button
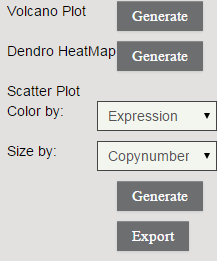
 in the **Plot Generation Panel** menu.

# Heatmap

The **Dendro heatmap** plot (Fig. 15) displays the genes in a colored matrix ranging from green to red, accordingly to gene expression level (green=low, red=high).

Zoom in/out options allow customizing the visualization of both genes and cell lines.

# Volcano Plot


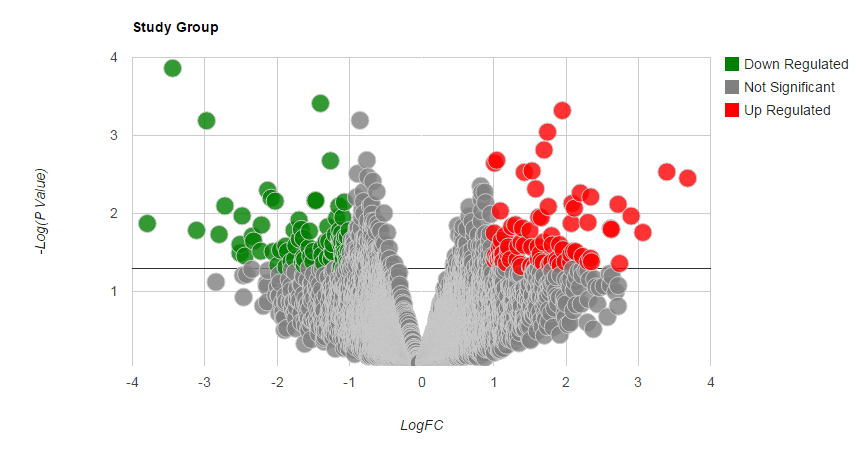


Fig. 15 – Volcano Plot: default view

The Volcano Plot is an interactive chart where each gene is represented by a circle in a Cartesian plan with the X axis corresponding to log(FoldChange) and y axis to –log(p-value).

Green circles indicate genes with –Log(PValue) > 1.31***** and LogFC < -1 (Down modulated).

Red circles indicate genes with –Log(PValue) > 1.31***** and LogFC > +1 (Up modulated).

***** : p-value = 0,05

Plot navigation is dynamic by mouseover and scrolling functions, allowing to zoom in and out and to move left and right by holding and releasing mouse left-click (Fig. 16-17)


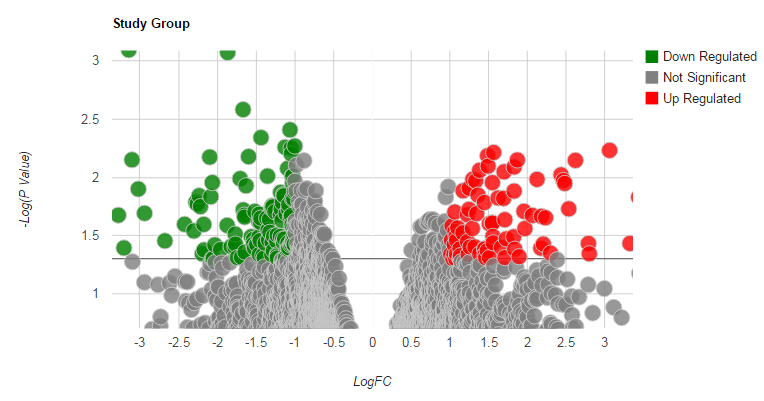

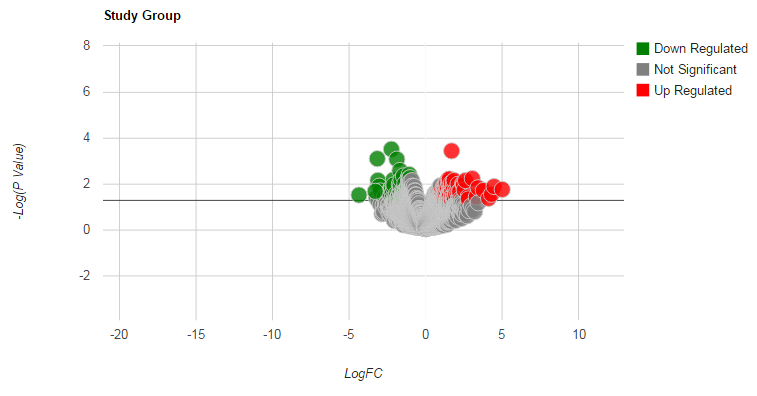


Fig. 16 – Volcano Plot: zoom-in and zoom-out views (by mouse scroll)

In addition, gene information such as gene symbol, LogFC, -Log(p-value) and class status can be visualized by mouseover (Fig. 18)


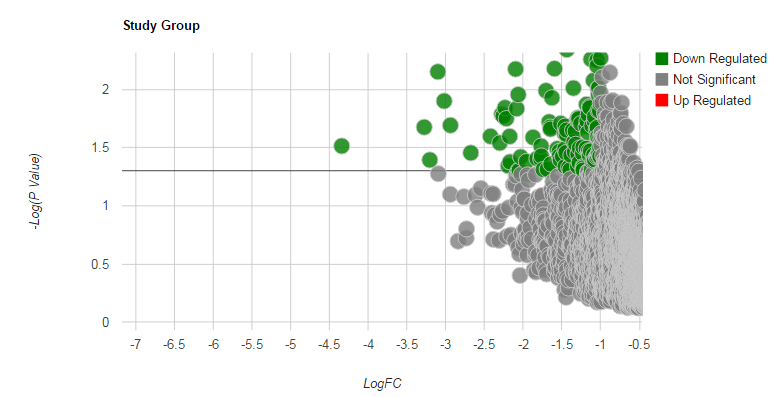


Fig. 17 13 – Volcano Plot: side views (mouse left-click)


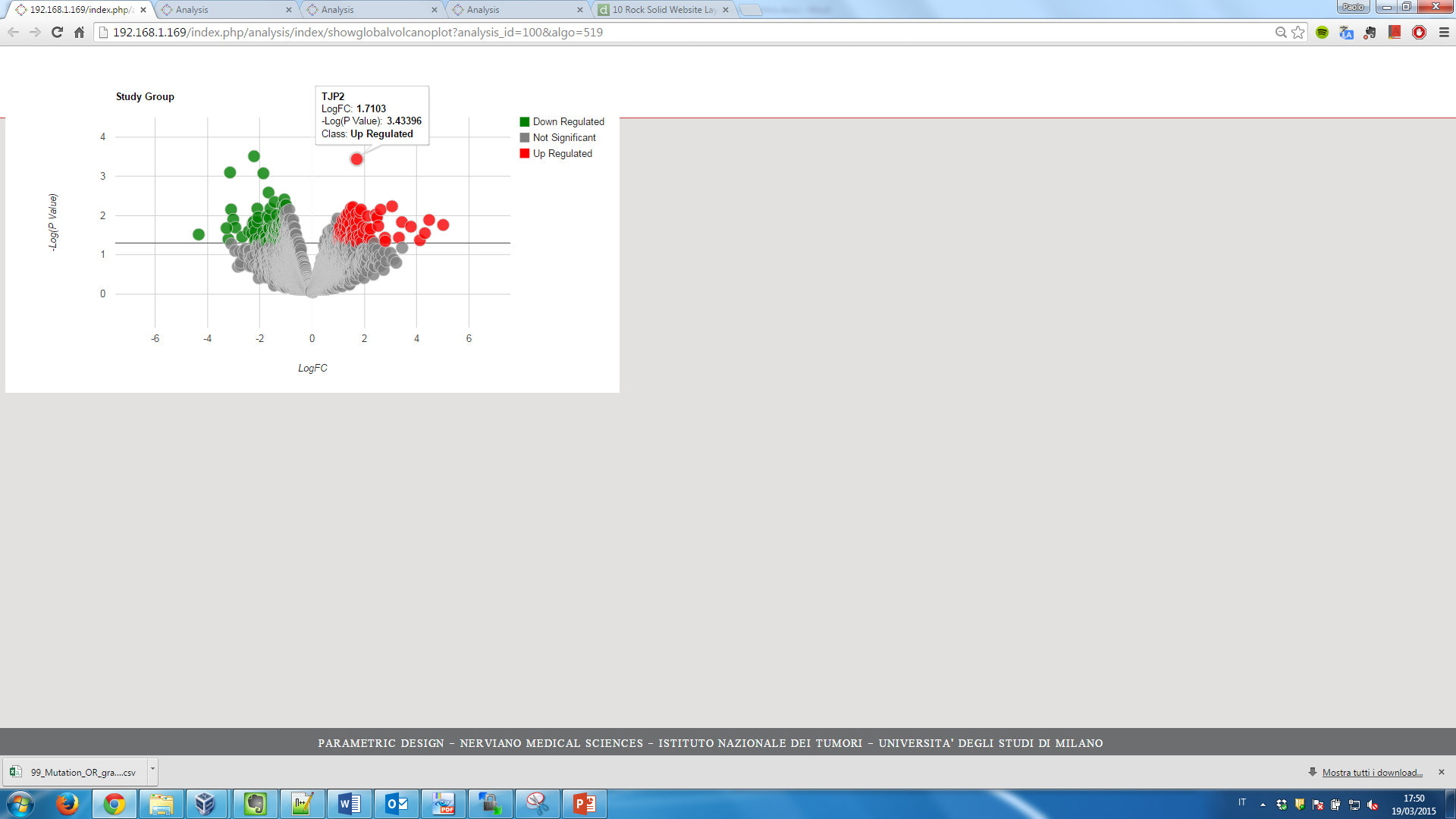


Fig. 18: Volcano Plot: - gene information (mouseover)

# Scatter Plot

In the Scatter Plot visualization, two molecular characteristics can be displayed simultaneously for the panel of selected genes. Color and dimension features of the dots can be set to represent mutations, gene expression or copy number features (Fig. 19).

Zoom in/out options allow customizing visualization of both genes and cell lines.


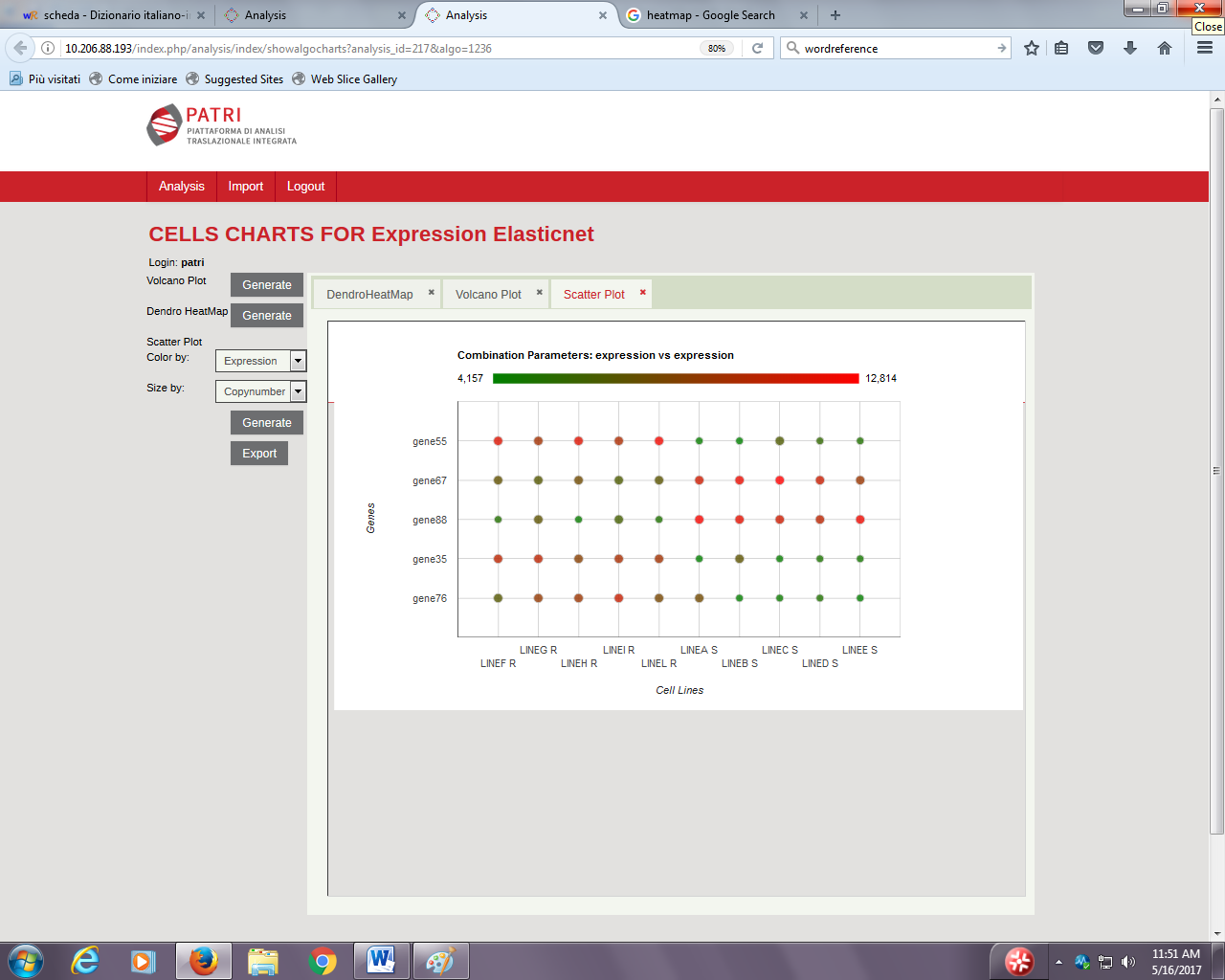


# *Fig. 19 Scatter plot*

# Clinical Samples

This page, accessible through the Selected Cell Analysis result page by click on the Clinical Samples
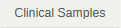
 button, allows to mine clinical sample data, if available, by assigning each sample to either ‘resistant’ or ‘sensitive’ subpopulations based on gene profiles selected in the (mutation, copy number, gene expression) via random forest algorithm evaluation.

To start the analysis, select a dataset from the provided drop down menu. In this menu user pre-loaded clinical datasets will be displayed. Press the Execute button
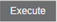
 (Fig. 20).


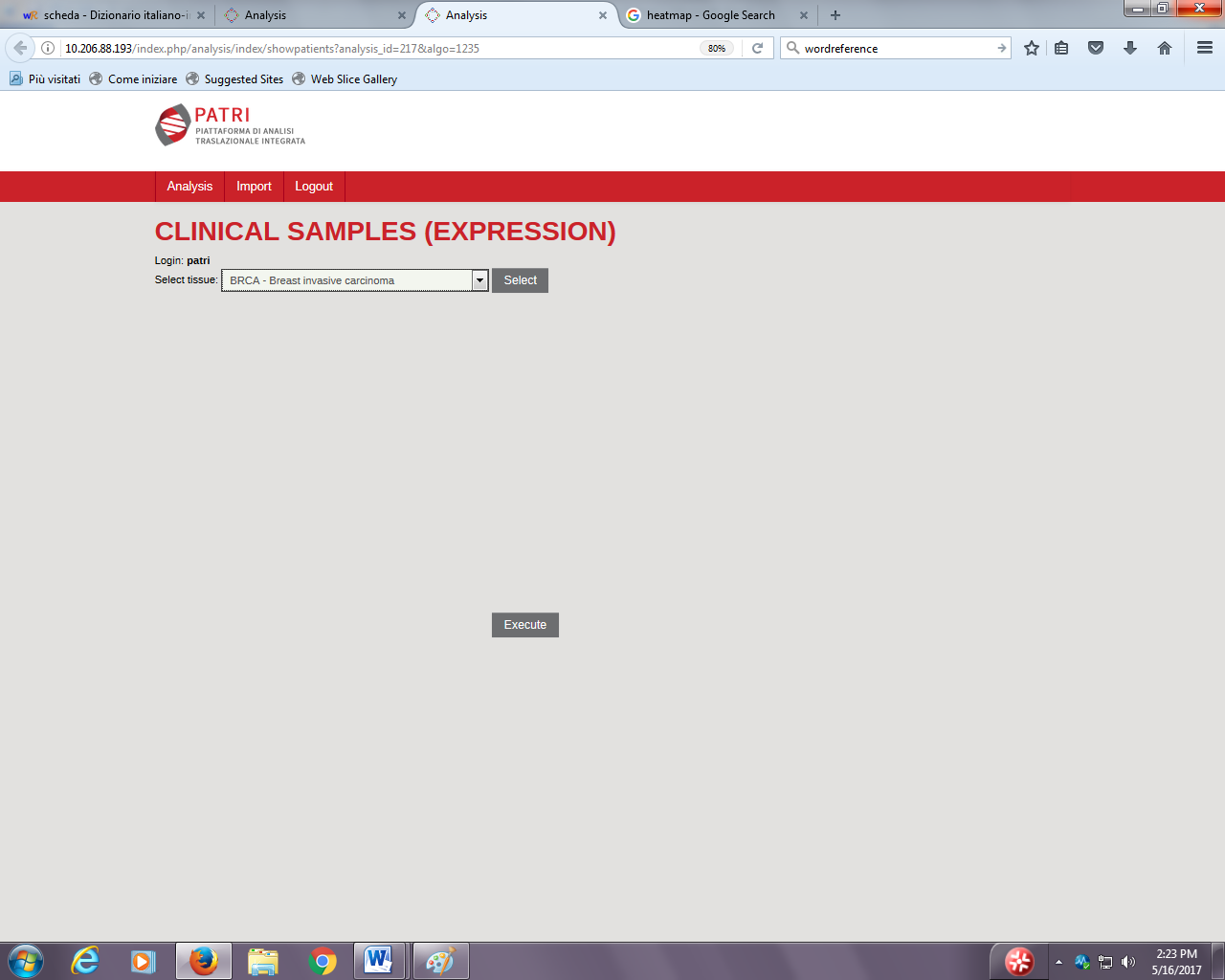


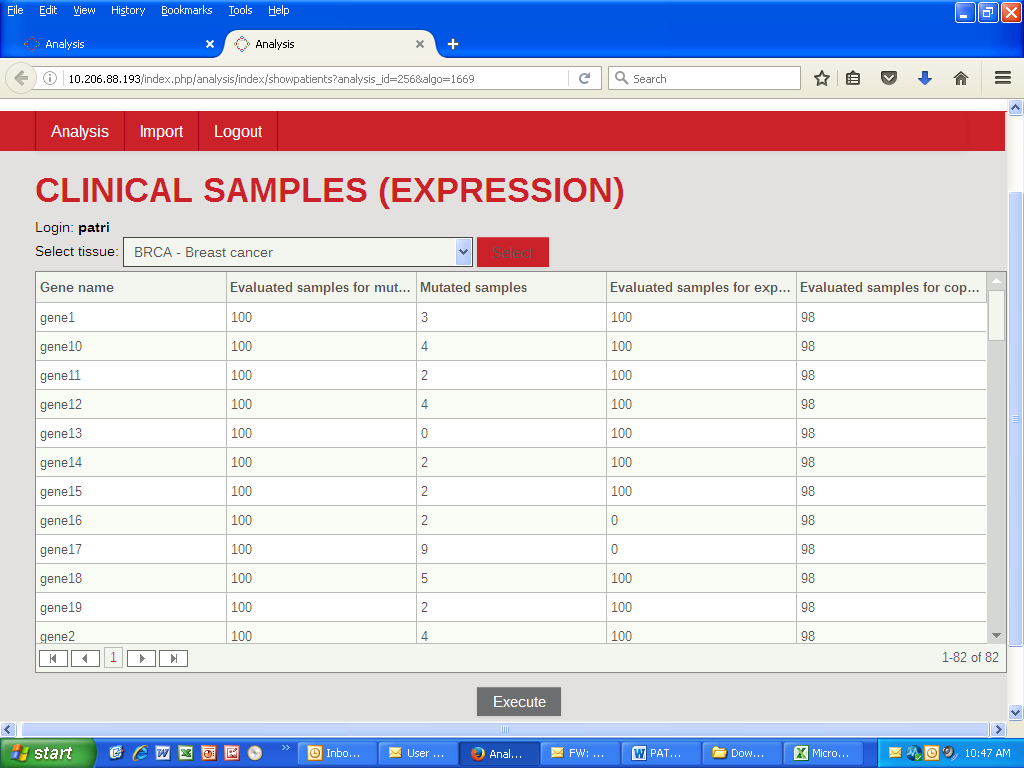


Fig. 20 –Clinical Sample (tumor tissue) selection before and after clicking the ‘Select’ button

Upon dataset selection, the analysis starts. The log of the run execution is printed on the screen (Fig. 21). When the execution of the program ended, the sentence Execution completed appears above the log screen in red characters.


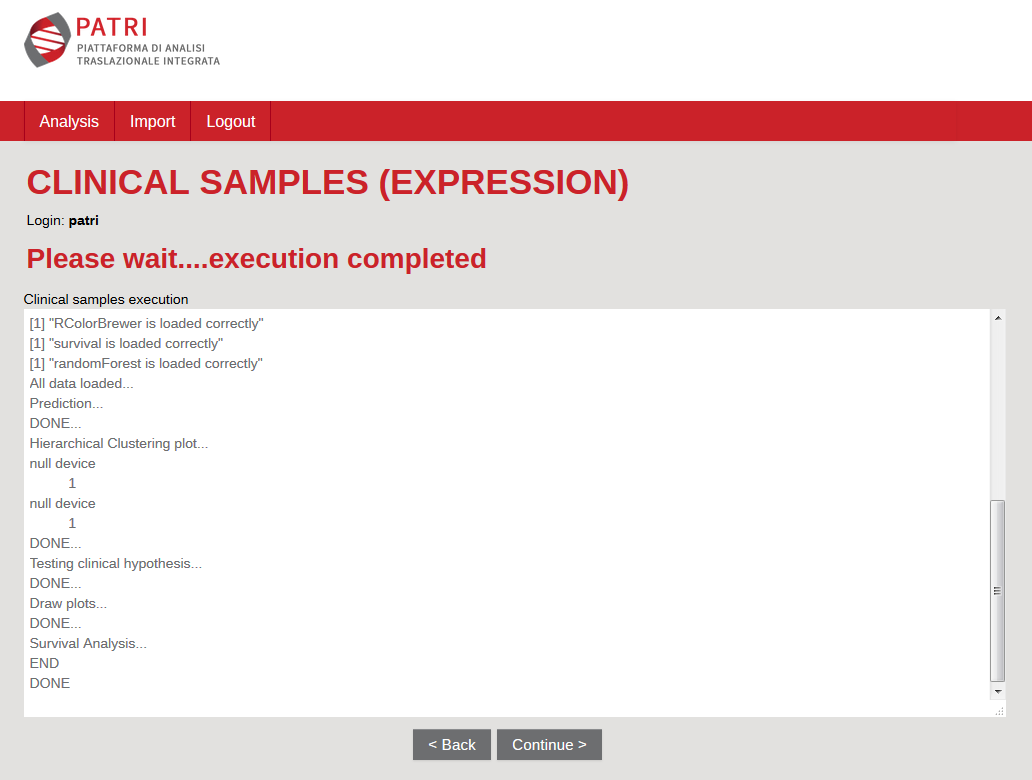


Fig. 21 - log Clinical Samples run

Statistically relevant clinical features, differentially characterizing the two subpopulations, are reported and can be graphically inspected, to support the exploration of novel hypotheses for potential treatment susceptibility criteria within the available patient population.

Fig. 22 - Clinical Samples (Expression) result page

The result page shown in Fig. 22 is organized as follows:

- A table reporting the statistically significant clinical features retrieved
- Two buttons for the generation of the Heatmap and the Kaplan-Meyer Curve respectively.
- Two buttons allowing Export as txt of the visualized table (Statistic table) and of the list of samples predicted as Sensitive or Resistant (Sample Description) along with the sample description annotations.

Table fields:

- Clinical_Feature = name of the field describing the clinical feature
- Type_of_Variable = type of variable (categorical or continuous)
- Classes (categorical) = names of the categories, only for categorical variables
- P_Value = p-value resulting from the analysis

By click on each single row a plot of sample distribution for the selected feature in potentially responder and non-responder to drug treatment is shown in PDF format. Below an example of categorical variable plot (bar chart) and continuous variable plot (box plot) is reported.


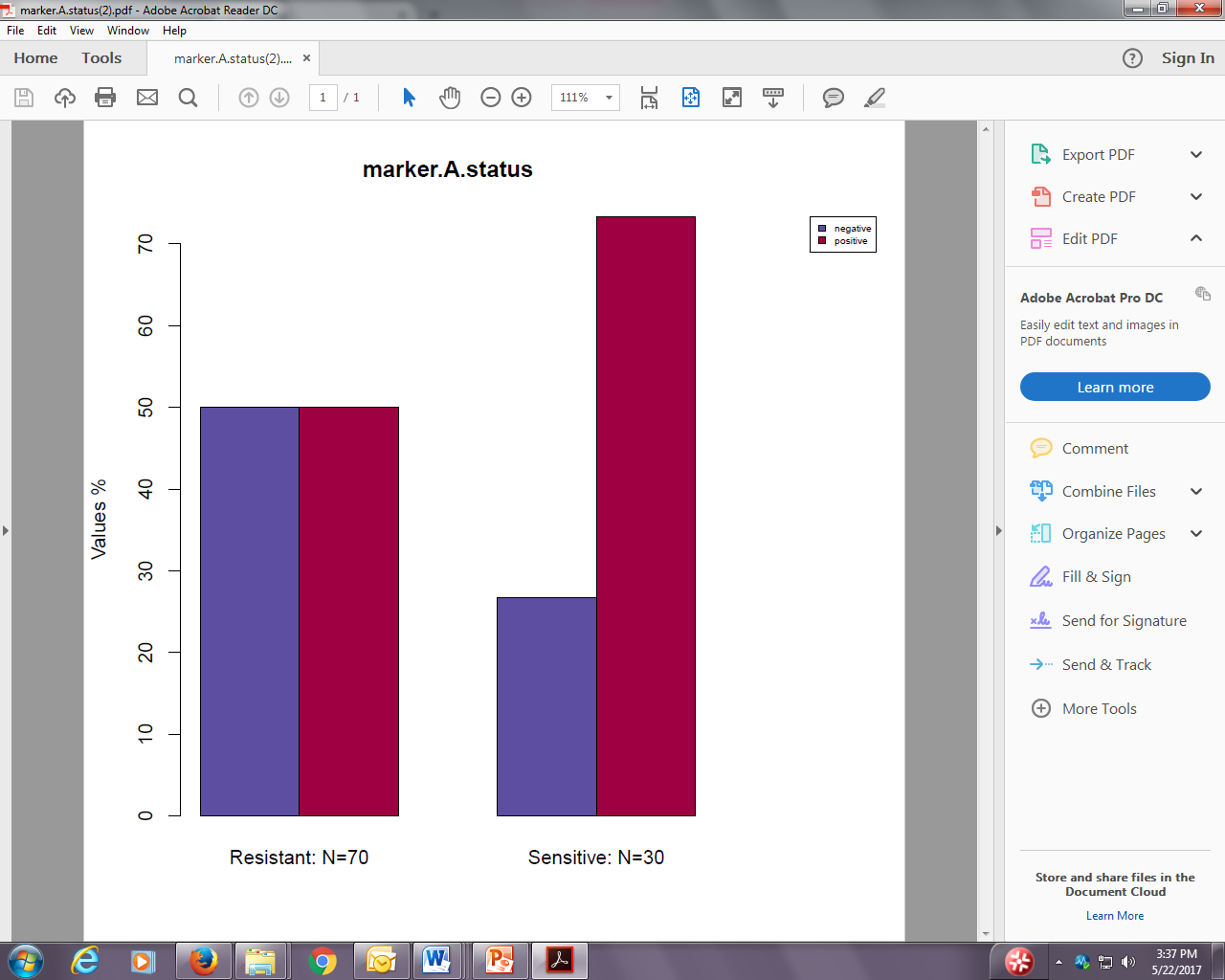


Fig. 23 – Example of a plot using categorical variables


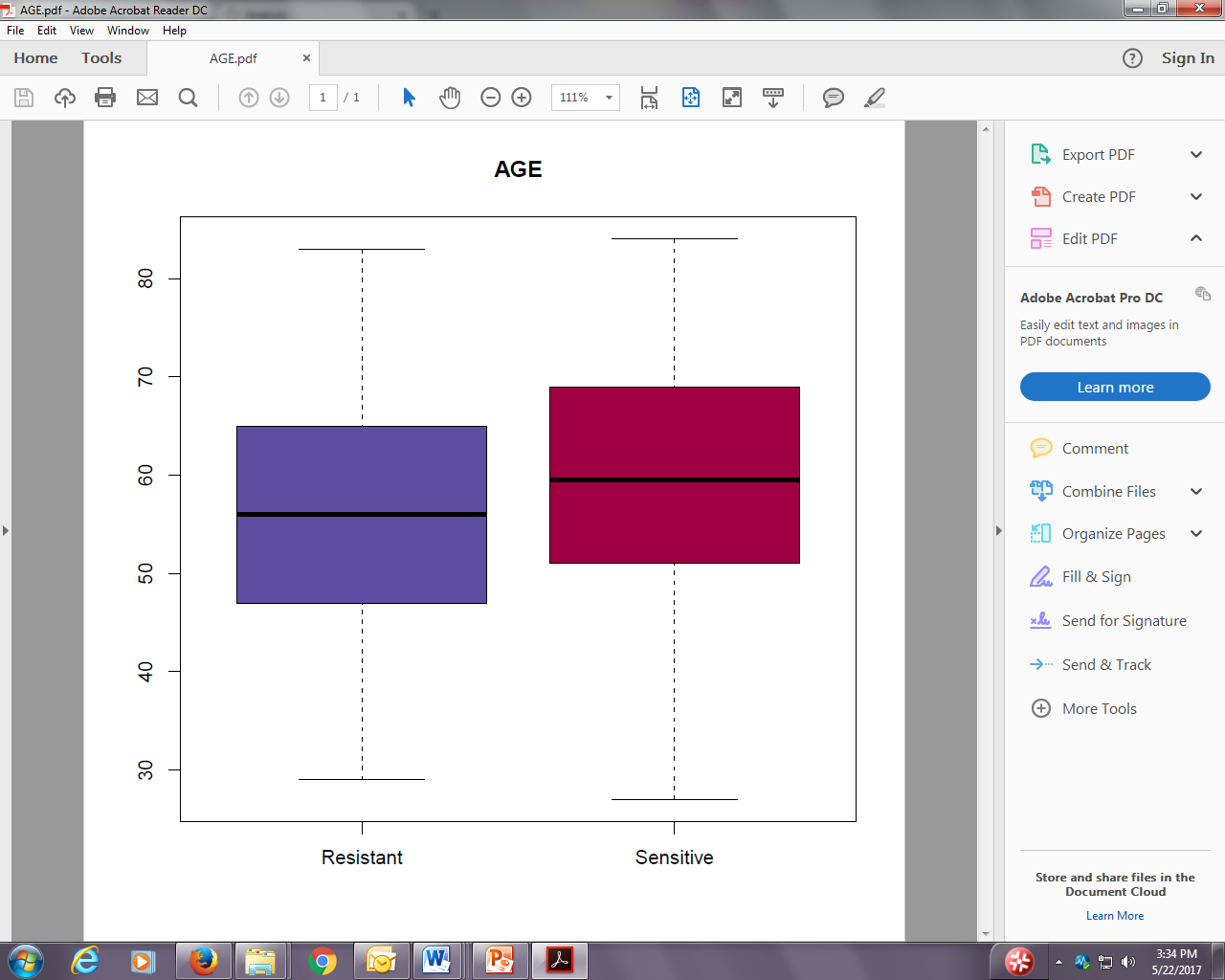


Fig. 24 - Example of a plot using continuous variables

The Heatmap button
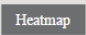
 generates a heatmap reporting the expression of the selected genes in the samples in PDF format (an example is shown in the figure below). Samples predicted as potentially sensitive to drug treatment are reported in blue, while potentially resistant samples are reported in green.


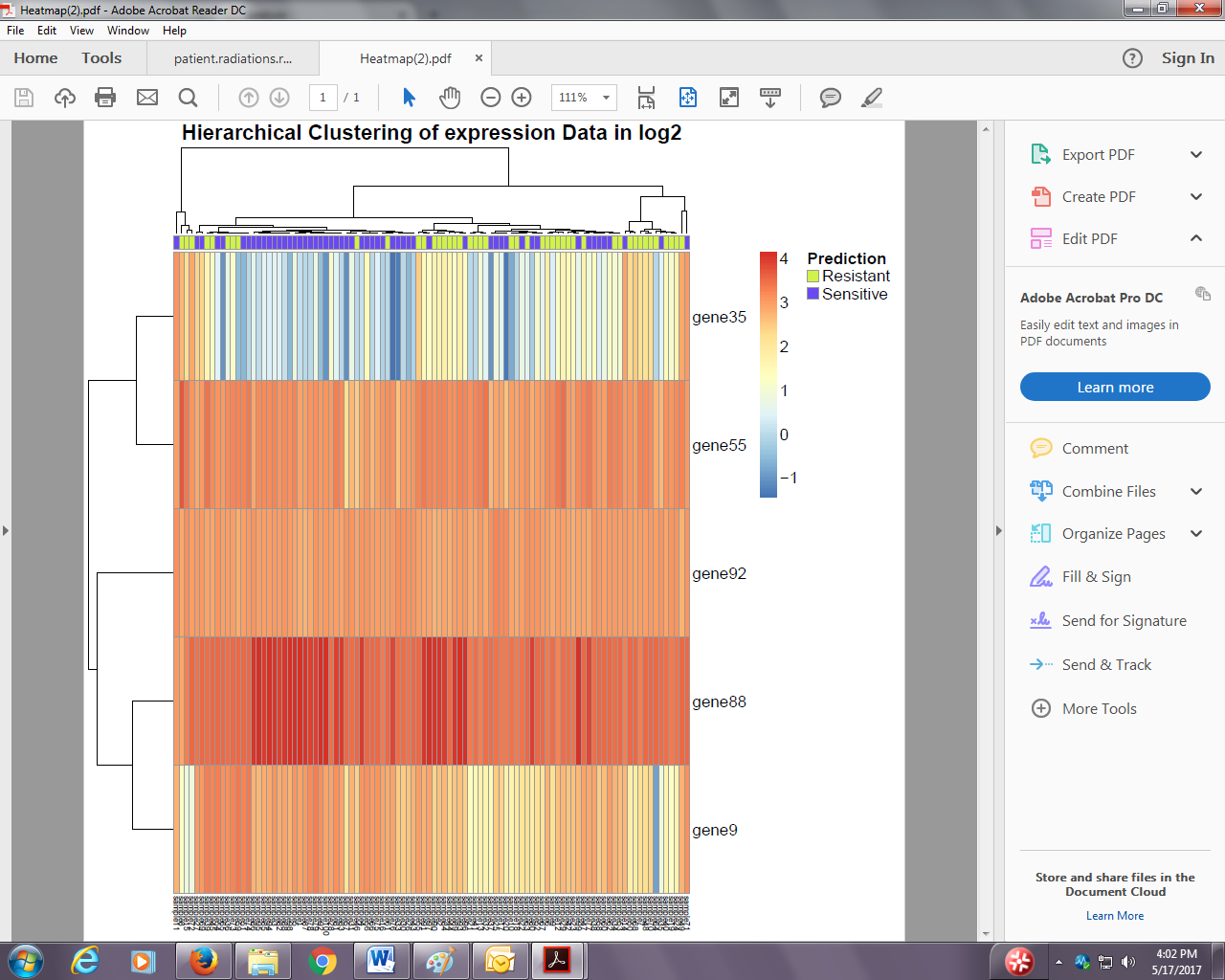


Fig. 25 – Heatmap

The KM_curve button displays Kaplan-Meier survival curve in PDF format (Fig. 26).


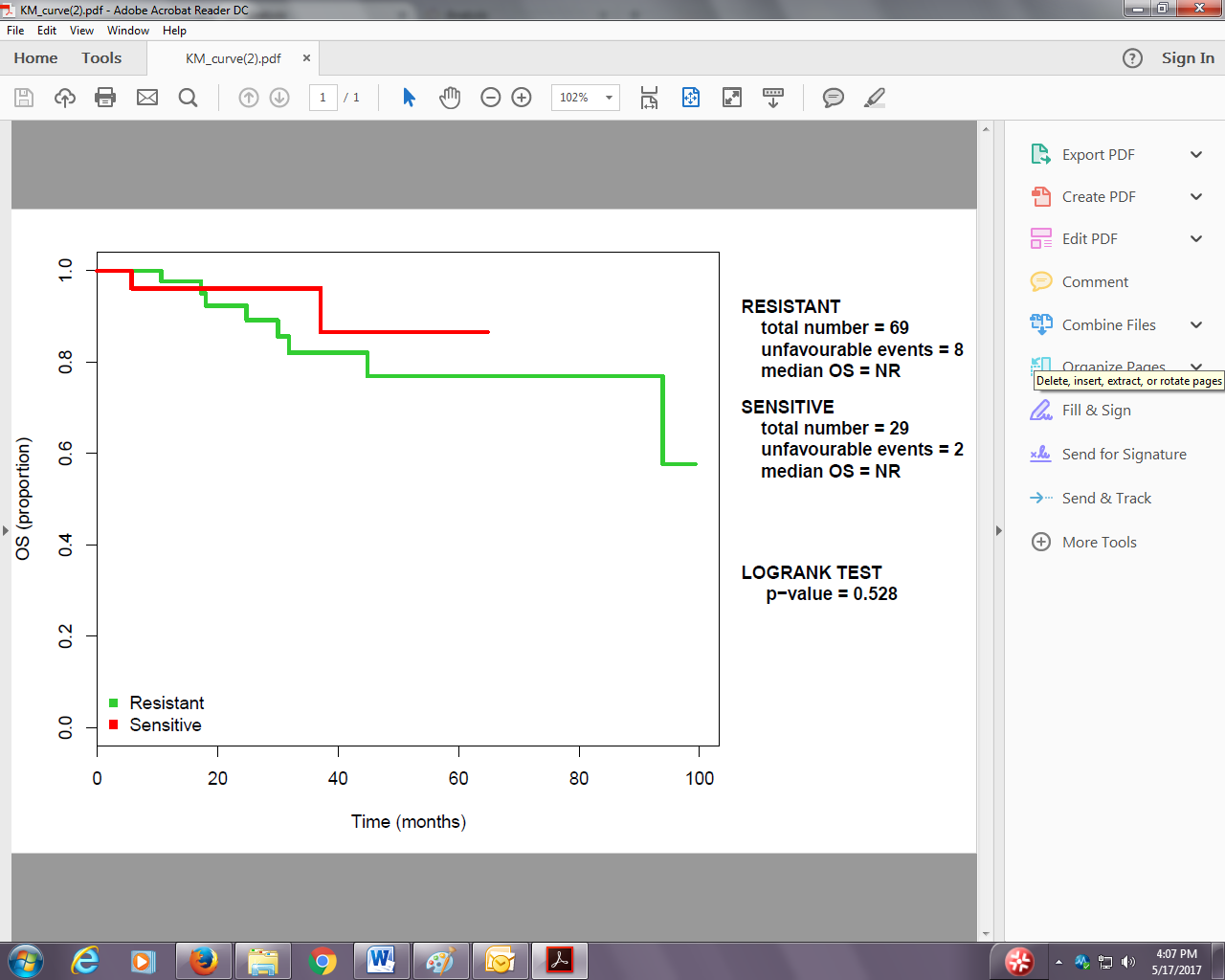


Fig. 26 - Kaplan-Meier curve

**New Sample Analysis**

Fig. 27- New Sample Analysis

The “New sample analysis” page can be accessed from the Analysis page either upon creation of a new analysis or by loading a previously saved one. It allows the selection of the clinical samples to include in the subsequent analyses.

The samples to be used in the analysis will be displayed in the **Selected samples table**.

Sample import can be performed in different ways:

1. Graphical selection:

- Select one or more samples from the provided table **Available samples** inserting the tick on the corresponding row
- Move cell lines from **Available samples** to **Selected samples** using the
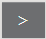
 button.

Note: The button
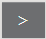
 allows moving the samples back to the **Available samples table**

Note: Using the buttons
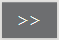
 and
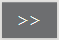
 the user can move from **Available samples** to **Selected samples** and vice versa all the samples.

1. Text selection:

- Click on **Import samples** button **
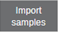
**
- Insert sample names delimited by space in the provided form
- Click **Import samples**

1. From file (including the label on sensitivity/resistance to the drug):

- Click on the **Import samples and sensitivity** button **
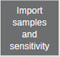
**
- Select a txt file (delimited by space) with sample names and the label “sensitive” or “resistant” (see the provided example files)
- Click **Import samples**

Note: Click on **Export Selection** button to download the list of samples included in the **Selected samples** table in txt format.

To continue with the analysis click on the button .

The **Delete Analysis** button on the top left allows removing the current analysis.

# New Sample Analysis: label of the samples

Fig. 28 – NEW SAMPLE ANALYSIS: selection of samples to be included in the analysis

In this page the user can label the sample as “sensitive” or “resistant”, if not done in the previous step, and eventually remove selected samples from the analysis by following the steps reported below:

- To assign the Sensitive (S) or Resistant (R) label to each sample, click on the red capital letter in the Sensitive/Resistant column
- The samples with a tick will be included in the subsequent analysis. Please deselect if you wish to exclude from the analysis. This field cannot be selected if the Sensitive/Resistant column value is **Null**.

Click on the following button to continue with the statistic model selection.

Table fields:

- Name of the sample
- Short name to describe the tumor
- **Sensitive/Resistant** column: it is mandatory to specify if the sample is considered sensitive or resistant to the drug treatment.
- Number of gene variants reported in the dataset loaded in PATRI for the indicated sample (**Mutated genes**).
- Number of WT genes reported in the dataset loaded PATRI for the indicated sample (**Wt genes**).
- Number of genes profiled by gene expression (**Genes evaluated for expression**) or Copy Number (**Genes evaluated for Copy Number**).

Please note:

1. At least 2 Resistant and 2 Sensitive samples must be selected to continue the analysis.

Elastic Net model (it can be selected in the following page) requires at least 4 Sensitive and 4 Resistant samples.

# New Sample Analysis: Algorithm choice

Please follow the same procedure described in ‘New Cell Analysis: Algorithm choice’

# Selected Sample Analysis

Fig. 29 - SELECTED SAMPLE ANALYSIS: Example of results from gene expression analysis by Limma

Genes identified in the differential analysis can be sorted by p-value or logFC (LR).

Please see ‘Selected Cell Analysis’ for details on filtering and sorting procedure.

The buttons below the result page allows to:

**Add genes**: add a gene not reported in the table because it doesn’t fit the statistics and filtering options set by the slider bars.

**Remove genes**: remove selected genes

**Charts**: open a new page allowing the selection of different chart types for data visualization.

**Full Export**: export of all results in txt format

**Tbl Export:** export of filtered data in txt format

Note: Chart visualization needs an active internet connection since **Charts** and **Volcano** use Google libraries to generate the plots.

# Data visualization by Charts

Please follow the same procedure described in ‘Charts’**.**

# Import

Fig. 30 – Import page menu

The Import function allows importing genomics data in PATRI platform. Data are requested in text format (space separator).

Click on the Import red button on the top bar.

Select the desired import dataset option from IMPORT list by clicking on the corresponding row (e.g. Import Expression for gene expression data) (Fig. 30).

Data format for each type of dataset is described below.

## Import Expression

Click on Import Expression link to upload gene expression dataset. The dataset has to be organized as follows:

| **Field Name** | **Description** | **Value** |
| --- | --- | --- |
| **SOURCE** | Data source | ie. ‘PUBLIC’; ‘INTERNAL’;… |
| **SAMPLE_TYPE** | Set = 1 if cell line, set = 0 if clinical sample | 0;1 |
| **SAMPLE_ID** | For cell line data: NULL;  For clinical samples data: equal to **SAMPLE_ID** in the **sample description** table | NULL; SAMPLE_ID |
| **CELL_NAME** (for cell lines)**/SHORT_TUMOR_TYPE** (for clinical samples) | For cell line data: name of the cell line (equal to **CELL_NAME** in **cell line description** table)  For clinical samples data: nickname of tumor type (equal to **SHORT_TUMOR_TYPE** in **sample description** table) | String |
| **GENE_NAME** | Gene Name | String |
| **PROBE_SET** | Probe set name (for microarray data only) | String |
| **VALUE** | Gene expression value (log2 format) | Float |

Table 1 - Gene Expression dataset fields

Note: For cell lines please set SAMPLE_TYPE and SAMPLE_ID as follows:

- SAMPLE_TYPE = 1
- SAMPLE_ID = NULL(or empty).

For clinical samples please set SAMPLE_TYPE and SAMPLE_ID as follows:

- SAMPLE_TYPE = 0
- SAMPLE_ID = SAMPLE_ID (from **sample description** table)

These two fields allow distinguishing Cell Lines from Clinical Samples Expression tables. Similarly for Mutation and CopyNumber tables.

For cell lines please provide a field CELL_NAME reporting the name of the cell line set equal to **CELL_NAME** in **cell line description** table.

For clinical samples provide instead a field SHORT_TUMOR_TYPE set equal to **SHORT_TUMOR_TYPE** in **sample description** table.

Example of gene expression dataset from cell lines:

| SOURCE | SAMPLE_TYPE | SAMPLE_ID | CELL_NAME |
| --- | --- | --- | --- |
| IN HOUSE | 1 | NULL | A2780 |
| IN HOUSE | 1 | NULL | A2780 |
| IN HOUSE | 1 | NULL | A2780 |
| IN HOUSE | 1 | NULL | A2780 |

| GENE_NAME | PROBE_SET | VALUE |
| --- | --- | --- |
| UBR5 | 51366_at | 8,66 |
| POP5 | 51367_at | 11,52 |
| PDE1A | 5136_at | 3,74 |
| POMP | 51371_at | 10,12 |

Table 2 - Gene Expression dataset (cell lines)

Example of gene expression dataset from clinical sample:

| SOURCE | SAMPLE_TYPE | SAMPLE_ID | SHORT_TUMOR_TYPE |
| --- | --- | --- | --- |
| HOSPITAL1 | 0 | Tumor_01 | CRC |
| HOSPITAL1 | 0 | Tumor_01 | CRC |
| HOSPITAL1 | 0 | Tumor_01 | CRC |
| HOSPITAL1 | 0 | Tumor_01 | CRC |

| GENE_NAME | PROBE_SET | VALUE |
| --- | --- | --- |
| UBR5 | 51366_at | 8,66 |
| POP5 | 51367_at | 11,52 |
| PDE1A | 5136_at | 3,74 |
| POMP | 51371_at | 10,12 |

Table 3 - Gene Expression dataset (cell lines)

## Import Mutation

Click on Import Mutation link to upload dataset containing gene variant data. The file has to be organized as follows:

| **Field Name** | **Description** | **Value** |
| --- | --- | --- |
| **SOURCE** | Data source | ie. ‘PUBLIC’; ‘INTERNAL’;… |
| **SAMPLE_TYPE** | Set = 1 if cell line, set = 0 if clinical sample | 0;1 |
| **SAMPLE_ID** | For cell line data: NULL;  For clinical samples data: equal to **SAMPLE_ID** in the **sample description** table | NULL; SAMPLE_ID |
| **CELL_NAME** (for cell lines)**/SHORT_TUMOR_TYPE** (for clinical samples) | For cell line data: name of the cell line (equal to **CELL_NAME** in **cell line description** table)  For clinical samples data: nickname of tumor type (equal to **SHORT_TUMOR_TYPE** in **sample description** table) | String |
| **GENE_NAME** | Gene Name | String |
| **MUT_OR_WT** | Specify if the gene is mutated (M) or not (WT) in this position | ‘W’ o ‘M’ |
| **AA_MUTATION** | Mutated AA position | String |
| **DNA_MUTATION** | Mutated nucleic acid position | String |
| **REFERENCE_ALLELE** | Expected nucleotide in the reference sequence | ‘A’, ’T’, ’C‘ o ‘G’ |
| **TUMOR_SEQ_ALLELE1** | Nucleotide in the Allele 1 | ‘A’, ’T’, ’C‘ o ‘G’ |
| **TUMOR_SEQ_ALLELE2** | Nucletide in the Allele 2 | ‘A’, ’T’, ’C‘ o ‘G’ |
| **DESCRIPTION** | Gene description | String |
| **MUTATION_DESCRIPTION** | Type of mutation | String |
| **CHROMOSOME** | Chromosome number | Integer |
| **START_POSITION** | Start Position | Integer |
| **END_POSITION** | End position | Integer |
|  |  |  |

Table4 - Mutation dataset fields

Example:

| SOURCE | SAMPLE_TYPE | SAMPLE_ID | CELL_NAME | GENE_NAME | MUT_OR_WT |
| --- | --- | --- | --- | --- | --- |
| PUBLIC | 1 | NULL | A2780 | ATR | M |
| PUBLIC | 1 | NULL | MCF7 | ATR | M |
| PUBLIC | 1 | NULL | COLO205 | ATR | M |
| PUBLIC | 1 | NULL | HELA | ATR | M |

| AA_MUTATION | DNA_MUTATION | REFERENCE_ALLELE | TUMOR_SEQ_ALLELE1 | TUMOR_SEQ_ALLELE2 |
| --- | --- | --- | --- | --- |
| p.Q43R | c.128A>G | A | G | G |
| p.R90Q | c.269G>A | G | A | A |
| p.R119C | c.355C>T | C | T | T |
| p.E126* | c.376G>T | G | T | T |

| DESCRIPTION | MUTATION_DESCRIPTION | CHROMOSOME | START_POSITION |
| --- | --- | --- | --- |
| Ataxia Telangiectasia And Rad3-Related Protein | Missense_Mutation | 7 | 98479625 |
| Ataxia Telangiectasia And Rad3-Related Protein | Missense_Mutation | 7 | 98490054 |
| Ataxia Telangiectasia And Rad3-Related Protein | Missense_Mutation | 7 | 98490140 |
| Ataxia Telangiectasia And Rad3-Related Protein | Nonsense_Mutation | 7 | 98491430 |

| END_POSITION |
| --- |
| 98479625 |
| 98490054 |
| 98490140 |
| 98491430 |

Table 5 – Example of Copy Number dataset

## Import Copy number

Click on Import Copy Number link to upload copy number datasets. The file has to be organized as follows:

| **Field Name** | **Description** | **Value** |
| --- | --- | --- |
| **SOURCE** | Data source | ie. ‘PUBLIC’; ‘INTERNAL’;… |
| **SAMPLE_TYPE** | Set = 1 if cell line, set = 0 if clinical sample | 0;1 |
| **SAMPLE_ID** | For cell line data: NULL;  For clinical samples data: equal to **SAMPLE_ID** in the **sample description** table | NULL; SAMPLE_ID |
| **CELL_NAME** (for cell lines)**/SHORT_TUMOR_TYPE** (for clinical samples) | For cell line data: name of the cell line (equal to **CELL_NAME** in **cell line description** table)  For clinical samples data: nickname of tumor type (equal to **SHORT_TUMOR_TYPE** in **sample description** table) | String |
| **GENE_NAME** | Gene Name | String |
| **CHROMOSOME** | Chromosome number | String |
| **START** | Transcription Start Position | Integer |
| **END** | Transcription End Position | Integer |
| **COPY_NUMBER** | Copy Number Value (linear format) | Float |
| **TYPE** | Loss, normal or gain (user defined) | String |

Table 6 - Copy Number dataset fields

Example:

| SOURCE | SAMPLE_TYPE | SAMPLE_ID | CELL_NAME | GENE_NAME |
| --- | --- | --- | --- | --- |
| LAB1 | 1 | NULL | A2780 | WASH7P |
| LAB1 | 1 | NULL | A2780 | FAM138F |
| LAB1 | 1 | NULL | A2780 | OR4F5 |
| LAB1 | 1 | NULL | A2780 | ISG15 |

| NUMCHR | TXSTART | TXEND | COPY_NUMBER | TYPE |
| --- | --- | --- | --- | --- |
| 1 | 14363 | 29961 | 2 | NORMAL |
| 1 | 34567 | 36081 | 4 | GAIN |
| 1 | 63555 | 70008 | 4 | GAIN |
| 1 | 321274 | 325876 | 1 | LOSS |

Table 7 – Example of Copy Number dataset

## Import Cell Line Description

Click on the Import Cell Line Description link to upload a tab delimited file containing information on the cell lines used in the study. The file has to be organized as follows:

| **Field Name** | **Description** | **Value** |
| --- | --- | --- |
| **SOURCE** | Data source | ie. ‘PUBLIC’; ‘INTERNAL’;… |
| **CELL_NAME** | Name of the cell line | String |
| **ID_REF** | Reference ID (ie. GEO access number or internal reference ID) | String |
| **PROVIDER** | Cell line provider (ie. ATCC, DSMZ, RIKEN, …) | String |
| **ORGANISM** | Organism from which the cell line was derived (er. human, mouse) | String |
| **PRIMARY_SITE** | Tissue of origin of the cell line | String |
| **HISTOLOGY** | Histology | String |
| **HISTOLOGY_SUBTYPE** | Histology subtype | String |
|  |  |  |

Table 8 - Cell Line dataset fields

Example:

| **SOURCE** | **CELL_NAME** | **ID_REF** | **PROVIDER** |
| --- | --- | --- | --- |
| GEO | A2780 | GSM887008 | ATCC |
| GEO | MCF7 | GSM887009 | DSMZ |
| GEO | COLO205 | GSM887010 | DSMZ |
| GEO | HELA | GSM887011 | ATCC |

| **ORGANISM** | **HISTOLOGY** | **HISTOLOGY_SUBTYPE** |
| --- | --- | --- |
| Homo sapiens | carcinoma | Clear cell carcinoma |
| Homo sapiens | leukemia | Chronic myeloid leukemia |
| Homo sapiens | leukemia | Acute myeloid leukaemia |
| Homo sapiens | carcinoma | Squamous cell carcinoma |

Table 9 – Example of Cell Line description dataset

## Import Gene Description

Click on Import Gene Description to import the dataset of gene annotation. The file has to be organized as follows:

| **Field Name** | **Description** | **Value** |
| --- | --- | --- |
| **SOURCE** | Data source | ie. ‘NCBI RefSeq’ |
| **GENE_NAME** | Gene Name | String |
| **GENE_ID** | Gene ID | String |
| **ID_REF** | Reference ID (ie. GEO access number or internal reference ID) | String |
| **DESCRIPTION** | Gene Description | String |
| **CHROMOSOME** | Chromosome number | Integer |
| **START** | Gene Start Position | Integer |
| **END** | Gene End Position | Integer |

Table 10 – Gene description dataset fields

Example:

| SOURCE | GENE_NAME | GENE_ID | ID_REF |
| --- | --- | --- | --- |
| RefSeq | APBA2 | NULL | NULL |
| RefSeq | APC | NULL | NULL |
| RefSeq | APEX1 | NULL | NULL |
| RefSeq | API5 | NULL | NULL |

| DESCRIPTION | CHROMOSOME | START | END |
| --- | --- | --- | --- |
| amyloid beta (A4) precursor protein-binding; | 7 | 98479625 | 98479625 |
| adenomatous polyposis coli | 1 | 98490054 | 98490054 |
| APEX nuclease 1 | 11 | 98490140 | 98490140 |
| apoptosis inhibitor 5; | 9 | 98491430 | 98491430 |

Table 11 –Example of gene description dataset

## Import Clinical Sample Description

The “clinical sample_description” table is designed to store patient clinical data and contains 8 mandatory fields:

- sample identifier
- tumor type
- short abbreviation of tumor type
- gender
- age
- days to death
- days to last follow up
- vital status

The last 3 fields are used to generate Kaplan-Meier (KM) survival curves.

In addition 200 generic fields (from GEN1 to GEN200) are provided to allow customization based on the specific tumor type and on specific institution or research center clinical data.

Two separate tables are required: a table called Clinical Samples Description, with information on the clinical sample, and a Clinical Samples Description Mapping table, with description of the GEN1-GEN200 fields, that will be cross-linked to the first table.

First click on Import Clinical Samples Description to upload clinical sample information. The file has to be organized as follows:

| **Field Name** | **Description** | **Value** |
| --- | --- | --- |
| SAMPLE _ID | sample identifier | String |
| TUMOR_TYPE | Tumor description | String |
| SHORT_TUMOR_TYPE | Short description of tumor type. | String |
| GENDER |  | String |
| AGE |  | Number |
| DAYSTODEATH |  | Number |
| DAYSTOLASTFOLLOWUP |  | Number |
| VITALSTATUS |  | String |
| GEN1 | In this and the following fields, user-defined clinical features can be defined, i.e. Marker A status | String |
| GEN2 | See above | String |
| .......... | See above | String |
| GEN200 | See above | String |

Table 12 - Clinical Samples Description table

Example:

| SAMPLE_ID | TUMOR_TYPE | SHORT_TUMOR_TYPE | GENDER | AGE |
| --- | --- | --- | --- | --- |
| Sample1 | Breast cancer | brca | F | 54 |
| Sample2 | Breast cancer | brca | M | 84 |
| Sample3 | Breast cancer | brca | M | 78 |
| Sample4 | Breast cancer | brca | F | 71 |
|  |  |  |  |  |
| DAYSTODEATH | DAYSTOLASTFOLLOWUP | VITALSTATUS | GEN1 | GEN2 |
| 548 | NA | dead | negative | african |
| NA | 1119 | alive | negative | european |
| 624 | NA | dead | negative | african |
| NA | 1847 | alive | positive | european |

Table 13 – Example of Sample Description dataset

Then click on Import Clinical Samples Description Mapping to upload information on generic field and their link to Sample Description table. The file has to be organized as follows:

| **Field Name** | **Description** | **Value** |
| --- | --- | --- |
| **SHORTTUMORTYPE** | Short description of tumor type. It is linked to short_tumor_type field in SamplesDescription table | String |
| **TUMORTYPE** | Tumor description | String |
| **POSITION** | Insert the column number (*) corresponding tothe SamplesDescription table | String |
| **FIELD_DESCRIPTION** | Replace GEN1…GEN2 with a description of the clinical parameters under evaluation | String |

Table 14 - Clinical Samples Description Mapping Table

* ie.: POSITION = 1 corresponds to the column SAMPLE _ID; POSITION = 2 to the column TUMOR_TYPE; ... POSITION = 9 to the column marker A status, POSITION = 10 to the column marker B status; ...

Example:

|  | SHORTTUMORTYPE | TUMORTYPE | POSITION | FIELD_DESCRIPTION |
| --- | --- | --- | --- | --- |
|  | brca | Breast cancer | 1 | SAMPLE _ID |
|  | brca | Breast cancer | 2 | TUMOR_TYPE |
|  | brca | Breast cancer | 3 | SHORT_TUMOR_TYPE |
|  | brca | Breast cancer | 4 | GENDER |
|  | brca | Breast cancer | 5 | AGE |
|  | brca | Breast cancer | 6 | DAYSTODEATH |
|  | brca | Breast cancer | 7 | DAYSTOLASTFOLLOWUP |
|  | brca | Breast cancer | 8 | VITALSTATUS |
|  | brca | Breast cancer | 9 | marker A status |
|  | brca | Breast cancer | 10 | marker B status |
|  | brca | Breast cancer | 11 | country of originN3 |

Table 15- Example of Sample Description Mapping dataset
